# Distinct community structures of soil nematodes from three ecologically different sites revealed by high-throughput amplicon sequencing of four 18S ribosomal RNA gene regions

**DOI:** 10.1371/journal.pone.0249571

**Published:** 2021-04-15

**Authors:** Harutaro Kenmotsu, Masahiro Ishikawa, Tomokazu Nitta, Yuu Hirose, Toshihiko Eki

**Affiliations:** 1 Molecular Genetics Laboratory, Department of Applied Chemistry and Life Science, Toyohashi University of Technology, Toyohashi, Aichi, Japan; 2 Research Center for Agrotechnology and Biotechnology, Toyohashi University of Technology, Toyohashi, Aichi, Japan; University of Limpopo, SOUTH AFRICA

## Abstract

Quantitative taxonomic compositions of nematode communities help to assess soil environments due to their rich abundance and various feeding habitats. DNA metabarcoding by the 18S ribosomal RNA gene (SSU) regions were preferentially used for analyses of soil nematode communities, but the optimal regions for high-throughput amplicon sequencing have not previously been well investigated. In this work, we performed Illumina-based amplicon sequencing of four SSU regions (regions 1–4) to identify suitable regions for nematode metabarcoding using the taxonomic structures of nematodes from uncultivated field, copse, and cultivated house garden soils. The fewest nematode-derived sequence variants (SVs) were detected in region 3, and the total nematode-derived SVs were comparable in regions 1 and 4. The relative abundances of reads in regions 1 and 4 were consistent in both orders and feeding groups with prior studies, thus suggesting that region 4 is a suitable target for the DNA barcoding of nematode communities. Distinct community structures of nematodes were detected in the taxon, feeding habitat, and life-history strategy of each sample; i.e., Dorylamida- and Rhabditida-derived plant feeders were most abundant in the copse soil, Rhabditida-derived bacteria feeders in the house garden soil, and Mononchida- and Dorylamida-derived omnivores and predators and Rhabditida-derived bacteria feeders in the field soil. Additionally, low- and high-colonizer–persister (cp) groups of nematodes dominated in the house garden and copse soils, respectively, whereas both groups were found in the field soil, suggesting bacteria-rich garden soil, undisturbed and plant-rich copse soil, and a transient status of nematode communities in the field soil. These results were also supported by the maturity indices of the three sampling sites. Finally, the influence of the primer tail sequences was demonstrated to be insignificant on amplification. These findings will be useful for DNA metabarcoding of soil nematode communities by amplicon sequencing.

## Introduction

Many soil-dwelling organisms, invisible to the naked eye, form stable ecosystems and play important roles in nutrient recycling in the pedosphere [1]. Nematodes are abundant in soil, are widely distributed in freshwater, terrestrial, and marine environments on Earth [2–5], and exhibit a variety of feeding habits, such as bacterial and fungal feeding, predation, and animal or plant parasitism [6]. Nematodes play a crucial role in the nutrient cycling of soil biota and influence plant growth [7]. Nematode taxonomic compositions vary by ecosystem type [8, 9] and are influenced by various factors such as food availability and abundance, physical and chemical parameters (e.g., pH, temperature) [10, 11], soil properties [11, 12], and agricultural conditions (e.g., tillage, cultivated plants, fertilizers) [13–22]. These characteristics strongly indicate that the taxonomic composition and abundance of nematodes can be used as an indicator for biological conditions in soils [2, 23–25]. Prior research by our laboratory has demonstrated the presence of distinct nematode taxonomic compositions in soybean-cultivated agricultural and unmanaged follower bed soils using DNA barcoding by Sanger sequencing of the 900-base pair (bp) region of the 18S small subunit ribosomal RNA (SSU) gene from individual nematodes [26]. During DNA barcoding of nematodes, the nucleotide sequences of polymerase chain reaction (PCR)-amplified barcode DNAs from individual nematodes were determined by DNA sequencing; the nematodes were then classified by their sequences into taxonomic groups sharing identical DNA barcode sequences, known as operational taxonomic units (OTUs). The number of OTUs indicates the number of taxonomic groups (i.e., nematode species diversity), and the number of nematodes in each OTU shows the proportion of each taxonomic group in the nematode community. Thus, this process provides quantitative and qualitative information on the nematode community. However, the “one-by-one DNA barcoding” method using Sanger sequencing of a PCR amplicon from an individual organism is laborious and time-consuming, and the resulting taxonomic data is often poor due to a limited number of nematode samples [27]. To speed up the process, high-throughput sequencing-assisted DNA barcoding has been applied to the taxonomic analysis of soil nematodes. To find suitable regions for DNA barcoding among four SSU gene regions (regions 1–4, 337–388 bp in length), the present authors [28] clarified the taxa of 68 out of 96 individual nematodes isolated from copse soil using massive amplicon sequencing via the Illumina MiSeq platform. Region 4, located at the 3’-region of the SSU gene, was suggested as the most suitable among these four regions due to the identification of the most abundant nematode-derived sequence variants (SVs) and sufficient reference sequence coverage in DNA barcoding of individual nematodes; however, it is unclear whether these advantages are applicable for DNA barcoding in nematode communities (i.e., involving complex mixed nematodes). Although there have been several studies on the DNA metabarcoding of terrestrial nematodes using high-throughput sequencing of SSU gene-derived amplicons [20, 29–33], the regions most suitable for complex nematode communities have yet not been identified.

Recent progress in DNA metabarcoding using “environmental DNA” has allowed researchers to clarify species and their abundance living in various environments [34]. In these DNA metabarcoding experiments, the proper barcode in environmental DNAs derived from living organisms has been specifically amplified by PCR, and their nucleotide sequences have been determined by high-throughput sequencing to assign their taxa and abundances based on the resultant huge number of sequences. This work therefore aims to apply this technology to the taxonomic profiling of soil nematodes. SSU and cytochrome *c* oxidase I (COI) gene-derived sequences have been used as representative barcodes for DNA metabarcoding [35]. DNA barcoding with COI sequences provides high taxonomic resolution, but it is difficult to develop universal primers for this method to cover a variety of specie, as these nucleotide sequences have diverged even in isolated [26] and mock nematode communities [36]. Universal SSU primers have been developed for the DNA barcoding of eukaryotes via high-throughput amplicon sequencing [37, 38]. Since the amplicons generated by these universal primers contain more heterogenous eukaryotes than those with other primers such as COI, SSU primers could be useful for DNA metabarcoding. The resultant taxonomic resolutions from the amplicon sequencing with universal primers were not significant due to the relatively high sequence conservation of the SSU genes [35]. However, it is crucial in order to conduct taxonomic analysis of soil nematode communities by high-throughput amplicon sequencing, to develop PCR primers that can amplify SSU gene-derived DNAs containing relatively diverged nucleotide sequences from a wide variety of nematode species. In this work, therefore, Illumina MiSeq-assisted amplicon sequencing of four SSU regions is performed using different nematode community-derived DNA to clarify the taxonomic profile of each nematode community and to investigate optimal SSU regions for DNA barcoding of heterogeneous nematode populations. In addition, tailed PCR primers for the Illumina sequencing platform were often used to prepare amplicons for DNA barcoding, however, the effects of tail sequences on the results have not been well investigated. Thus, we also examined their impacts by comparing the results from the high-throughput sequencing of amplicons prepared using one-step PCR with tailed primers and two-step PCR with tailless and tailed primers.

## Materials and methods

### Soil sampling

**Fig 1:**
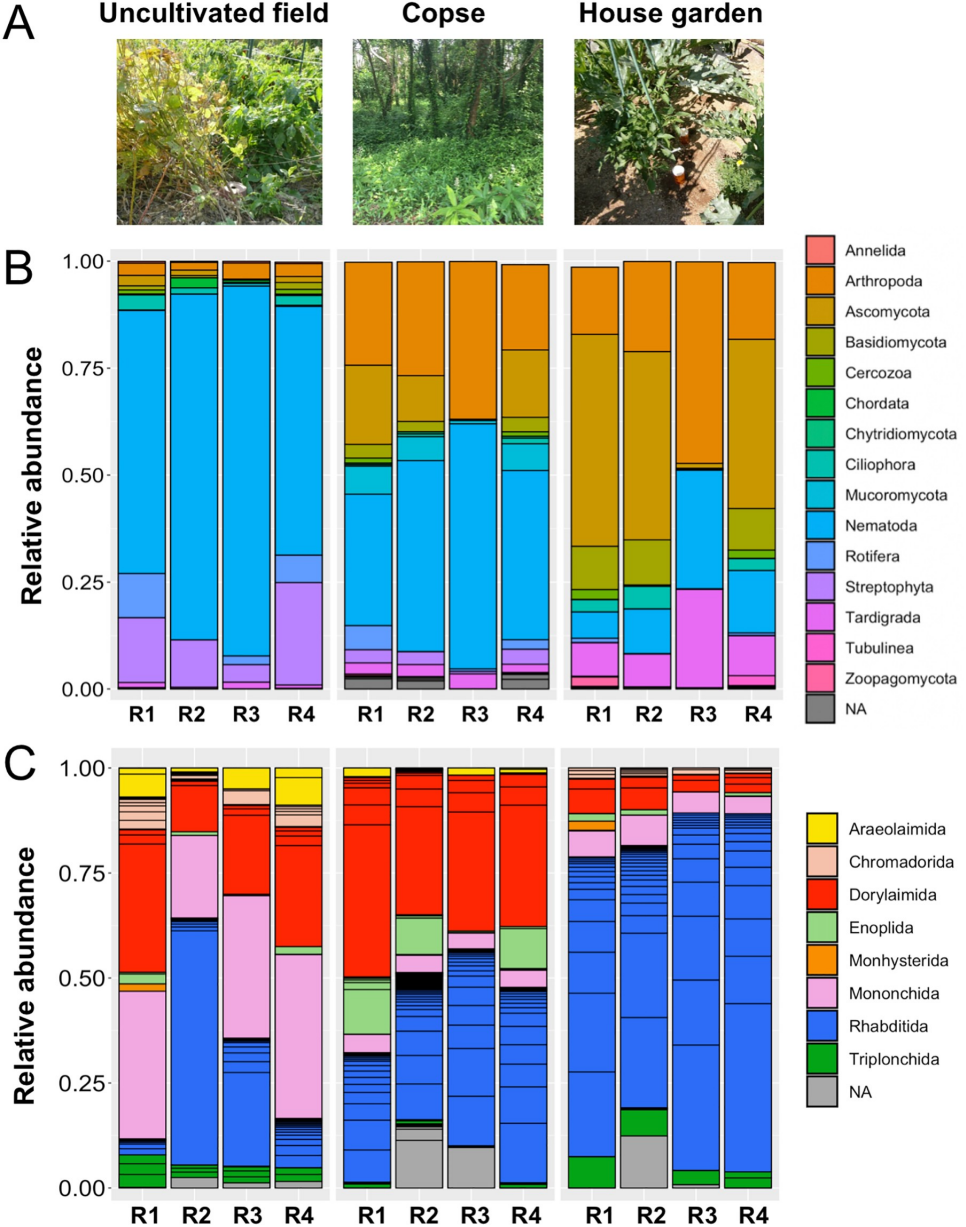
Sampling sites and relative abundances of sequence variants (SVs) at the phylum level and the nematode order level. (A) Soil sampling sites. Photographs of uncultivated field and copse are reprinted from prior PLoS One papers [26, 28]. (B) Relative abundance and phylum of SVs obtained from regions 1 (R1), 2 (R2), 3 (R3), and 4 (R4) of the 18S ribosomal RNA (SSU) gene in each sample. (C) Relative abundance and order of nematode-derived SVs from each region (R1–R4). Each sample was derived from the sampling site in (A) on top of histograms in (B) and (C). Boxes in the histogram indicate the SVs and their abundances; phylum and order levels are indicated by color. Taxonomic classification of SVs was based on the SILVA database [46]. NA: Not assigned.

Soil samples were collected from a copse on Toyohashi University of Technology campus, as in a prior study [28], and from a zucchini-cultivated house garden where chemical fertilizers and water were periodically supplied in June and July 2016 under clear climatic conditions (temperature: 28°C–35°C) in Toyohashi, Japan (137° 24’E, 34° 42’N), which is located in the temperate zone (Fig 1A). A field soil sample was collected in November 2013 from an uncultivated field managed by the Research Center for Agrotechnology and Biotechnology of the Toyohashi University of Technology, as detailed in the prior work [26] (Fig 1A). The soil was sampled to a depth of 15–20 cm using a soil sampling auger (Fujiwara Scientific Co., Tokyo, Japan) 2.5 cm in diameter. Three independent soil samples were taken and mixed from each site. Over-sized contaminants (e.g., stones and plant roots) were removed by filtering the samples through a 0.7 mm sieve; the resulting samples (approximately 40 g of fresh soil in total) were used for nematode isolation within a day of collection.

### Isolation of nematodes and DNA preparation

Nematodes from approximately 10-g samples of fresh soil from a copse and a house garden were isolated using the improved flotation sieving method with colloidal silica, as described by Morise et al. [26], and repeated four times for each sample location. Whole nematodes from approximately 40-g soil samples were thus trapped on sieves and eluted into water in a watch glass; the nematodes were then picked up using a micropipette (P-20, Gilson, Middleton, WI, USA) with a cut tip under an SZX16 stereomicroscope (Olympus) and collected into a DNA LoBind tube (Eppendorf, Hamburg, Germany). Genomic DNAs from whole nematodes isolated from copse and house garden soils were purified using DNeasy PowerSoil Kit (QIAGEN, Venlo, Netherlands) according to the manufacturer’s instructions. Single nematodes isolated from the field soil were then transferred to a tube, heated at 99°C for 3 min in 20 μL of 0.25 M NaOH, and then neutralized by adding 4 μL of 1 M HCl, 10 μL of 0.5 M Tris-HCl (pH 8.0), and 5 μL of 2% Triton X-100. Fifty-five nematode DNA samples were mixed and stored at –20°C until use in the following PCR experiment.

### PCR and DNA sequencing

Four sets of PCR primers with or without tail sequences for Illumina MiSeq sequencing were obtained from Fasmac (Atsugi, Japan) (detailed in Table 1). PCR primers without tail sequences were only used for the two-step PCR as described below. PCR primers with tail sequences were used for amplification of the 18S SSU gene fragments in the corresponding regions 1–4 (see S1 Fig), as detailed by Kenmotsu et al. [28]. The PCR reaction mixture (20 μL) contained 10 μL of 2 × Buffer for KOD FX Neo, 4 μL of 2 mM dNTPs, 0.4 units of the KOD FX Neo DNA polymerase (Toyobo, Tokyo, Japan), 2 μL of nematode DNA, as described above, and 0.3 μM each of the tailed forward and reverse primers. Amplification from the template DNA of nematodes from copse and house garden soils was initiated with a 2-minute denaturation at 94°C followed by 30 cycles each involving denaturation at 94°C for 10 s, annealing at 55°C for 30 s, and extension at 68°C for 60 s. PCR amplification using DNA from field soil-derived nematodes was performed in two ways to investigate the influence of tail sequences on PCR: (1) with tailed primers as described above except with 35 cycles (i.e., one-step PCR), and (2) PCR with tailless primers, which involves 27 cycles as described above, followed by 8 cycles of PCR with the corresponding tailed primers (i.e. two-step PCR). After confirming the PCR products in 1% or 2% agarose gel electrophoresis, they were purified with 0.8 volumes of AMPure XP beads (Beckman-Coulter, Indianapolis, IN, USA) according to the manufacturer’s instructions and eluted with 10 mM Tris-HCl (pH 8.5). Index PCR was performed in a thermocycler for eight cycles using a Nextera XT Index Kit v2 (Illumina, San Diego, CA, USA) according to the manufacturer’s instructions. The independent index was used for the 18S rRNA amplicon that was obtained from each reaction. The amplified libraries were purified by the addition of 1.12 volumes of AMPure XP beads and eluted with 10 mM Tris-HCl (pH 8.5). The concentration of each library was quantified using a spectrophotometer. Equal amounts of the libraries were pooled and quantified using a Qubit dsDNA HS Assay Kit (Thermo Fisher Scientific, Waltham, MA, USA). Each 300 bp end of the pooled library was sequenced using a MiSeq Reagent Kit v3 (600 cycles; Illumina) on a MiSeq instrument (Illumina). The sequences were deposited in the DDBJ Sequence Read Archive database under the accession number DR011293 with BioProject ID PRJDB10960 and BioSample IDs SAMD00264441 to SAMD00264443. The details of the registered data were shown in S1 Table.

**Table 1:** Polymerase chain reaction (PCR) primers used for amplifying the small subunit ribosomal RNA (SSU) gene regions.

SSU gene region^a^	Primer	Nucleotide sequence (5′-to-3′)^b^	Amplicon size (bp)^c^
Region 1	SSU18A-4F3	GCTTRTCTCAAAGATTAAGCCATGCATG	388
	SSU_R22	GCCTGCTGCCTTCCTTGGA	
Region 2	SSUconsF1	AGCAGCCGCGGTAATTCCAGCTC	384
	SSU26Rplus4	AAGACATTCTTGGCAAATGCTTTCG	
Region 3	Nem_18SR_ExtF	GTTCGAAGGCGATYAGATACCGCC	337
	SSU_R23plus7	TCGYTCGTTATCGGAATWAACCAGAC	
Region 4	NF1	GGTGGTGCATGGCCGTTCTTAGTT	368
	18Sr2b_ExtR	GGTGTGTACAAAKSGCAGGGACGTA	

^a^ These regions are detailed by Kenmotsu et al. [28] and in S1 Fig.

^b^ The corresponding primers with tail sequence were also used by Kenmotsu et al. [28]. Except for SSU_R22 [39], NF1 [29] and the newly generated SSUconsF1, the nucleotide sequences of the primers were modified from the following original primers based on the multiple alignment of the publicly available nematode SSU nucleotide sequences: SSU18A (5′-AAAGATTAAGCCATGCATG-3′) [40] for SSU18A-4F3, Nem_18S_F (5′-CGCGAATRGCTCATTACAACAGC-3′) [41] for Nem_18SR_ExtF, SSU_R_26 (5′-CATTCTTGGCAAATGCTTTCG-3′) and SSU_R_23 (5′-TCTCGCTCGTTATCGGAAT-3′) [39] for SSU26Rplus4 and SSU_R23plus7, and 18Sr2b (5′-TACAAAGGGCAGGGACGTAAT-3′) [29] for 18Sr2b_ExtR, respectively.

^c^ The predicted length of amplicon generated from *C*. *elegans* genomic DNA is indicated in S1 Fig.

### DNA sequence data analysis

The sequence reads from four amplicons from PCRs with tailed and tailless primers (field sample) and from PCRs with tailed primers (copse and house garden samples) were independently imported into QIIME2 version 2020.2 (https://qiime2.org) [42]. The primer sequences were removed by Cutadapt plugin (version 3.1) with the default parameters [43]. The forward and reverse reads were joined, denoised, and chimera-checked using the dada2 plugin [44]. We utilized the trimming parameters (—p-trunc-len-f and—p-trunc-len-r) of 220, 220 (region 1), 225, 200 (region 2), 220, 220 (region 3), and 220, 220 (region 4), respectively, and utilized the default parameters for the other options. To perform reference-based detection and removal of the chimeric sequences, the resultant SVs from dada2 [44] were further processed with vsearch uchime ref command with a minimum score option of—minh 0.5 using VSEARCH (version 2.13.3) [45] and the 18S rRNA reference from the SILVA database, version 132, with a 99% clustering threshold (https://www.arb-silva.de/download/archive/) [46]. Finally, the chimera-romoved SVs were filtered according to length as follows: 345 bp (region 1), 340 bp (region 2), 280 bp (region 3), and 310 bp (region 4), to remove short SVs that were derived from the amplicon PCR. The taxonomy of the SVs was assigned using a feature-classifier plugin that was trained with the 99% clustered 18S rRNA references in the SILVA database [46]. The taxonomic ranks of the Nematode orders were curated by manual inspection. The frequency of the SVs in each sample were converted based on their relative abundance and visualized using phyloseq [47]. The phylum-level compositions of the SVs (frequency >1% of the average) were shown as bar plots for each region (Fig 1B and S9 Fig). The order-level compositions of the nematode-derived SVs (frequency >1% of the average) were shown as bar plots for each region (Figs 1C and 8).

The resultant nematode-derived SVs in each region (i.e., regional nematode SVs) were named according to their region and SV number, e.g., SV 1 in region 1 was named as R1_SV_1. The SV numbers (e.g., 298 of R1_SV_298) were counted along with the abundance of sequence reads of SV, where a lower number represents the SV containing a larger number of reads. The relative abundance of each regional nematode SV was determined as the percentage of the reads of the SV in the total sequence reads of all regional nematode SVs. The taxonomic analysis of regional nematode SVs was performed two ways. First, the taxonomic ranks of the regional nematode SVs were determined based on the SILVA database [46]. Second, regional nematode SVs were assigned to an order, family, and genus in accordance with their closest species match via a BLASTN search of the National Center for Biotechnology Information (NCBI) website (https://www.ncbi.nlm.nih.gov) in October 2020. The resulting entries with the smallest e-values, which represent hits’ significance in Expect value, were only used for identification of the closest species to the queried nematode SVs (i.e., matches without genus data, such as environmental samples, were omitted even if they had the smallest e-values). A few questionable SVs found by BLASTN search were removed from the regional nematode SVs identified by the SILVA database, and the resultant regional nematode SVs and their taxonomic data were used for further phylogenetic analyses. Four phylogenetic trees of the regional nematode SVs were constructed using the corresponding regional sequences of *Halobiotus crispae* (phylum Tardigrada) as an outgroup. The nucleotide sequences were aligned, and phylogenetic trees were constructed using the BOOTSTRAP N-J TREE algorithm (bootstrap: 1000 replicates) with the ClustalX (version 2.1) package (http://www.clustal.org/clustal2/) [48] in the Genetyx-MAC software (version 19, Genetyx Co.). The resultant tree files were used to draw the cladograms using the Genetyx-Tree software (version 2.2.6, Genetyx Co.). Additionally, the ATGC software (version 6, Genetyx Co., Tokyo, Japan) was used to screen for identical or closest regional nematode SVs and the SSU gene-derived operational taxonomic units (rOTUs) found by our group [26, 28] to regional nematode SVs under with a 99% match and minimum matching number of 200 bp, followed by manual inspection.

Feeding types of the regional nematode SVs were assigned according to their corresponding genus as identified by a BLASTN search using the method described by Yeates et al. [6] and the Nematode Ecophysiological Parameter Search at the Nemaplex homepage of UC Davis, USA (http://nemaplex.ucdavis.edu/Ecology/EcophysiologyParms/EcoParameterMenu.html) in November 2020 [49]. SVs identified as belonging to animal parasites were classified according to parasitic nematodes [50–52]. The colonizer–persister (cp) values of nematode families were classified according to the research by Bongers [53, 54] and the Nematode Ecophysiological Parameter Search [49]. Maturity indices were calculated as the weighted mean of the individual cp-values, as described by Bonger (1990) [54]:

$$Maturity\;Index=\sum_{i=1}^{n}v(i)\times f(i)$$

where *v*(*i*) is the cp-value of family *i* and *f*(*i*) is the frequency of reads from the family compared to those from all families without reads derived from unassigned families (NA) in a sample (S2–S6 and S11 Tables).

Additionally, relative abundances and taxa (orders) of rOTUs identified in prior studies were integrated into the cladograms of the nematode-derived SVs in regions 1–4 to compare the taxonomic profiles with previous data and each sample was identified by the soil sample code and experimental ID such as H02 (i.e., the experiment of ID 02 using a field (code H)-derived soil sample) as indicated in S2 Fig.

## Results

### Sequence variants identified from the three nematode communities in four SSU gene regions

Sample DNAs were prepared from isolated nematodes from uncultivated field, copse and cultivated house garden (Fig 1A) and the PCR amplification of each SSU gene fragment was performed using four primer sets with tail sequences for adopting the Illumina MiSeq platform (Table 1). During agarose gel electrophoresis, the resultant PCR products with expected sizes were found in the reactions with the nematode DNA from the copse, house garden, and field soils, as detailed in S3A, S3B and S3C Fig, respectively, and successively subjected to massive DNA sequencing using MiSeq. The sequence reads were processed to extract the SVs in each region, and nematode-derived SVs were identified from the total SVs in each region based on the taxonomic ranks provided by the SILVA database as described in Materials and methods.

As detailed in Table 2, most SVs were detected in region 1 in the field and copse samples (146 and 279 SVs, respectively) and region 2 in the house garden sample (226 SVs), whereas the fewest SVs were obtained in region 3 in each sample. The copse sample had the most SVs in the SSU gene regions, except in region 3. Of the total SVs, 26–46, 38–88, 23–34, and 27–38 were nematode-derived SVs in regions 1–4, respectively. The most nematode-derived SVs in each sample were 39 in region 1 (field), and 88 (copse) and 44 (house garden) in region 2. Fewer nematode-derived SVs were detected in region 3; however, the fractions of nematode-derived SVs in the total SVs were significantly higher (37.1%–48.5%) than those in the other three regions (14.8%–35.8%). Among each sample, the most nematode-derived SVs were obtained in the copse sample in regions 1 (46 SVs) and 2 (88 SVs) and the field sample in regions 3 (34 SVs) and 4 (38 SVs).

**Table 2:** Numbers of SILVA-based total and nematode-derived SVs in each SSU region.

	Sampling site	Region 1	Region 2	Region 3	Region 4
Total SVs	Field	146	120	81	122
	Copse	279	246	66	172
	House garden	176	226	62	147
Nematode-derived SVs^a^	Field	39 (26.7%^b^)	38 (31.7%)	34 (42.0%)	38 (31.1%)
	Copse	46 (16.5%)	88 (35.8%)	32 (48.5%)	34 (19.8%)
	House garden	26 (14.8%)	44 (19.5%)	23 (37.1%)	27 (18.4%)

^a^ Numbers of nematode-derived SVs are based on the taxonomic ranks in the SILVA database [46].

^b^ Percentage of nematode-derived SVs in the total number of SVs.

Next, the taxonomic ranks of all SVs were determined in each region based on the SILVA database [46] (i.e., R1–R4 in Fig 1B). In the uncultivated field sample, the majority of sequence reads (60%–85%) were derived from the phylum Nematodes; the second-most abundant phylum was Streptophyta (plants). In both the copse and house garden samples, however, nematode-derived reads were less than 50% of the total reads. Only 10%–30% of the SVs in the house garden sample were nematode-derived; reads derived from phylum Ascomycota and Basidiomycota (i.e., fungi), Arthropoda (i.e., insects), and Tardigrada were more dominant. Although the phylum compositions of reads were comparable in regions 1, 2, and 4, the relative abundance of nematode-derived sequences was the largest in region 3. The fungi-derived fraction in each sample was markedly lower in region 3 than the other regions (R3 columns in Fig 1B).

### Taxonomic analyses of nematode-derived SVs from three samples in four regions

Amplicon sequencing of the three samples provided 92, 145, 69, and 76 nematode-derived SVs in regions 1–4, respectively (S2 Table). The resultant nematode-derived SVs in each region were named as regional nematode SVs according to their region and SV number, as detailed in Materials and methods. The regional nematode SVs were further classified to orders based on the SILVA taxonomic ranks, as shown in Fig 1C, where the nematode orders are indicated by colors and independent SVs are indicated by boxes in the histograms alongside their abundances. Comparable relative abundances of nematode SVs were obtained at the order level in both copse and house garden samples in four regions, although the relative abundance of SVs assigned to order Rhabditida was significantly higher (>50%) in region 2 in the field sample than in other regions and samples. Many Rhabditida-derived SVs were detected in each sample, as indicated by the blue columns in the histograms shown in Fig 1C. Overall, the most abundant orders of SVs in each nematode sample were, in order: Dorylaimida, Mononchida, and Rhabditida in the field sample, Dorylaimida and Rhabditida in the copse sample, and Rhabditida in the house garden sample, indicating different nematode communities in these soils.

**Fig 2:**
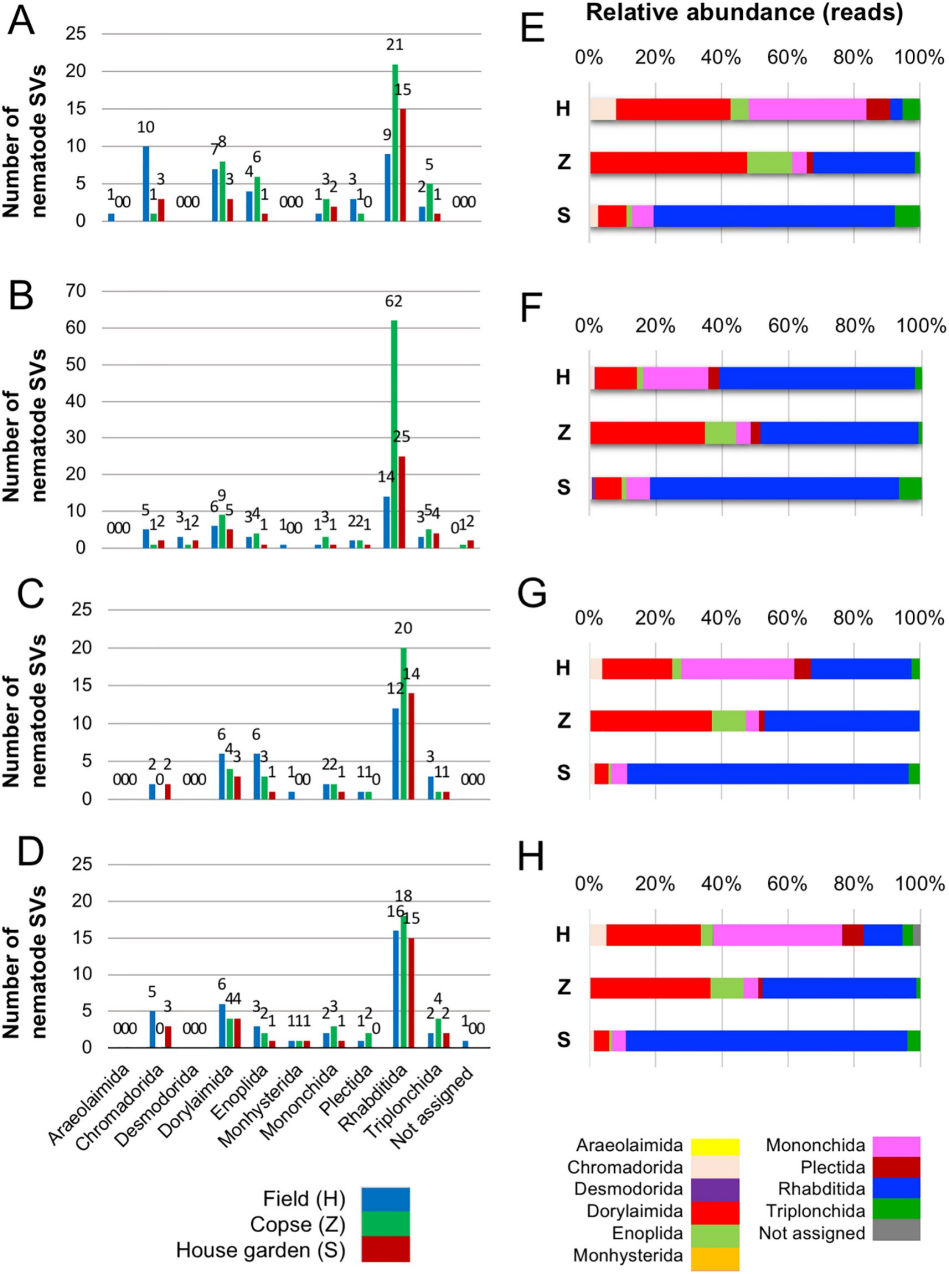
Numbers of regional nematode SVs and relative abundance of nematode-derived sequence reads at the order level in each region. Numbers of nematode SVs assigned to 10 orders are indicated at the bottom of (D) and unassigned SVs identified from the field (blue), copse (green), and house garden (red) samples are indicated in the histograms in regions (A) 1, (B) 2, (C) 3, and (D) 4. The number on top of the histogram corresponds to the numbers of SVs. The percentage of the abundance of sequence reads corresponding to nematode order obtained from regions (E) 1, (F) 2, (G) 3, and (H) 4 are indicated in the field (H), copse (Z), and house garden (S) samples in the horizontal histogram by color. The colors corresponding to orders are indicated at the bottom of (H). Orders of regional nematode SVs were assigned based on the BLASTN search.

The taxonomic groups of nematodes living in each of the three soils were then investigated via sequence homology-based phylogenetic analysis of the nematode SVs. Before phylogenetic analysis, the taxa of the regional nematode SVs identified by the SILVA-based classification were further evaluated by a BLASTN search. The filtration by BLASTN search identified four, one, two and zero of non-nematode-derived SVs and removed from regional nematode-derived SVs of regions 1–4, respectively, and the resultant 88, 144, 67 and 76 regional nematode SVs were used for further analysis, respectively (see S2 Table). The resultant BLASTN-based taxonomic information (order, family, and genus) of the regional nematode SVs are detailed along with their SILVA-based taxonomic ranks and feeding types in S3–S6 Tables. Each SV was classified according to feeding type (i.e., bacteria feeder, fungus feeder, plant feeder, eukaryote feeder, omnivore, predator, or animal parasite) based on that of the genus with the highest sequence homology to the SV. A few SVs with multiple feeding types were classified as “not assigned” (NA). The total and relative abundances of the regional nematode SVs from the three samples are summarized by order in Fig 2. Nematode SVs derived from 10 orders were found, including Araeolaimida, Chromadorida, Desmodorida, Dorylaimida, Enoplida, Monhysterida, Mononchida, Plectida, Rhabditida, and Triplonchida.

The Rhabditida-derived SVs were most abundant across the samples in the four SSU gene regions studied except for 10 SVs derived from the order Chromadorida in the field sample in region 1 (Fig 2A–2D). The numbers of SVs derived from the orders Dorylaimida (18, 20, 13, 14 SVs in regions 1–4 from three samples), Enoplida (11, 8, 10, and 6 SVs), and Triplonchida (8, 12, 5, and 8 SVs) were relatively abundant, followed by the orders Chromadorida (14, 8, 4, and 8 SVs)-, Mononchida (6, 5, 5, and 6 SVs)-, and Plectida (4, 5, 2, and 3 SVs)-derived SVs. The SVs derived from the orders Araeolaimida (1 SV in region 1), Desmodorida (6 SVs in region 2), and Monhysterida (1 SV in regions 2 and 3, 3 SVs in region 4) were all found only in very minor fractions. The number of SVs in region 2 from the copse sample was markedly large; most of them were derived from the Rhabditida order. Twice as many Chromadorida-derived SVs were present in region 1 than elsewhere in the field sample (see the blue histograms in Fig 2A). Otherwise, the SV numbers in each order were largely comparable across the three samples and four regions. Further, the relative abundances of reads in each order were comparable in the copse and house garden samples, whereas those in the field sample were different among the regions (Fig 2E–2H). The relative abundance of reads derived from the order Rhabditida was significantly high (58.8%) in region 2 compared with others (30.5% in region 3, 11.7% in region 4, and 3.8% in region 1), causing the regional difference of relative read abundances in orders in the field sample. The compositions of the relative read abundances were similar in both regions 1 and 4 (Fig 2E and 2H). The most abundant orders were Dorylaimida and Mononchida in the field sample, Dorylaimida and Rhabditida in the copse sample, and Rhabditida in the house garden sample.

We identified the nematode-derived SVs with less than 99% sequence identity to the publicly available SSU sequences in each region as novel SVs (S7 Table). Thirty-six (40.9% in total nematode-derived SVs found in region 1) and sixty-five (45.1%) out of 88 and 144 SVs were detected in regions 1 and 2, respectively. However, 12 (17.9%) and 15 (19.7%) out of 67 and 76 SVs were identified in regions 3 and 4, respectively. The majority of these SVs in regions 1 (17 of 36 SVs), 2 (44 of 65 SVs), and 4 (10 of 15 SVs) were derived from the order Rhabditida, but 7 of the 12 SVs in region 3 were from the order Enoplida. Furthermore, 17, 7, and 8 of the 44 Rhabditida-derived SVs in region 2 were highly similar to the genera *Pratylenchus*, *Travassosinema*, and *Filenchus*, respectively. All novel Chromadorida-derived SVs in regions 1 and 2 are likely to belong to the genus *Achromadora*, and 7 novel SVs in region 3 were derived from two genera (*Alaimus* and *Trischistoma*) in Enoplida.

Additionally, we investigated possible polymorphic SV pairs sharing fewer than two different nucleotides (S8 Table) as we found several polymorphic alleles in four SSU regions in previous amplicon sequencing of the isolated nematodes [28]. However, a significant difference of the resultant SVs was not found among the four regions.

### Cladograms, feeding types, and maturity indices for the three nematode communities in the four regions

**Fig 3:**
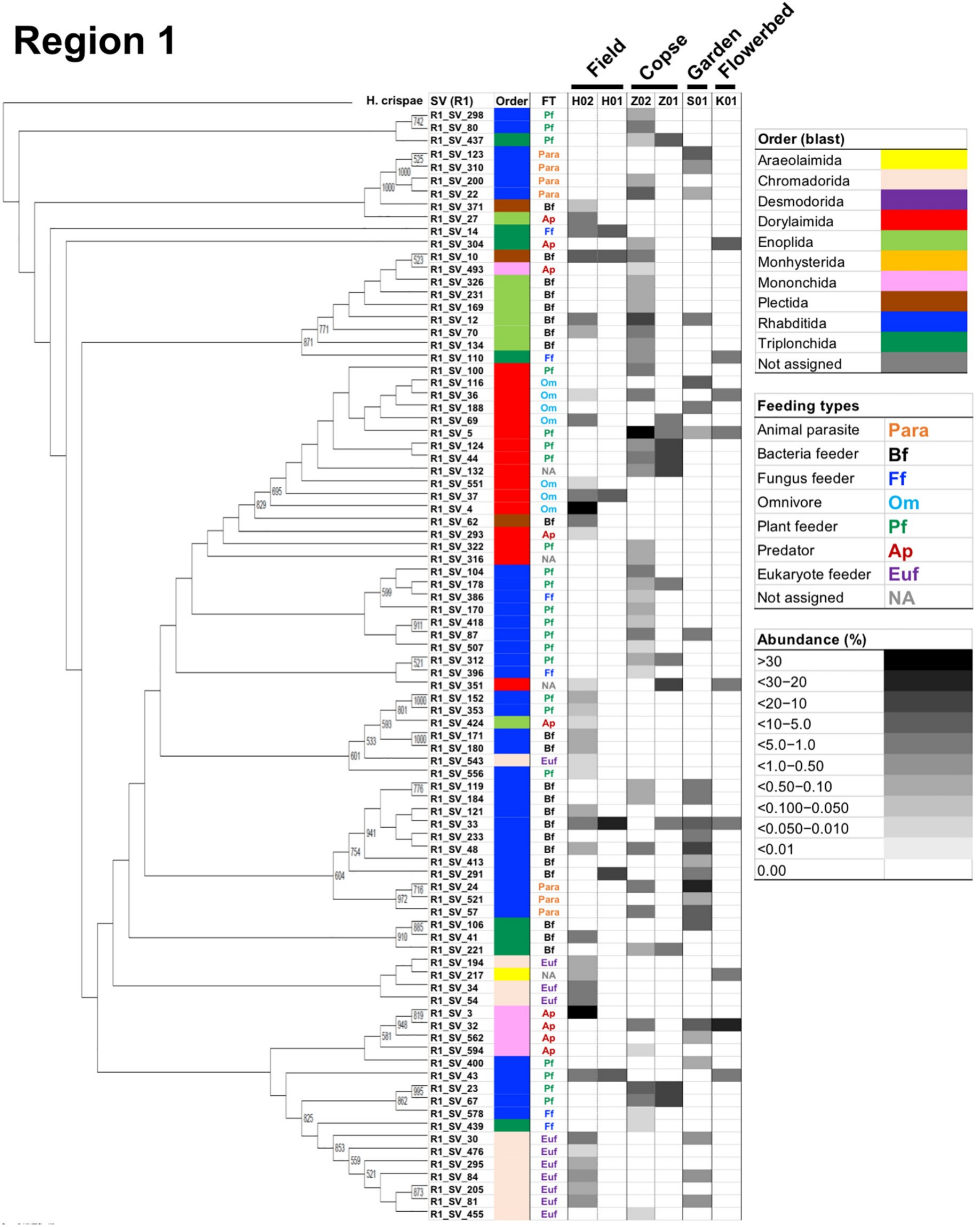
A cladogram of nematode SVs in region 1 with the corresponding orders, feeding types, and relative read abundances. The cladogram was prepared using nematode SVs from region 1 and the corresponding SSU gene sequence of *H*. *crispae* as the outgroup as described in the Materials and methods section. Bootstrap numbers of over 500 per 1000 are indicated at the nodes of the cladogram. The name of the nematode-derived SV in region 1 was indicated as R1_SV_number at the branch end of the cladogram. Orders and feeding types (FT) of the nematode SVs are indicated in the corresponding columns at the right of the horizontally compressed cladogram by colored bars and abbreviations, as shown in the legend boxes. Relative read abundance of nematode SVs in the samples from the field (H02), copse (Z02), and house garden (S01) soils are indicated by density bars. Orders, feeding types, and relative abundance (i.e., percentage of the number of nematodes in the SSU gene-derived operational taxonomic units (rOTU)) of the rOTUs that were previously identified also indicated, where H01 represents the rOTUs from the soybean-cultivated field and K01 represents those from the unmanaged flowerbed soils sampled in 2010 [26] and Z01 represents those from the copse soils sampled in 2018 [28] (see S2 Fig).

**Fig 4:**
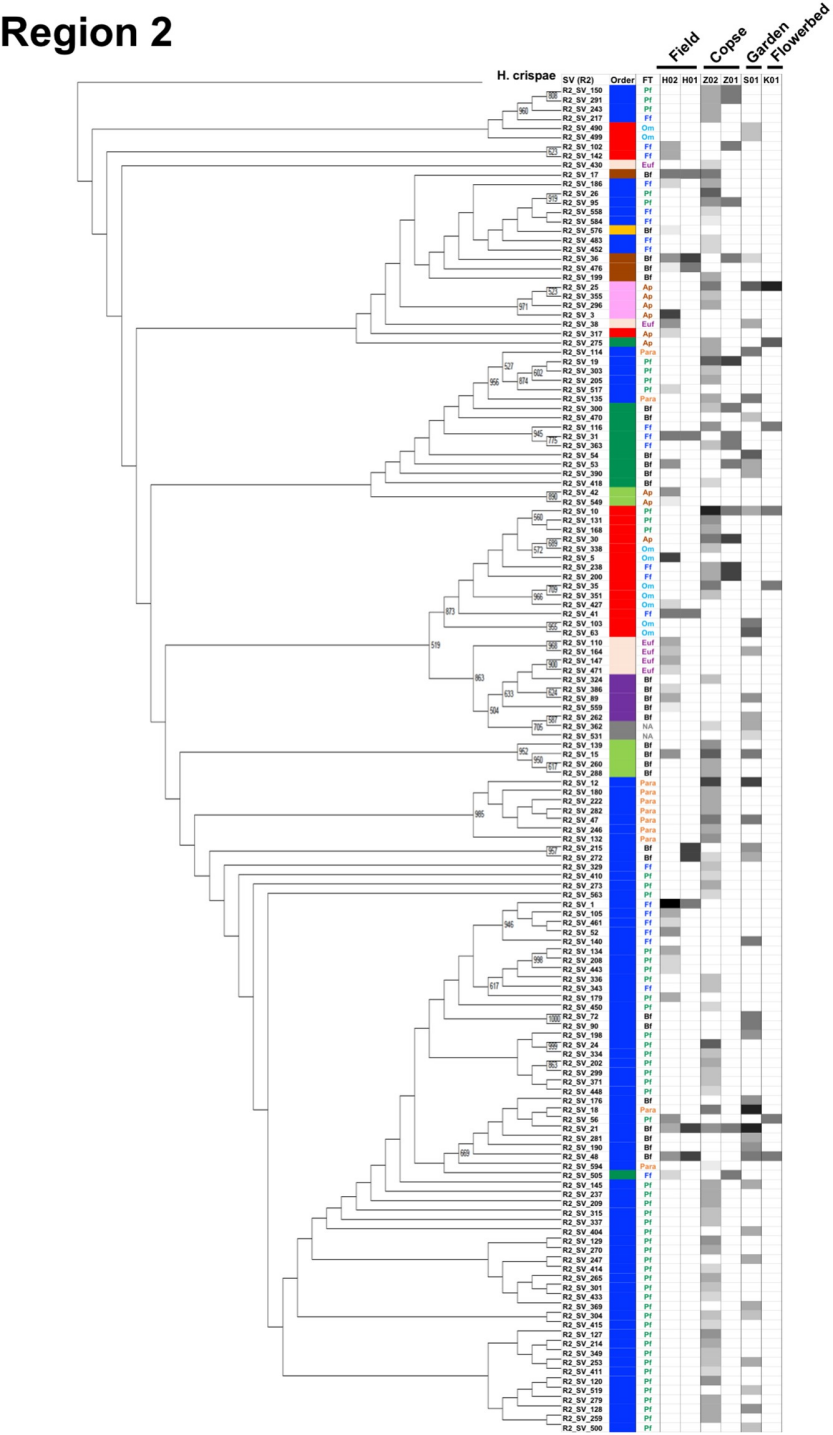
A cladogram of nematode SVs in region 2 with the corresponding orders, feeding types, and relative read abundances. The cladogram was prepared using nematode SVs of region 2 as described in the Materials and methods section. Orders, feeding types, and relative read abundances of the nematode SVs are indicated in the corresponding columns at the right of the horizontally compressed cladogram by colored bars, colored abbreviations, and density bars, respectively, as detailed in the caption of Fig 3 above.

**Fig 5:**
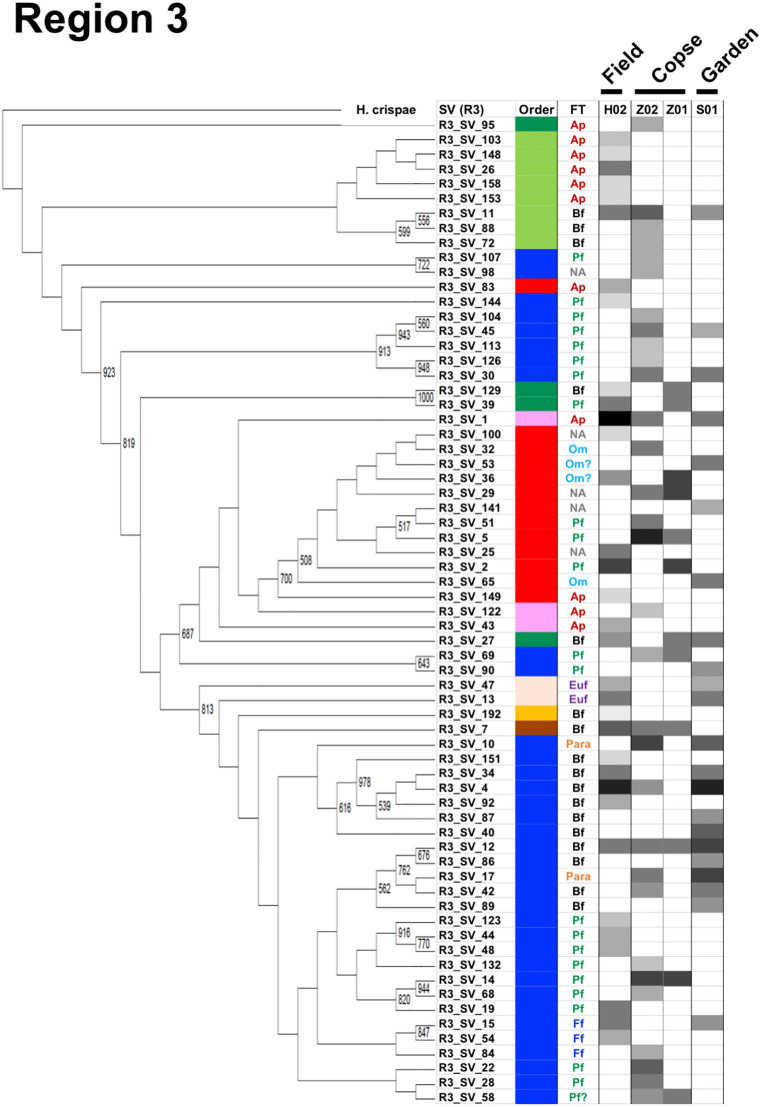
A cladogram of nematode SVs in region 3 with the corresponding orders, feeding types, and relative read abundances. The cladogram was prepared using nematode SVs of region 3 as described in the Materials and methods section. Orders, feeding types, and relative read abundances of the nematode SVs are indicated in the corresponding columns at the right of the horizontally compressed cladogram by colored bars, colored abbreviations, and density bars, respectively, as detailed in the caption of Fig 3 above.

**Fig 6:**
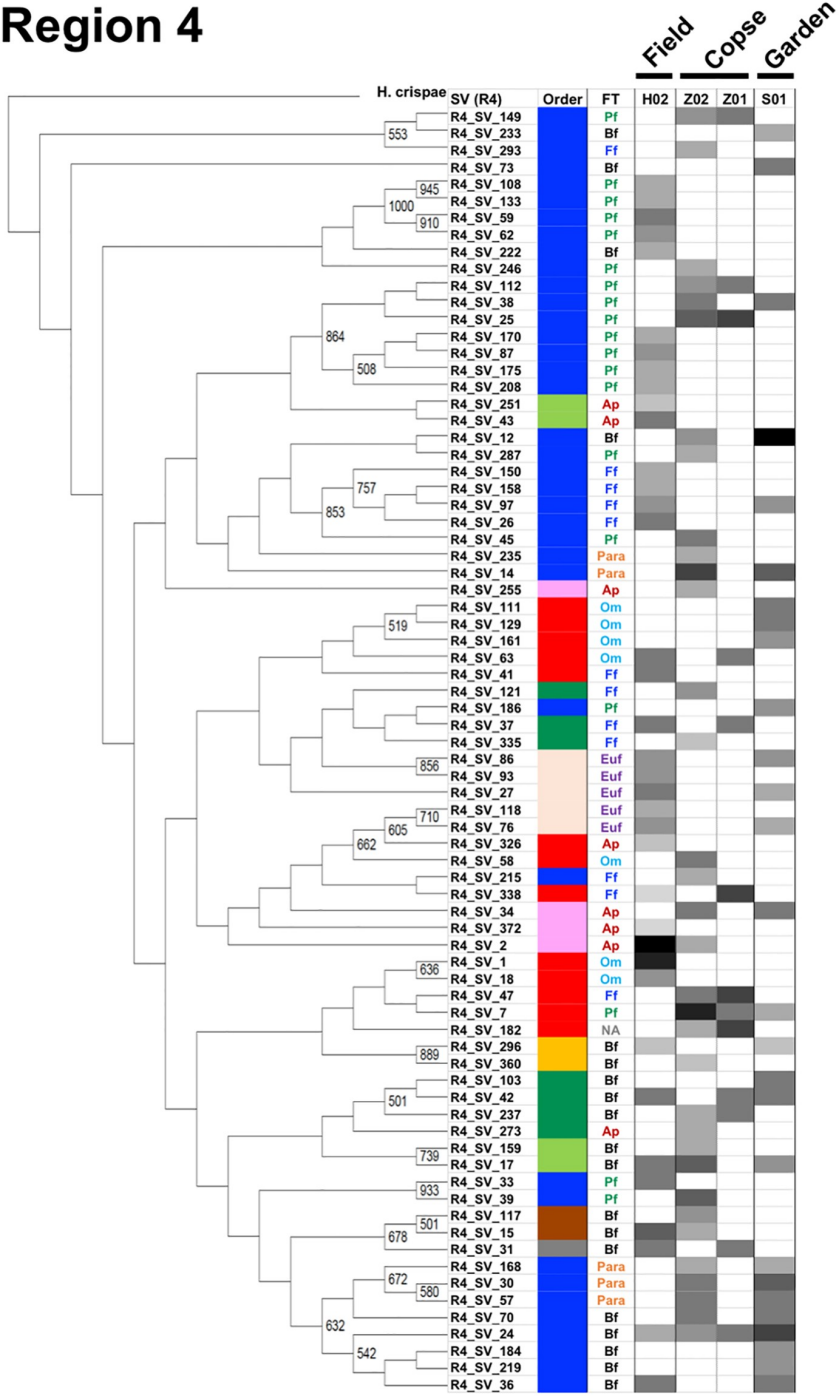
A cladogram of nematode SVs in region 4 with the corresponding orders, feeding types, and relative read abundances. The cladogram was prepared using nematode SVs of region 4 as described in the Materials and methods section. Orders, feeding types, and relative read abundances of the nematode SVs are indicated in the corresponding columns at the right of the horizontally compressed cladogram by colored bars, colored abbreviations, and density bars, respectively, as detailed in the caption of Fig 3 above.

A cladogram was built for each region using nematode SVs and the corresponding SSU gene sequence of *H*. *crispae* (phylum Tardigrada) as an outgroup (Figs 3–6), where the relative abundance of each SV in the samples are indicated as H02, Z02, and S01, indicating the field, copse, and house garden samples, respectively. A few regional nematode SVs were only shared among the field (H02), copse (Z02), and house garden (S01) samples: 2 out of 88 nematode SVs in region 1 (R1_SV_12 and R1_SV_48 in Fig 3), 2 out of 144 SVs in region 2 (R2_SV_15 and R2_SV_21 in Fig 4), 4 out of 67 SVs in region 3 (R3_SV_1, R3_SV_4, R3_SV_11 and R3_SV_12 in Fig 5), and 2 out of 76 SVs in region 4 (R4_SV_17 and R4_SV_24 in Fig 6). This clearly indicates the uniqueness of the taxonomic compositions in each nematode community. The resultant regional nematode SVs and their corresponding rOTUs were compared with prior data from copse soils in mid-summer 2018 [26, 28] and soybean-cultivated field and unmanaged flowerbed soils in winter 2010–2011 [26, 28] (S2 Fig). Seventeen of the eighteen previously identified rOTUs from the copse-derived nematodes [28] were identified in the SSU gene regions (S9 Table). Using a 900-bp DNA barcode encompassing regions 1 and 2, Morise et al. [26] identified 13 and 15 rOTUs derived from nematodes in the cultivated field and flowerbed soils, respectively. Here, 8 of the 13 (62%) and 9 of the 15 (60%) rOTUs were identical to the SVs of regions 1 and/or 2 in this study (S10 Table). Despite the presence of undetected rOTUs in this study, several nematode SVs from the field and copse samples corresponded with previously determined rOTUs (S9 and S10 Tables). This indicates taxonomic similarities in the two comparable fields (columns H01 and H02 in Figs 3 and 4) and copse samples (columns Z01 and Z02 in Figs 3–6).

The characteristic profiles of read abundances and feeding habitats of nematodes in 10 orders were found in the three samples studied (Figs 3–6). The order with the largest number of nematode SVs was Rhabditida except in region 1 of the field sample (Fig 2A–2D); the SVs with the largest number of reads in the house garden sample were also derived from the Rhabditida in regions 1–4 (R1_SV_24, R2_SV_21, R3_SV_4, and R4_SV_12) (Figs 3–6 and S2 Table). The Rhabditida species includes four feeding groups (bacteria feeder, fungus feeder, plant feeder, and animal parasite). A large number of Rhabditida-derived SVs with a plant-feeding habitat were abundant in the copse sample and were detected in region 2, unlike in other regions (Fig 4). Bacteria, plant, and fungus feeders derived from the Rhabditida were most prevalent in the field sample; bacteria feeders and animal parasites were dominantly found in the house garden sample (Figs 3–6). Interestingly, the Rhabditida-derived animal parasites were abundant in the house garden sample and present in the copse sample but were not found in the field sample. The SVs derived from orders Dorylaimida and Mononchida were also abundant and found in each nematode community. The Mononchida-derived SVs (R1_SV_3, R3_SV_1, and R4_SV_2) from the field sample occupied the most abundant fractions of reads in regions 1, 3, and 4 (Figs 3–6 and S2 Table). The members of this order belong to predators and were found in all three samples. The Dorylaimida-derived SVs (R1_SV_5, R2_SV_10, R3_SV_5, and R4_SV_7) from the copse sample contained the largest numbers of reads in regions 1–4. Nematodes in this order exhibit several feeding types; the omnivores were abundant in both the field and house garden soils despite their different SVs, whereas plant feeders dominated in the copse soil in addition to hyphal feeders. The members of the order Triplonchida also exhibit various feeding types (bacteria, fungus, plant feeders, and predators). The bacteria feeders were commonly found in all three communities but dominantly detected in the house garden sample. Fungus feeders dominated in the copse sample, and bacteria and fungus feeders were found in the field sample (Figs 3–6). The members of the order Enoplida are bacteria feeders and predators; they were dominantly found in the copse and field soils, respectively. Although a single SV derived from the genus Alaimus of order Enoplida was found in all regions (as R1_SV_12, R2_SV_15, R3_SV_11, and R4_SV_17), no other SVs of the Enoplida order was detected in the house garden sample (Figs 3–6 and S3–S6 Tables). Similar compositions and abundances of the Chromadorida-derived SVs were found in the field and house garden samples in regions 1, 3, and 4 (Figs 3, 5 and 6), but only two were present in the copse sample at low abundances (R1_SV_455 and R2_SV_430) (Figs 3 and 4). The SVs derived from the orders Desmodorida, Monhysterida, and Plectida occupied minor fractions. The Desmodorida-derived SVs were only detected in region 2 (Fig 4) and only one or two SVs from the Monhysterida were found in regions 2–4 (Figs 4–6). The Plectida species are bacteria feeders and were detected in both the field and copse samples, but poorly found in the garden sample (Figs 3–6).

Forty nematode families across four SSU gene regions were identified (see S11 Table); including 26, 31, 26, and 29 families in regions 1–4, respectively. Of these 40 families, 19 were commonly detected in all four regions; 12 families were only found in a single region. A larger number of unassigned SVs (10 SVs) were found in region 3 than in other regions (4–6 SVs); this may have been caused by increased ambiguous BLAST-hit entries due to the shorter nucleotide sequence of region 3 (290 bp vs approximately 350 bp in regions 1 and 2 and 320 bp in region 4). The number of nematode SVs in each family is shown along with the corresponding colonizer–persister (cp)-values in regions 1–4 in S4A–S4D Fig. The cp-values (1–5) have been used as indicators for nematode’s life strategy characteristics [23, 53, 55]: nematodes with low cp-values have a short generation time and produce many small eggs, resulting in an explosive population growth under food-rich conditions, whereas those with high cp-values have a long life span and low production rate, and are thus highly sensitive to environmental disturbances, such as pollution. SVs derived from the families of lower cp-groups were preferentially found in the house garden and field samples, whereas SVs from several families with higher cp-values were detected in the copse and field samples (S4 Fig).

The relative abundance of reads from the samples in each family was then analyzed and is summarized by cp-group in S5–S8 Figs. In the copse sample, the reads derived from the family Belondiridae (cp-value of 5 and plant feeder) were consistently the most abundant. In the house garden sample, the most abundant reads were derived from the Cephalobidae (cp-value of 2 and bacteria feeder) and Ungellidae (cp-value unknown, animal parasite) families in regions 1 and 2 and the Rhabditidae (cp-value of 1) and Cephalobidae families in regions 3 and 4 (S5C–S8C Figs), suggesting dominant propagations of nematodes of low cp-groups in the garden soils. In the field sample, the most abundant families were the Mylonchulidae (predator) and Qudsianematidae (omnivore) families with a cp-value of 4 in region 1, and the Aphelenchidae (cp-value of 2 and fungus feeder) and Mylonchulidae families in region 2 (S5A and S6A Figs). Whereas unassigned SVs (NA) were the most abundant in regions 3 and 4, nearly all of these were derived from a single regional SV (R3_SV_1 and R4_SV_2) that was most similar to nematodes of the family Mylonchulidae or Mononchidae (predator) in the order Mononchida (see S5 and S6 Tables). The family Rhabditidae and Dorylaimidae (cp-value of 4) were the second-most abundant in regions 3 and 4 (S7A and S8A Figs), respectively, suggesting the presence of nematode groups with both low and high cp-values in the field soil. We further determined the maturity indices, which can be used as ecological measures of environmental disturbance [54], for the three sampling sites based on the cp-values and the frequency of each family in the four SSU regions (Table 3). The highest and lowest values for the maturity indices were commonly obtained in the four regions from the copse and house garden samples, respectively. Since a higher value of the maturity index indicates a stable and undisturbed soil environment and *vice versa*, these results were consistent with the distributions of the nematode families in the three sample soils, as shown above. Furthermore, they indicate distinct environments in undisturbed copse soils and house garden soils disturbed by tillage and fertilizers.

**Table 3:** Maturity indices for three sampling sites in four SSU regions.

Sampling site	Region 1	Region 2	Region 3	Region 4
Field	3.63	2.71	2.75	3.26
Copse	4.23	3.86	3.97	3.89
House garden	2.73	2.58	1.58	1.78

Maturity indices were calculated as described in the Materials and methods section, based on the method described by Bongers [54].

**Fig 7:**
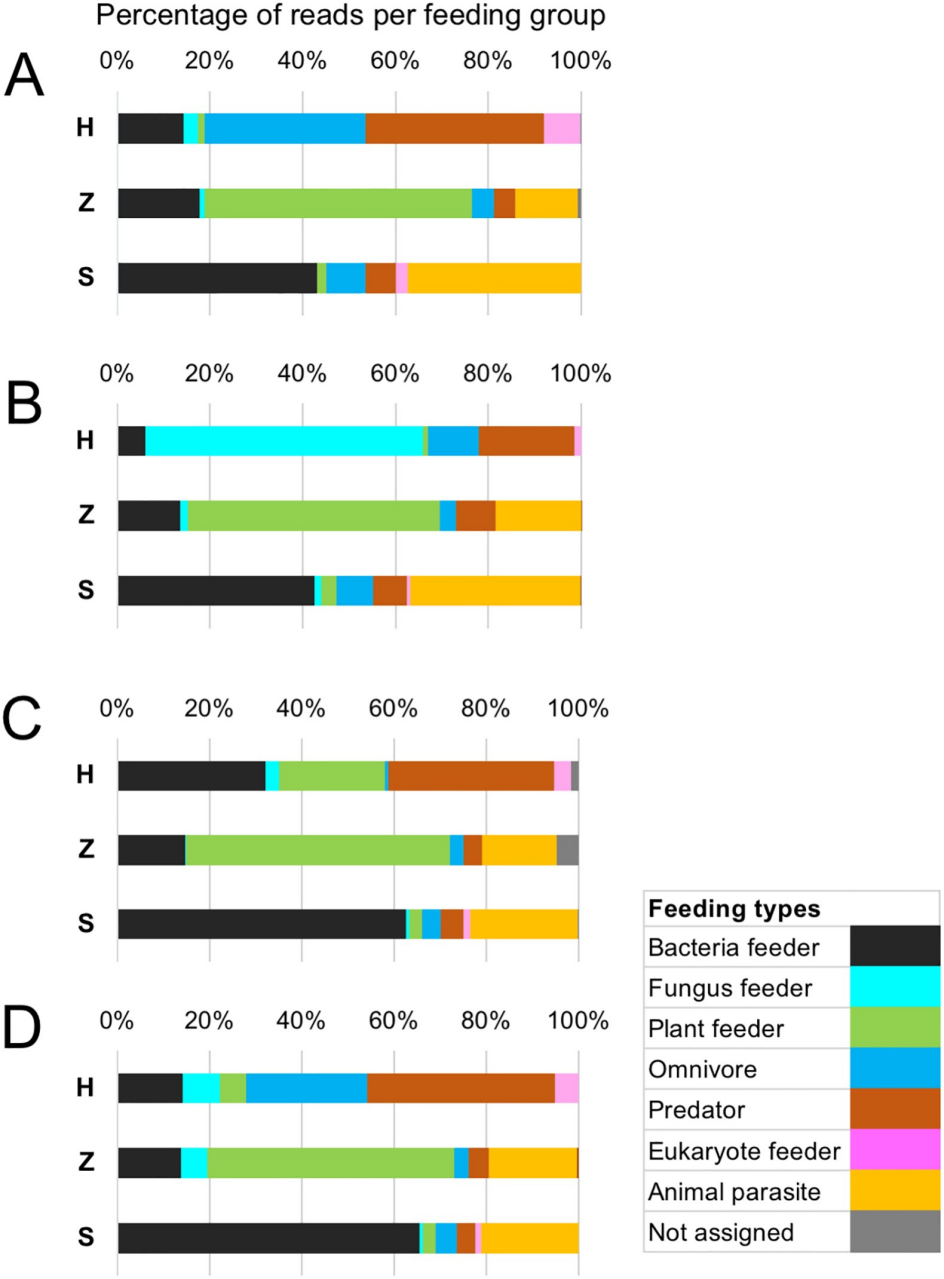
Relative abundances of sequence reads by feeding group across all samples. Nematode SVs identified from the field (H), copse (Z), and house garden (S) samples were assigned to one of seven feeding types (bacteria feeder, fungus feeder, plant feeder, omnivore, predator, eukaryote feeder, and animal parasite) as described in the Materials and methods section. Percentages of the sequence reads of nematode SVs in feeding groups are shown by colored fractions of horizontal histograms in regions (A) 1, (B) 2, (C) 3, and (D) 4, where the colors corresponding to each feeding type are shown in the legend box. The fractions of the SVs with multiple feeding types are classified as “not assigned” and indicated in gray.

To investigate which feeding types were most present in each nematode community, the fractions of the nematode-derived reads in regions 1–4 are summarized by feeding group in Fig 7A–7D. Despite minor differences, similar feeding type compositions were found among the four regions in the copse and house garden nematode communities (columns Z and S, respectively), i.e., plant feeders and bacteria feeders as the largest fraction, respectively. Interestingly, animal parasites also occupied significant fractions (approximately 14%–37%) in both of these nematode communities. Unlike the copse and garden samples, however, the relative abundances of reads in feeding types were varied among the regions in the field sample (column H): predator ≧ omnivore > bacteria feeder in regions 1 and 4, fungus feeder > predator in region 2, and predator = bacteria feeder > plant feeder in region 3. Thus, the read abundance profiles in feeding types in the field sample were similar in regions 1 and 4, but not in regions 2 and 3. The most dominant feeding types of each region included: predator and omnivore in region 1 (R1_SV_3 at 35.2% and R1_SV_4 at 30.6%, respectively); fungus feeder and predator in region 2 (R2_SV_1 at 55.8% and R2_SV_3 at 19.7%, respectively); predator, bacteria feeder, and plant feeder in region 3 (R3_SV_1 at 33.9%, R3_SV_4 at 22.3%, and R3_SV_2 at 18.8%, respectively); and predator and omnivore in region 4 (R4_SV_2 at 39.0% and R4_SV_1 at 24.1%, respectively) (S2–S6 Tables).

### Comparative analysis of amplicon sequences from one-step PCR with tailed primers and two-step PCR with tailless and tailed primers

PCR primers with tail sequences have commonly been used for amplification of SSU gene fragments for adopting to the Illumina MiSeq platform, however it has not been investigated the influence of the tail sequences in amplicon sequencing. To clarify this point, PCR was performed using nematode DNAs isolated from the field soil as a template with four primer sets including tailed or tailless primers (see Table 1). Amplicons from 35 PCR cycles with tailed primers (one-step PCR) and 27 PCR cycles with tailless primers followed by 8 cycles with the corresponding tailed primers (two-step PCR) were prepared and their resulting nucleotide sequences were analyzed; the results are summarized in Table 4. In region 1, the one- and two-step analyses of amplicons produced the same number of SVs and nematode-derived SVs, and similar results were obtained in regions 2 and 4. However, the total number of SVs varied significantly in region 3 when using the one- and two-step processes.

**Table 4:** Total and nematode-derived SVs from the high-throughput sequencing of amplicons prepared from one-step PCR with tailed primers and two-step PCR with tailless and tailed primers.

	Sample	Region 1	Region 2	Region 3	Region 4
Total SVs	One-step PCR^b^	146	120	81	122
	Two-step PCR^c^	146	140	49	128
Nematode-derived SVs^a^	One-step PCR	39 (26.7%)	38 (31.7%)	34 (42.0%)	38 (31.1%)
	Two-step PCR	39 (26.7%)	39 (27.9%)	30 (61.2%)	36 (28.1%)

^a^ Nematode-derived SVs were selected based on the taxonomic ranks in the SILVA database.

^b^ Amplicons from the one-step PCR with tailed primers (35 cycles) as used for amplicon sequencing of the three nematode samples.

^c^ Amplicons from the two-step PCR with tailless primers (27 cycles) followed by tailed primers (8 cycles).

**Fig 8:**
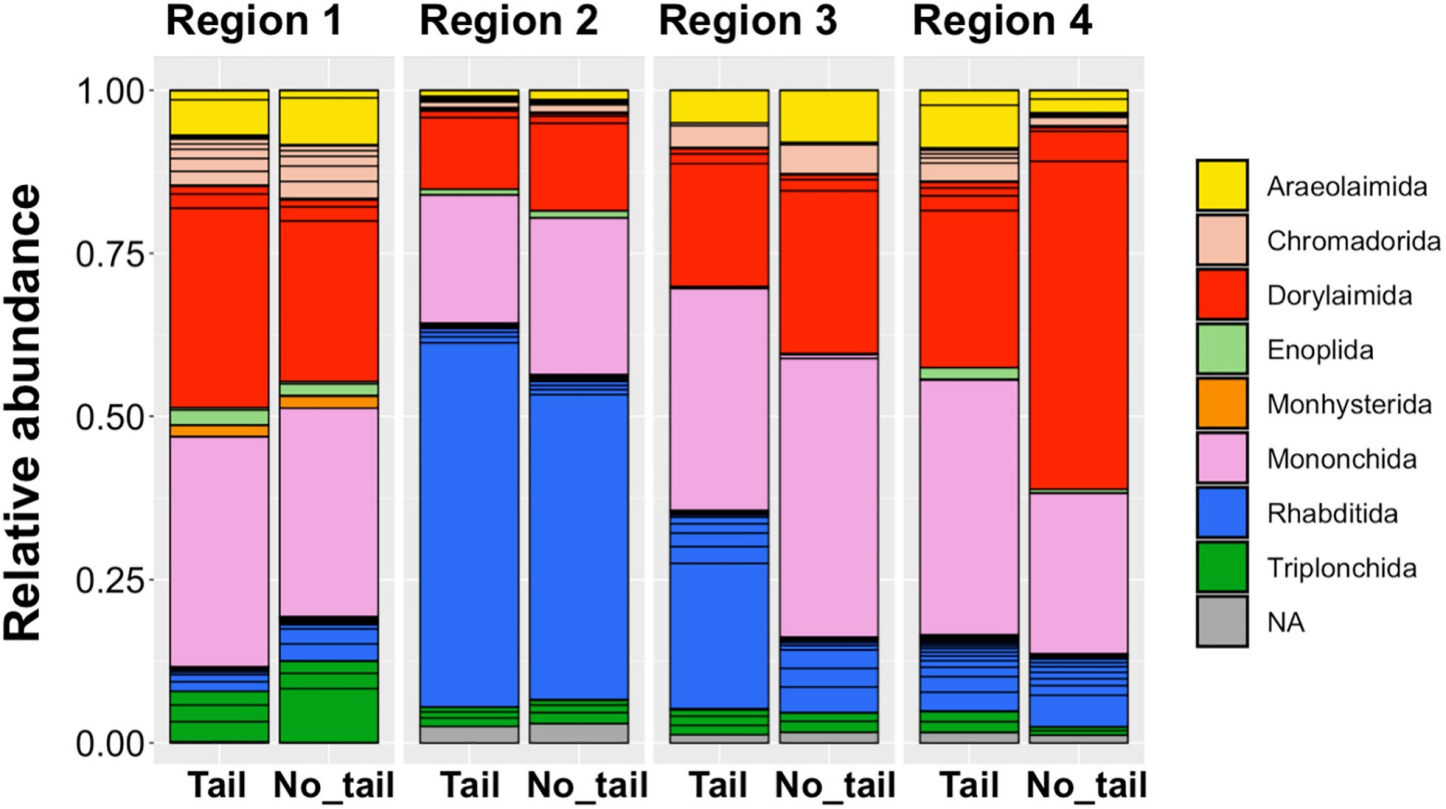
Relative read abundance of nematode-derived SVs in each order from massive sequencing of amplicons prepared by one- and two-step PCRs. The amplicons of the four SSU gene regions indicated on top of the histograms were prepared via a one-step PCR with tailed primers (Tail) and a two-step PCR using tailless and tailed primers (No_tail) with the field sample DNA as a template. Nematode-derived SVs were identified from the SVs as described in the Materials and methods sections and their orders were determined based on the SILVA database. The boxes in each order’s column indicate the SVs and their relative abundance, whereas the colors corresponding to order are shown in the legend. NA: Not assigned.

The resulting phylum determinations and read abundances of the resultant SVs, summarized in S9 Fig, demonstrated that the major fractions of sequence reads were nematode-derived sequences in each region and comparable results were obtained from the analyses of both amplicons. The orders and read abundances of the regional nematode-derived SVs were also investigated; the resulting histograms from the analyses of the four sets of amplicons by one- and two-step PCR are shown in Fig 8. Similar results were obtained, except for minor differences in the abundance of the order Rhabditida in region 3 and the order Dorylaimida in region 4. The taxa and relative abundances of regional nematode SVs detected by both amplicon sequencing were then investigated; the results are shown in S10 Fig. Most of the SVs, including the major SVs, were shared by both amplicon data. The undetected SVs in the amplicon of regions 1–4 by one-step PCR were 8, 11, 7 and 7; 9, 11, 12, and 10 SVs in each region were undetected by the two-step PCR. Despite these minor differences, the composition and relative abundance of the regional nematode SVs from both amplicon sequencing methods were confirmed to be relatively comparable in each region.

## Discussion

### Barcoding of nematode communities by high-throughput sequencing-based amplicon sequencing of four SSU gene regions

Four SSU gene regions (i.e., regions 1–4) were investigated as suitable target regions for Illumina MiSeq-assisted DNA barcoding of nematode communities isolated from three different soils. Approximately nine variable regions of eukaryotic SSU genes have been suggested as potential DNA barcodes by Hugerth et al. [38] and Hadziavdic et al. [37]; several metabarcoding studies of isolated terrestrial nematode communities have been reported. In many of these, the V7–V8 region (region 4 in this study) of the SSU gene amplified by the primer set including NF1 and 18Sr2b has been preferentially used as a DNA barcode [20, 22, 30–33, 36, 56] (S1 Fig). The V1–V2 (here, region 1) [36], V4–V5 (here, region 2) [21, 32, 57, 58], and V9 [58] regions have also been used for DNA barcoding of nematodes (S1 Fig). The regions 1 (V1–V2), 2 (V4), 3 (V5–V7), and 4 (V7–V8) in this study were analyzed for their capabilities in the DNA barcoding of individual nematodes isolated from the copse soil, as published by Kenmotsu et al. [28]; region 4 was indicated as a suitable barcode of the SSU gene. In this work, a larger number of nematode-derived SVs was obtained in region 2 than in the other three regions in the copse and house garden samples, but not in the field sample (see Table 2). As polymorphic alleles of nematode SSU genes [59] may have accounted for the increased SVs in region 2, possible polymorphic SV pairs sharing less than two different nucleotides were investigated, as detailed in S8 Table. However, a significant difference of resultant SVs was not found among the four regions, thus discrediting this hypothesis. Although consistent reads were detected in both copse and garden samples across the regions, Rhabditida-derived SVs were highly abundant in the field sample in region 2 (Fig 2F), but not in regions 1 and 4 (Fig 2E and 2H). These inconsistent results from amplicon sequencing of region 2 have not yet been accounted for. Some researchers have performed metabarcoding of soil nematodes [21, 32, 57], and the V4 region (encompasses in region 2) has been suggested to be useful for taxonomic analysis of eukaryotes [35]. Waeyenberge et al. [32] compared two primer sets (EcoF/EcoR and optimized NemF/18Sr2b) by the high-throughput amplicon sequencing of artificial nematode communities and reported less taxonomic coverage and increased non-nematode-derived reads in amplicons by the former primers, whose target region almost overlaps with region 2, than those by the latter primer set for amplification of region 4 (see S1 Fig). Taken together with different profiles of taxa and feeding types in region 2 as discussed in the next section, future efforts should focus on investigating region 2 for DNA barcoding by repeated experiments. The lowest numbers of SVs were found in region 3, but this region had the highest relative abundance of nematode-derived SVs (37%–49%) (see Table 2). Further, a significantly lower fraction of SVs was fungi-derived (see Fig 1B). These observations are consistent with those of prior DNA barcoding results using individual nematodes [28] and indicated that the primers for region 3 could generate amplicons in a nematode-specific manner. However, in addition to the relatively poor identification of SVs, the short length of the region 3 sequence (290 bp) likely makes assigning taxa by homology-based BLASTN search more difficult (see S5 and S11 Tables), further indicating that region 3 may be unsuitable for DNA barcoding. The taxonomic and feeding habitat profiles of nematodes from the three sites studied were comparable in both regions 1 and 4 (see Figs 2 and 7), and these results were consistent with prior observations from DNA barcoding using individual nematodes [28]. Region 4 is thus likely the most suitable for DNA barcode-based taxonomic profiling of nematodes, due to the higher coverage of reference sequences than region 1 [36]. Since Sapkota and Nicolaisen [30] directly analyzed soil nematode communities using the semi-nested amplification in the NF1–18Sr2b region (i.e., region 4) from whole soil DNAs with the adopted primers (NemF and 18Sr2b) (S1 Fig), future steps should focus on testing the four primer sets studied here for DNA metabarcoding of nematodes using soil DNAs.

The nematode taxa of nematodes could also be analyzed using the high-throughput sequencing of amplicons generated from complex nematode DNAs by the universal eukaryotic primers instead of the nematode-specific primers. Geisen et al. used the universal eukaryotic primers 3NDf and 1132rmod for MiSeq-assisted sequencing of 570 bp-amplicons derived from isolated nematodes. The amplified DNA spans the V4-V5 regions including region 2 (S1 Fig) and the resultant taxonomic compositions of the nematodes were highly resolved despite the differences between the abusolute and relative abundances of the nematode taxa [57]. Bongiorno st al. also applied these primers for the taxonomic profiling of nematodes isolated from agricultural field soils [21]. Massive sequencing of long (>570 bp) PCR products by the 3NDf/132rmod primer set can provide high taxonomic resolution, however, recovery of the sequence reads will be reduced due to the limitations of the readable sequence length with the Illumina sequencing platform. This may cause issues for metabarcoding using amplicon sequencing from whole soil DNAs. As several universal eukaryotic primers have been developed across the SSU region [37, 38], universal primers for other SSU regions for amplicon sequencing should be examined using complex nematodes or soil DNAs.

In this study, although nematodes isolated from three different soils for DNA preparation were used, significant fractions of the reads from non-nematode eukaryotes were detected by amplicon sequencing in the four regions, especially in the house garden samples (Fig 1B). The majority of these reads were derived from the phylum Ascomycota and Basidiomycota (i.e., fungi), Arthropoda (i.e., insects), and Tardigrada. A large fraction of the sequence reads from the isolated nematodes were often occupied by non-nematode species in previous MiSeq-assisted amplicon sequencing. Approximately 5% of the total reads were only derived from the phyrum Nematode in the metabarcoding using the eukaryotic universal primers for the V4 and V9 regions and the nematodes from the forest soils, and most of the reads was derived from Fungi and Rhizaria [58]. Treonis et al. investigated the taxa of nematode communities from different types of agricultural soils by amplicon sequencing using NF1-18Sr2b primers with the Miseq platform [20]. In this study, the major fractions of the OTUs were derived from Fungi (40.3% in total eukaryotic OTUs) and Arthropoda (20.2%), and the relative abundance of the nematode-derived OTUs was 19.9%, but major fractions. Thus, even in isolated nematodes, non-nematode eukaryotes such as fungi and insects, were found to contaminate nematode samples as they made up large fractions of the reads and OTUs (SVs).

In this study, we succeeded in identifying novel nematode-derived SVs with less than 99% sequence identity to publicly available SSU-derived sequences (S7 Table). Over 40% of the nematode SVs in regions 1 and 2 were novel but less than 20% of those in the other two regions were novel. Since Ahmad et al. reported that the NF1-18Sr2b region (region 4) offers wide coverage compared with the SSUF04-SSUR22 region (region 1) [36], this difference was determined to be mainly caused by different sequence coverages in the public databases in the former and latter regions.

### Distinct taxonomic and ecophysiological structures and of nematode communities from the field, copse, and house garden soils

The taxonomic structures and ecophysiological functions (i.e., feeding types and life-history strategies) of nematodes in three distinct soils (uncultivated field, copse, and cultivated house garden) were investigated using DNA metabarcoding of four SSU gene regions. The highest and lowest fraction of nematode-derived SVs per total SVs were obtained in the field and house garden samples, respectively (Fig 1B). The most nematode SVs were obtained from the copse sample in regions 1 and 2 and from the field sample in regions 3 and 4. The fewest nematode SVs were found in the house garden sample in the SSU gene regions 1, 3, and 4 (see Table 2).

The profiles of the relative read abundances, taxa, and feeding types of the regional nematode SVs in four cladograms were useful for characterizing nematode communities (Figs 3–6). Communities from the field and copse samples were more complex than those in the house garden sample. The composition of the orders (Fig 2E–2H) and feeding types (Fig 7) also indicated a relatively simple structure of the nematode community in the house garden soil, dominated by Rhabditida-derived bacteria feeders. These results indicate the difference in complexity of nematode communities between the field and copse soils and the house garden soil.

Distinct taxonomic compositions of nematode SVs were found in each of the four SSU gene regions studied at the order (Fig 2) and family (S4–S8 Figs) levels among the three nematode communities, as were distinct compositions of feeding groups (Fig 7). The nematodes from the house garden soil were rich in Rhabditida (families Cephalobidae and Rhabditidae)-derived bacteria feeders and animal parasites. The community in the copse soil was formed by nematodes derived from the orders Dorylaimida (the family Belondiridae) and Rhabditida, which were mostly plant feeders. The nematode community in the uncultivated field soil was dominated by the Mononchida- and Dorylamida-derived omnivores and predators.

There have been several reports of taxonomic analyses of nematodes in forest [33, 58, 60–71] and agricultural field [11, 13, 15–17, 19–22, 30, 72] soils. Studies on forest soils have shown that bacteria, plant, and fungus feeders occupy significant fractions of the nematode communities despite varied proportions by the sampling sites, periods, and environmental status of forests [33, 62, 68, 70]. Omnivores and predators were found in relatively minor fractions except for in natural subtropical forests [58, 67] and coastal pine forests [70]. Prior high-throughput sequencing of SSU gene fragments using individual nematodes in copse soil demonstrated the presence of 18 rOTUs and three major orders (i.e., 51% of total nematodes were Dorylaimida, 29% were Rhabditida, and 17% were Triplonchida), and that over 70% of nematodes were fungus and plant feeders [28]. In this study, 32-to-88 nematode-derived SVs were detected, including 16 out of the above-mentioned 18 rOTUs from the nematode community of the comparable copse soil (see Table 2 and S9 Table). Further, plant feeders derived from the Dorylaimida and Rhabditida were also found to be the major factions of the community, although a decreased fraction of Triplonchida and fungus feeders were present (column Z in Figs 2E–2H and 7). These results also agreed with the abundant fractions of plant feeders found in previous taxonomic studies of nematode communities from Atlantic forest and steppe-forest soils using high-throughput amplicon sequencing [33, 58].

Nematode communities in agricultural fields have also been reported to contain mainly bacteria, fungus, and plant feeders, with minor groups of omnivores and predators [11, 13, 15–17, 19–22, 30, 72]. However, the proportions of feeding groups in agricultural soils can be modified by tillage [15, 20, 21], fertilizers and compost treatments [13, 15–17, 19, 20, 22], or crop cultivation [16, 17]. A prior investigation, in which Sanger-based DNA barcoding of individual nematodes from soybean-cultivated agricultural soils using a 900 bp-region encompassing regions 1 and 2 as a barcode (S1 Fig) allowed for the identification of 13 rOTUs, which were mainly classified to the Rhabditida-derived bacteria feeders [26]. In this study, 37 and 38 nematode-derived SVs, including 8 of the previously defined 13 rOTUs, were identified from amplicon sequencing of regions 1 and 2 using nematodes from uncultivated field soil at a comparable site (S2 and S10 Tables). However, despite the presence of common SVs in the previous rOTUs, nematodes derived from the orders Dorylaimida and Monochinda were more abundant in regions 1 and 4 in the field sample (column H in Fig 2E and 2H), as were those from the Rhabditida species in regions 2 and 3 (Fig 2F and 2G). Further, omnivores and predators occupied significant fractions of the field sample (Fig 7). In previous reports of amplicon sequencing for nematodes, relatively larger fractions (>40%) of bacterial feeders in the total population were detected in agricultural fields under the copping conditions with/without organic matter addition or tillage [21] or when fertilizer or farm compost were supplied [22]. These observations differed however, from those reported here for uncultivayed field soils. These differences may have been caused by the presence or absence of crop cultivation, fertilizer treatments, and tillage, which are known to affect the nematode community.

In an uncultivated field, the propagation of bacteria feeders may be restricted by the reduction of food; under a limited supply of fertilizers, predators and omnivores, which can subsist on a variety of food types, including other nematodes, may dominate the population. The taxa of nematodes from a zucchini-growing house garden were thus investigated here (Fig 1A). Across the four regions studied, the majority of the community was occupied by Rhabditida-derived bacteria feeders (column S in Figs 2 and 7), as has been often observed in nematode communities from cultivated agricultural soils. Additionally, distinct fractions of animal parasites were present in the house garden and copse samples (Fig 7); most of the parasites belonged to the families Ungellidae and Travassosinematidae (S5C–S8C Figs). Nematode species of these families have been reported to be parasitic to invertebrates such as earthworms [50–52]. These observations may indicate a bacteria-rich environment due to the supply of chemical fertilizers for zucchini cultivation and the presence of abundant host animals for parasitic nematodes.

The feeding behavior is a useful indicator for the ecological classification of nematodes [23, 55]. Nematodes can be also classified by life-history strategy, i.e., by assignment to colonizer–persister (cp) groups based on character sets that rank them from one to five [53]. Colonizers, with their lower cp-values, have short generation times, exploit transient niches, and have high rates of egg production, mobility, and metabolic activity; most bacteria feeders are included in this group. Persisters have higher cp-values, produce fewer eggs than colonizers, and are more susceptible to environmental disturbances. Lower cp groups are represented in broader terrestrial environments, whereas higher cp groups such as the Dorylaimida and Mononchida families generally have a narrower ecological amplitude [73]. Thus, the relative abundance of cp groups (i.e., cp-1–cp-5) in the nematode population has been used to assess terrestrial conditions and pollutions [23, 54, 55] and was also assessed here for the three soil samples studied (S4–S8 Figs and S11 Table). Bacteria-feeding species from the family Cephalobidiae (cp-2) dominated in the house garden soils, indicating high levels of microbial activity, presumably owing to the supply of chemical fertilizers. Dominant nematode families with low cp-values such as Rhabditidae (cp-1) and Cephalobidae (cp-2), have been commonly observed with Illumina-based amplicon sequencing of soil nematodes isolated from agricultural fields [20] that are supplied with fertilizers [22]. In the copse soils, however, nematodes from the family Belondiridae (cp-5) of the order Dorylaimida were commonly the most abundant across the regions, suggesting stable and undisturbed environments. Despite the different profiles between regions 1 and 4 (S5 and S8 Figs) and 2 and 3 (S6 and S7 Figs), both low (families Aphelenchidae (cp-2) and Rhabditidae (cp-1)) and high (the Dorylaimida- and Mononchida-derived families (cp-4 and cp-5, respectively)) cp groups were found in the uncultivated field sample. Although agricultural soils often contain an abundance of low-cp bacteria feeders due to the enriched nutrient conditions, predators, omnivores, and bacterivores may dominate the community via predations and feeding of available non-bacterial foods, including bacteria feeders, under the uncultivated and undisturbed conditions that occur for the several months without cultivation, tillage, or fertilizers.

Distinct nematode communities were also identified at the three sites using the maturity indices based on the cp-values and the frequency of the nematode families in the populations (Table 3). Low (1.58–2.73) and high (3.86–4.23) maturity indices were obtained from the house garden and copse soils, respectively. The maturity indcies of nematode communities calculated by the amplicon sequencing were previously found to be 1.4–2.8 [22] and 1.48–1.84 [21] in agricultural field soils, and 2.5–3.2 [33] in steppe-forest soils, and our results from the cropped garden and copse soils are consistent with these observations. The maturity indices from uncultivated field soils (2.71–3.63) were between the scoresfor the copse and house garden samples, supporting the above discussion.

## Conclusions

In this study, the taxonomic structures of three nematode communities from uncultivated field, copse, and cultivated house garden soils were investigated along with their feeding types and life-history strategies by high-throughput amplicon sequencing of four regions (regions 1–4) of the 18S ribosomal RNA gene. We also investigated the suitable regions for DNA metabarcoding for the complex nematode populations among the four SSU regions. The fewest nematode-derived SVs were detected in region 3, but their contents in total SVs were highest among four regions; this is consistent with prior DNA barcoding of individual nematodes. The most nematode-derived SVs were found in the copse and house garden samples in region 2, likely due in part to the increased abundance of Rhabditida-derived SVs. Consistent with prior work, the relative read abundances in regions 1 and 4 were comparable in both nematode orders and feeding groups, suggesting that region 4 is a suitable target for DNA barcoding of nematode communities and individual nematodes.

The performed BLASTN search and developed cladograms of regional nematode SVs in the four SSU gene regions studied clarified distinct taxonomic structures of nematodes living in the three soil types studied. In the uncultivated field soil, nematode SVs derived from the Dorylaimida, Mononchida, and Rhabditida orders were most abundant, whereas those from the Dorylaimida and Rhabditida orders were most abundant in the copse soil and those from the Rhabditida order were most abundant in the house garden soil. Further, distinct ecophysiological community structures among the soils were successfully detected via varying feeding habitats and life-history strategies. House garden soil was dominated by the family Cephalobidae (cp-2) and Rhabditidae (cp-1)-derived bacteria feeders and animal parasites, whereas copse soil had more Belondiridae (cp-5)-derived plant feeders. This suggests the presence of abundantly propagated bacteria in the house garden soil and ecologically undisturbed and plant-rich conditions in the copse soil. In the uncultivated field, families with low cp-values as well as a significant fraction of families with high cp-values such as Mylonchulidae (cp-4) were detected, suggesting a possible transient nematode community status including bacteria feeders, omnivores, and predators. Distinct ecophysiological structures for the three nematode communities were also indicated by the maturity indices for the three sampling sites: low, high, and middle maturity indices were obtained from the house garden, copse and uncultivated field samples, respectively. Finally, a comparison of tail sequences demonstrated that the influences in DNA barcoding by tail sequences upon amplification were not significant. Overall, the taxa and functional groups of soil nematodes derived from different environmental soils were clarified using high-throughput amplicon sequencing of four SSU gene regions; future efforts should focus on applying this approach to the taxonomic and functional profiling of nematodes as well as other soil organisms by using whole soil DNAs.

## Supporting information

S1 Table Information of the DRA-registered sequende data. (PDF)

S2 Table Nematode-derived sequence variants (SVs) identified by SILVA-based taxonomic ranks and their relative abundances in the three soil samples studied. (PDF)

S3 Table Nematode-derived SVs from region 1 and their taxa and feeding types based on a BLASTN search and SILVA database. (PDF)

S4 Table Nematode-derived SVs from region 2 and their taxa and feeding types based on a BLASTN search and SILVA database. (PDF)

S5 Table Nematode-derived SVs from region 3 and their taxa and feeding types based on a BLASTN search and SILVA database. (PDF)

S6 Table Nematode-derived SVs from region 4 and their taxa and feeding types based on a BLASTN search and SILVA database. (PDF)

S7 Table Novel nematode-derived SVs from three nematode communities in four SSU regions. (PDF)

S8 Table Regional nematode SVs shared with high sequence similarities. (PDF)

S9 Table Regional nematode SVs identical to the previously determined [28] gene-derived operational taxonomic units (rOTUs) found using the copse-derived nematodes (Z01). (PDF)

S10 Table Nematode SVs in regions 1 and 2 identical to the previously determined [26] rOTUs using the flowerbed (K01)- and cultivated field (H01)-derived nematodes. (PDF)

S11 Table Regional nematode SVs, feeding types, and colonizer–persister (cp)-values in the families of the four 18S small subunit ribosomal RNA (SSU) regions. (PDF)

S1 Fig Four barcode regions of the 18S ribosomal RNA (SSU) gene. The four barcode regions for PCR amplifications (regions 1–4, with amplicon sizes shown) used in this and in a prior study [28] are indicated by black double-headed arrows. The numbers indicating the nucleotide positions of the 5’-end of forward primers are shown on the entire SSU gene prepared from the nucleotide sequence of the *C*. *elegans* ribosomal RNA gene cluster (X03680). The dark blue boxes correspond to the hypervariable regions of the eukaryotic SSU genes reported by Hugerth et al. [38] and Hadziavdic et al. [37]. The regions that were amplified by the four indicated published primer sets (SSUF04-SSUR22 [39], EcoF-EcoR [32], NF1-18Sr2b [29], and NemF-18Sr2b [30]), as well as the amplified region from prior Sanger-based DNA barcoding [26], are indicated using gray double-headed arrows with amplicon sizes in parenthesis.
(TIFF)

S2 Fig Experimental scheme including comparative data analyses with prior results. The experimental scheme is shown in a box by solid line. The resultant nematode-derived SVs identified in this study were used for taxonomic profiling and investigation of primer tails’s effects on amplifications. The SSU-derived operational taxonomic units (rOTUs) identified in prior MiSeq [28]- and Sanger [26]-based DNA barcoding of individual nematodes are shown in boxes (broken lines) and were integrated into the cladograms of regional nematode SVs to compare the taxomic profiles (see Figs 3–6). Red colored codes in parenthesis (e.g., H02) represent the soil sample code (H: field soil) and the experimental ID (ID: 02).
(TIFF)

S3 Fig PCR products amplified from the three nematode sample DNAs in the four SSU regions. Five microliter aliquots of each reaction mixture containing nematode DNA from the (A) copse, (B) house garden and (C) field soils were subjected to 1% (A and B) or 2% (C) agarose gel electrophoresis. The PCR products from region 1–4 in the gels are visualized using successive ethidium bromide staining and are shown in lanes 1–4, respectively. In the field sample, the products prepared from one-step PCR with tailed (Tail) and two-step PCR with tailless and tailed primers (No_tail) were analyzed. M: Gene Ladder Wide 1 (Nippon Gene, Toyama, Japan) as a size marker.
(TIFF)

S4 Fig Regional nematode SVs by family and cp-group. The number of nematode SVs identified from the field (blue), copse (green), and house garden (dark red) soil samples is indicated by 3D-histogram for regions (A–D) 1–4, respectively, in each family. The cp-values, shown at the bottom of (D), indicate the nematode’s life strategy characteristics as described in the Materials and methods section. NA: not assigned to a single family (i.e., assigned to multiple families).
(TIFF)

S5 Fig Relative read abundance of region 1-derived nematode SVs by family and cp-group. The relative abundance (%) of sequence reads of SVs in region 1 from the (A) field, (B) copse, and (C) house garden samples for each family. Families are aligned by their cp-values (1–5); undefined cp-values are indicated by a hyphen (-). NA: not assigned to a single family.
(TIFF)

S6 Fig Relative read abundance of region 2-derived nematode SVs by family and cp-group. The relative abundance (%) of sequence reads of SVs in region 2 from the (A) field, (B) copse, and (C) house garden samples for each family. Families are aligned by their cp-values (1–5); undefined cp-values are indicated by a hyphen (-). NA: not assigned to a single family.
(TIFF)

S7 Fig Relative read abundance of region 3-derived nematode SVs by family and cp-group. The relative abundance (%) of sequence reads of SVs in region 3 from the (A) field, (B) copse, and (C) house garden samples for each family. Families are aligned by their cp-values (1–5); undefined cp-values are indicated by a hyphen (-). NA: not assigned to a single family.
(TIFF)

S8 Fig Relative read abundance of region 4-derived nematode SVs by family and cp-group. The relative abundance (%) of sequence reads of SVs in region 4 from the (A) field, (B) copse, and (C) house garden samples for each family. Families are aligned by their cp-values (1–5); undefined cp-values are indicated by a hyphen (-). NA: not assigned to a single family.
(TIFF)

S9 Fig Relative abundances and phylum of SVs by SSU gene region obtained from the high-throughput sequencing of amplicons prepared from one-step PCR with tailed primers and two-step PCR with tailless and tailed primers. The SVs from each SSU gene region shown on top of the histograms were identified from the high-throughput sequencing of amplicons prepared from one-step PCR with tailed primers (Tail) and two-step PCR with tailless and tailed primers (No_tail). The relative abundance of SVs and their phylum are shown, where the phylum is indicated by the colors in the legend. NA: Not assigned.
(TIFF)

S10 Fig Relative read abundances and orders of regional nematode SVs from amplicons of each SSU gene region generated by one- and two-step PCRs. The regional nematode SVs are indicated to the left of the column with each SSU gene region on top and their relative abundance of sequence reads in the amplicons from one-step PCR (Tail) and two-step PCR (No_tail) are indicated in each SSU gene region by density bars as shown in the legend. The colors represent the orders of the SVs determined by BLASTN search as indicated in the legend.
(TIFF)

S11 Fig (TIFF)

S1 Raw images The amplification of each SSU gene region of nematode DNA from the copse (left image of the first figure) and house garden (right image) soils was performed by PCR with tailed PCR primers for each SSU gene region. Aliquots of the resultant PCR products from each sample were independently subjected to 1% agarose gel electrophoresis as shown in the figure, where a red “X” designates an empty lane. The PCR products from one-step PCR (right side of the second figure) and two-step PCR (left side of the second figure, including a marker lane) were amplified using the field sample and were subjected to 2% agarose gel electrophoresis. The PCR products in the three gels were visualized using successive ethidium bromide staining. Fluorescent images of agarose gels were acquired using the FAS-III gel imaging system (Nippon Genetics Co., Tokyo, Japan), and the original TIFF images shown in each file were used to prepare S3A–S3C Fig by cropping out the unrelated area. M: lane with a size marker (Gene Ladder Wide 1, Nippon Gene, Toyama, Japan).
(PDF)
