# Supplementary material for: Distinct community structures of soil nematodes from three ecologically different sites revealed by high-throughput amplicon sequencing of four 18S ribosomal RNA gene regions
Source: PLoS One. 2021 Apr 15;16(4):e0249571. doi: 10.1371/journal.pone.0249571 (PMC8049254; doi:10.1371/journal.pone.0249571)
Supplement: S1 Table — (PDF) [file pone.0249571.s001.pdf]

S1 Table. Information of the DRA-registered sequende data.

ID	Sample_name	Type	Year	Platform	Read_length	Target_gene	Region	Tail	Primer	Filename_R1	Filename_R2
TE1	field_H02	field	2020	MiSeq	300bpx2	18S rDNA	region1	notail	primerSSU18A-4F3/SSU_R22	TE1_R1.fastq	TE1_R2.fastq
TE2	field_H02	field	2020	MiSeq	300bpx2	18S rDNA	region1	tail	primerSSU18A-4F3_MiseqF/SSU_R22_MiseqR	TE2_R1.fastq	TE2_R2.fastq
TE3	field_H02	field	2020	MiSeq	300bpx2	18S rDNA	region2	notail	primerSSU_consF/SSU26Rplus4	TE3_R1.fastq	TE3_R2.fastq
TE4	field_H02	field	2020	MiSeq	300bpx2	18S rDNA	region2	tail	primerSSU_consF_MiseqF/SSU26Rplus4_MiseqR	TE4_R1.fastq	TE4_R2.fastq
TE5	field_H02	field	2020	MiSeq	300bpx2	18S rDNA	region3	notail	primerNem_18SR_ExtF/SSU_R23plus7	TE5_R1.fastq	TE5_R2.fastq
TE6	field_H02	field	2020	MiSeq	300bpx2	18S rDNA	region3	tail	primerNem_18SR_ExtF_MiseqF/SSU_R23plus7_MiseqR	TE6_R1.fastq	TE6_R2.fastq
TE7	field_H02	field	2020	MiSeq	300bpx2	18S rDNA	region4	notail	primerNF1/18Sr2b_ExtR	TE7_R1.fastq	TE7_R2.fastq
TE8	field_H02	field	2020	MiSeq	300bpx2	18S rDNA	region4	tail	primerNF1_MiseqF/18Sr2b_ExtR_MiseqR	TE8_R1.fastq	TE8_R2.fastq
TE9	housegarden_S01	housegarden	2020	MiSeq	300bpx2	18S rDNA	region1	tail	primerSSU18A-4F3_MiseqF/SSU_R22_MiseqR	TE9_R1.fastq	TE9_R2.fastq
TE10	housegarden_S01	housegarden	2020	MiSeq	300bpx2	18S rDNA	region2	tail	primerSSU_consF_MiseqF/SSU26Rplus4_MiseqR	TE10_R1.fastq	TE10_R2.fastq
TE11	housegarden_S01	housegarden	2020	MiSeq	300bpx2	18S rDNA	region3	tail	primerNem_18SR_ExtF_MiseqF/SSU_R23plus7_MiseqR	TE11_R1.fastq	TE11_R2.fastq
TE12	housegarden_S01	housegarden	2020	MiSeq	300bpx2	18S rDNA	region4	tail	primerNF1_MiseqF/18Sr2b_ExtR_MiseqR	TE12_R1.fastq	TE12_R2.fastq
TE13	copse_Z02	copse	2020	MiSeq	300bpx2	18S rDNA	region1	tail	primerSSU18A-4F3_MiseqF/SSU_R22_MiseqR	TE13_R1.fastq	TE13_R2.fastq
TE14	copse_Z02	copse	2020	MiSeq	300bpx2	18S rDNA	region2	tail	primerSSU_consF_MiseqF/SSU26Rplus4_MiseqR	TE14_R1.fastq	TE14_R2.fastq
TE15	copse_Z02	copse	2020	MiSeq	300bpx2	18S rDNA	region3	tail	primerNem_18SR_ExtF_MiseqF/SSU_R23plus7_MiseqR	TE15_R1.fastq	TE15_R2.fastq
TE16	copse_Z02	copse	2020	MiSeq	300bpx2	18S rDNA	region4	tail	primerNF1_MiseqF/18Sr2b_ExtR_MiseqR	TE16_R1.fastq	TE16_R2.fastq
