# Supplementary material for: Distinct community structures of soil nematodes from three ecologically different sites revealed by high-throughput amplicon sequencing of four 18S ribosomal RNA gene regions
Source: PLoS One. 2021 Apr 15;16(4):e0249571. doi: 10.1371/journal.pone.0249571 (PMC8049254; doi:10.1371/journal.pone.0249571)
Supplement: S2 Table — (PDF) [file pone.0249571.s002.pdf]

S2 Table. Nematode-derived sequence variants (SVs) identified by SILVA-based taxonomic ranks and their relative abundances in the three soil samples studied.

Region 1

R1_SV	Field	Copse	Garden
R1_SV_3	35.2092	0.0000	0.0000
R1_SV_4	30.5869	0.0000	0.0000
R1_SV_5	0.0000	36.2410	0.1909
R1_SV_10	5.4623	2.0717	0.0000
R1_SV_12	2.3220	10.6450	1.7751
R1_SV_14	3.0663	0.0000	0.0000
R1_SV_22	0.0000	7.6522	0.4581
R1_SV_23	0.0000	7.1104	0.0000
R1_SV_24	0.0000	3.9035	20.1756
R1_SV_27	2.5418	0.0000	0.0000
R1_SV_30	2.0195	0.0000	0.9162
R1_SV_32	0.0000	4.3318	6.2035
R1_SV_33	1.4450	0.0000	7.3487
R1_SV_34	2.1022	0.0000	0.0000
R1_SV_36	0.0762	4.7188	0.0000
R1_SV_37	2.1545	0.0000	0.0000
R1_SV_41	2.0979	0.0000	0.0000
R1_SV_43	1.1012	0.0000	0.0000
R1_SV_44	0.0000	4.0764	0.0000
R1_SV_47	1.7214	0.0000	0.0000
R1_SV_48	0.1175	1.2926	18.7631
R1_SV_54	1.4146	0.0000	0.0000
R1_SV_57	0.0000	1.6512	9.7538
R1_SV_62	1.3906	0.0000	0.0000
R1_SV_67	0.0000	2.6961	0.0000
R1_SV_69	1.1991	0.0000	0.0000
R1_SV_70	0.3874	1.6512	0.0000
R1_SV_80	0.0000	1.9195	0.0000
R1_SV_81	0.7660	0.0000	0.6108
R1_SV_84	0.7900	0.0000	0.9353
R1_SV_87	0.0000	1.5093	1.2598
R1_SV_100	0.0000	1.0784	0.0000
R1_SV_104	0.0000	1.0372	0.0000
R1_SV_106	0.0000	0.0000	7.4442
R1_SV_110	0.0000	0.9365	0.0000
R1_SV_116	0.0000	0.0000	5.8599
R1_SV_119	0.0000	0.4051	2.4814
R1_SV_121	0.3874	0.0000	0.0000
R1_SV_123	0.0000	0.0000	5.1537
R1_SV_124	0.0000	0.6785	0.0000
R1_SV_132	0.0000	0.6244	0.0000
R1_SV_134	0.0000	0.6192	0.0000
R1_SV_152	0.3025	0.0000	0.0000
R1_SV_169	0.0000	0.3999	0.0000
R1_SV_170	0.0000	0.3999	0.0000
R1_SV_171	0.1980	0.0000	0.0000
R1_SV_178	0.0000	0.3741	0.0000
R1_SV_180	0.1088	0.0000	0.0000
R1_SV_184	0.0000	0.1471	1.3934
R1_SV_188	0.0000	0.0000	2.3478
R1_SV_194	0.1458	0.0000	0.0000
R1_SV_197	0.0000	0.0000	2.1951
R1_SV_200	0.0000	0.2915	0.0000
R1_SV_205	0.2416	0.0000	0.0000
R1_SV_217	0.1654	0.0000	0.0000
R1_SV_221	0.0000	0.2322	0.0000
R1_SV_231	0.0000	0.2167	0.0000
R1_SV_233	0.0000	0.0000	1.5843
R1_SV_291	0.0000	0.0000	1.0116
R1_SV_293	0.0392	0.0000	0.0000
R1_SV_295	0.1088	0.0000	0.0000
R1_SV_298	0.0000	0.1264	0.0000
R1_SV_304	0.0000	0.1238	0.0000
R1_SV_310	0.0000	0.0000	0.8780
R1_SV_312	0.0000	0.1135	0.0000
R1_SV_316	0.0000	0.1109	0.0000
R1_SV_322	0.0000	0.1058	0.0000
R1_SV_326	0.0000	0.1032	0.0000
R1_SV_351	0.0305	0.0000	0.0000
R1_SV_353	0.0762	0.0000	0.0000
R1_SV_371	0.0631	0.0000	0.0000
R1_SV_386	0.0000	0.0671	0.0000
R1_SV_396	0.0000	0.0310	0.0000
R1_SV_400	0.0000	0.0000	0.4772
R1_SV_413	0.0000	0.0000	0.4199
R1_SV_418	0.0000	0.0568	0.0000
R1_SV_424	0.0283	0.0000	0.0000
R1_SV_437	0.0000	0.0516	0.0000
R1_SV_439	0.0000	0.0490	0.0000
R1_SV_455	0.0000	0.0464	0.0000
R1_SV_462	0.0370	0.0000	0.0000
R1_SV_476	0.0348	0.0000	0.0000
R1_SV_493	0.0000	0.0335	0.0000
R1_SV_507	0.0000	0.0310	0.0000
R1_SV_521	0.0000	0.0000	0.2100
R1_SV_543	0.0218	0.0000	0.0000
R1_SV_551	0.0196	0.0000	0.0000
R1_SV_556	0.0196	0.0000	0.0000
R1_SV_562	0.0000	0.0000	0.1527
R1_SV_578	0.0000	0.0155	0.0000
R1_SV_594	0.0000	0.0129	0.0000
R1_SV_600	0.0000	0.0103	0.0000

Region 2

R2_SV	Field	Copse	Garden
R2_SV_1	55.8161	0.0000	0.0000
R2_SV_3	19.6531	0.0000	0.0000
R2_SV_5	10.9975	0.0000	0.0000
R2_SV_10	0.0000	25.7343	0.1576
R2_SV_12	0.0000	11.3581	12.3906
R2_SV_15	0.8624	8.7183	1.2880
R2_SV_17	2.4911	2.6486	0.0000
R2_SV_18	0.0000	3.5338	20.0967
R2_SV_19	0.0000	8.5054	0.0000
R2_SV_21	0.1654	0.8905	21.5314
R2_SV_24	0.0000	6.7631	0.0000
R2_SV_25	0.0000	4.1339	7.2007
R2_SV_26	0.0000	5.7776	0.0000
R2_SV_30	0.0000	4.2060	0.0000
R2_SV_31	1.2896	0.0000	0.0000
R2_SV_35	0.0000	3.2346	0.0000
R2_SV_36	0.9275	0.0000	0.0380
R2_SV_38	0.8828	0.0000	0.4293
R2_SV_41	1.0103	0.0000	0.0000
R2_SV_42	0.9221	0.0000	0.0000
R2_SV_47	0.0000	1.6525	2.0053
R2_SV_48	0.5845	0.0000	1.9347
R2_SV_52	0.9018	0.0000	0.0000
R2_SV_53	0.7214	0.0000	0.2446
R2_SV_54	0.0000	0.0000	6.2062
R2_SV_56	0.6956	0.0000	0.0000
R2_SV_63	0.0000	0.0000	5.1899
R2_SV_72	0.0000	0.0000	4.1737
R2_SV_89	0.2834	0.0000	0.5652
R2_SV_90	0.0000	0.0000	2.9835
R2_SV_95	0.0000	0.9151	0.0000
R2_SV_102	0.2468	0.0000	0.0000
R2_SV_103	0.0000	0.0000	2.5325
R2_SV_105	0.2753	0.0000	0.0000
R2_SV_110	0.2699	0.0000	0.0000
R2_SV_114	0.0000	0.3414	1.0760
R2_SV_116	0.0000	0.6793	0.0000
R2_SV_120	0.0000	0.6195	0.0000
R2_SV_127	0.0000	0.5772	0.0000
R2_SV_128	0.0000	0.2640	0.9619
R2_SV_129	0.0000	0.5720	0.0000
R2_SV_131	0.0000	0.5667	0.0000
R2_SV_132	0.0000	0.5579	0.0000
R2_SV_134	0.1614	0.0000	0.0000
R2_SV_135	0.0000	0.1883	1.1141
R2_SV_139	0.0000	0.5227	0.0000
R2_SV_140	0.0000	0.0000	1.5434
R2_SV_142	0.2075	0.0000	0.0000
R2_SV_145	0.0000	0.3678	0.2935
R2_SV_147	0.1641	0.0000	0.0000
R2_SV_150	0.0000	0.3731	0.0000
R2_SV_164	0.0719	0.0000	0.3152
R2_SV_168	0.0000	0.3080	0.0000
R2_SV_176	0.0000	0.0000	0.9021
R2_SV_179	0.1126	0.0000	0.0000
R2_SV_180	0.0000	0.2816	0.0000
R2_SV_186	0.0244	0.2182	0.0000
R2_SV_190	0.0000	0.0000	0.7554
R2_SV_198	0.0000	0.0000	0.7228
R2_SV_199	0.0000	0.2323	0.0000
R2_SV_200	0.0000	0.2253	0.0000
R2_SV_202	0.0000	0.2217	0.0000
R2_SV_205	0.0000	0.2094	0.0000
R2_SV_208	0.0380	0.0000	0.0000
R2_SV_209	0.0000	0.2024	0.0000
R2_SV_214	0.0000	0.1936	0.0000
R2_SV_215	0.0000	0.0000	0.5924
R2_SV_217	0.0000	0.1865	0.0000
R2_SV_222	0.0000	0.1813	0.0000
R2_SV_237	0.0000	0.1689	0.0000
R2_SV_238	0.0000	0.1672	0.0000
R2_SV_243	0.0000	0.1549	0.0000
R2_SV_246	0.0000	0.1461	0.0000
R2_SV_247	0.0000	0.0000	0.4511
R2_SV_253	0.0000	0.0792	0.2011
R2_SV_259	0.0000	0.1390	0.0000
R2_SV_260	0.0000	0.1390	0.0000
R2_SV_262	0.0000	0.0000	0.4130
R2_SV_265	0.0000	0.1320	0.0000
R2_SV_270	0.0000	0.1214	0.0000
R2_SV_272	0.0000	0.0282	0.2826
R2_SV_273	0.0000	0.1179	0.0000
R2_SV_275	0.0000	0.1162	0.0000
R2_SV_279	0.0000	0.1144	0.0000
R2_SV_281	0.0000	0.0000	0.3424
R2_SV_282	0.0000	0.1109	0.0000
R2_SV_288	0.0000	0.1074	0.0000
R2_SV_291	0.0000	0.1038	0.0000
R2_SV_296	0.0000	0.1003	0.0000
R2_SV_299	0.0000	0.0968	0.0000
R2_SV_300	0.0000	0.0950	0.0000
R2_SV_301	0.0000	0.0950	0.0000

Region 2 (continued)

R2_SV	Field	Copse	Garden
R2_SV_303	0.0000	0.0950	0.0000
R2_SV_304	0.0000	0.0669	0.0815
R2_SV_315	0.0000	0.0845	0.0000
R2_SV_317	0.0244	0.0000	0.0000
R2_SV_324	0.0000	0.0792	0.0000
R2_SV_329	0.0000	0.0739	0.0000
R2_SV_334	0.0000	0.0704	0.0000
R2_SV_336	0.0000	0.0704	0.0000
R2_SV_337	0.0000	0.0686	0.0000
R2_SV_338	0.0000	0.0686	0.0000
R2_SV_343	0.0000	0.0669	0.0000
R2_SV_349	0.0000	0.0634	0.0000
R2_SV_351	0.0000	0.0634	0.0000
R2_SV_355	0.0000	0.0598	0.0000
R2_SV_362	0.0000	0.0211	0.1141
R2_SV_363	0.0000	0.0581	0.0000
R2_SV_369	0.0000	0.0000	0.1685
R2_SV_371	0.0000	0.0546	0.0000
R2_SV_386	0.0380	0.0000	0.0000
R2_SV_390	0.0000	0.0000	0.1522
R2_SV_404	0.0000	0.0000	0.1413
R2_SV_410	0.0000	0.0440	0.0000
R2_SV_411	0.0000	0.0440	0.0000
R2_SV_414	0.0000	0.0422	0.0000
R2_SV_415	0.0000	0.0422	0.0000
R2_SV_417	0.0000	0.0000	0.0272
R2_SV_418	0.0000	0.0405	0.0000
R2_SV_427	0.0298	0.0000	0.0000
R2_SV_430	0.0000	0.0387	0.0000
R2_SV_433	0.0000	0.0370	0.0000
R2_SV_443	0.0258	0.0000	0.0000
R2_SV_448	0.0000	0.0317	0.0000
R2_SV_450	0.0000	0.0317	0.0000
R2_SV_452	0.0000	0.0317	0.0000
R2_SV_461	0.0231	0.0000	0.0000
R2_SV_470	0.0000	0.0000	0.0815
R2_SV_471	0.0203	0.0000	0.0000
R2_SV_476	0.0095	0.0000	0.0000
R2_SV_483	0.0000	0.0246	0.0000
R2_SV_490	0.0000	0.0000	0.0706
R2_SV_499	0.0000	0.0000	0.0652
R2_SV_500	0.0000	0.0000	0.0652
R2_SV_505	0.0149	0.0000	0.0000
R2_SV_517	0.0136	0.0000	0.0000
R2_SV_519	0.0000	0.0000	0.0543
R2_SV_531	0.0000	0.0000	0.0435
R2_SV_549	0.0095	0.0000	0.0000
R2_SV_558	0.0000	0.0106	0.0000
R2_SV_559	0.0081	0.0000	0.0000
R2_SV_563	0.0000	0.0106	0.0000
R2_SV_576	0.0068	0.0000	0.0000
R2_SV_584	0.0000	0.0070	0.0000
R2_SV_594	0.0000	0.0035	0.0000

Region 3

R3_SV	Field	Copse	Garden
R3_SV_1	33.9415	3.8212	4.9750
R3_SV_2	18.8314	0.0000	0.0000
R3_SV_4	22.3052	0.6061	29.8343
R3_SV_5	0.0000	28.3100	0.0000
R3_SV_7	5.0038	1.7272	0.0000
R3_SV_10	0.0000	11.8283	8.0798
R3_SV_11	1.2357	9.6318	0.7866
R3_SV_12	1.4224	1.1914	15.5032
R3_SV_13	3.3519	0.0000	1.1200
R3_SV_14	0.0000	11.3362	0.0000
R3_SV_15	2.5674	0.0000	0.6512
R3_SV_17	0.0000	4.3513	15.2323
R3_SV_19	2.0837	0.0000	0.0000
R3_SV_22	0.0000	5.5617	0.0000
R3_SV_25	1.5314	0.0000	0.0000
R3_SV_26	1.4004	0.0000	0.0000
R3_SV_27	0.9388	0.0000	3.3913
R3_SV_28	0.0000	4.7218	0.0000
R3_SV_29	0.0000	4.5793	0.0000
R3_SV_30	0.0000	2.6336	1.6462
R3_SV_32	0.0000	2.9205	0.0000
R3_SV_34	1.0283	0.0000	3.4538
R3_SV_36	0.7832	0.0000	0.0000
R3_SV_39	1.4419	0.0000	0.0000
R3_SV_40	0.0000	0.0000	5.5845
R3_SV_42	0.0000	0.8722	2.1932
R3_SV_43	0.2749	0.0000	0.0000
R3_SV_44	0.2023	0.0000	0.0000
R3_SV_45	0.0000	1.2427	0.4793
R3_SV_47	0.4059	0.0000	0.4115
R3_SV_48	0.3371	0.0000	0.0000
R3_SV_51	0.0000	1.2427	0.0000
R3_SV_53	0.0000	0.0000	2.7454
R3_SV_54	0.2736	0.0000	0.0000
R3_SV_58	0.0000	0.8266	0.0000
R3_SV_65	0.0000	0.0000	1.2711
R3_SV_68	0.0000	0.4218	0.0000
R3_SV_69	0.0000	0.3914	0.0000
R3_SV_72	0.0000	0.3648	0.0000
R3_SV_78	0.0454	0.0532	0.0000
R3_SV_83	0.1102	0.0000	0.0000
R3_SV_84	0.0000	0.2432	0.0000
R3_SV_86	0.0000	0.0000	0.6251
R3_SV_87	0.0000	0.0000	0.6199
R3_SV_88	0.0000	0.2185	0.0000
R3_SV_89	0.0000	0.0000	0.5991
R3_SV_90	0.0000	0.0000	0.5991
R3_SV_92	0.1271	0.0000	0.0000
R3_SV_95	0.0000	0.1767	0.0000
R3_SV_98	0.0000	0.1634	0.0000
R3_SV_100	0.0493	0.0000	0.0000
R3_SV_103	0.0558	0.0000	0.0000
R3_SV_104	0.0000	0.1387	0.0000
R3_SV_107	0.0000	0.1140	0.0000
R3_SV_113	0.0000	0.0988	0.0000
R3_SV_122	0.0000	0.0836	0.0000
R3_SV_123	0.0532	0.0000	0.0000
R3_SV_126	0.0000	0.0703	0.0000
R3_SV_129	0.0441	0.0000	0.0000
R3_SV_132	0.0000	0.0570	0.0000
R3_SV_141	0.0000	0.0000	0.1354
R3_SV_144	0.0311	0.0000	0.0000
R3_SV_148	0.0259	0.0000	0.0000
R3_SV_149	0.0259	0.0000	0.0000
R3_SV_151	0.0246	0.0000	0.0000
R3_SV_153	0.0233	0.0000	0.0000
R3_SV_158	0.0220	0.0000	0.0000
R3_SV_174	0.0000	0.0000	0.0625
R3_SV_192	0.0013	0.0000	0.0000

Region 4

R4_SV	Field	Copse	Garden
R4_SV_1	24.0870	0.0000	0.0000
R4_SV_2	38.9976	0.1595	0.0000
R4_SV_7	0.0000	28.9025	0.1405
R4_SV_12	0.0000	0.6616	40.0522
R4_SV_14	0.0000	14.1604	7.8900
R4_SV_15	6.5730	0.3144	0.0000
R4_SV_17	1.8065	9.5763	0.8332
R4_SV_18	0.8508	0.0000	0.0000
R4_SV_24	0.3745	0.6522	11.2728
R4_SV_25	0.0000	8.6661	0.0000
R4_SV_26	2.9210	0.0000	0.0000
R4_SV_27	2.8012	0.0000	0.3313
R4_SV_30	0.0000	3.1765	8.9239
R4_SV_31	2.2799	0.0000	0.0000
R4_SV_33	2.3787	0.0000	0.0000
R4_SV_34	0.0000	4.0445	4.1257
R4_SV_36	1.4410	0.0000	1.3351
R4_SV_37	1.5698	0.0000	0.0000
R4_SV_38	0.0000	4.6169	1.9273
R4_SV_39	0.0000	5.4052	0.0000
R4_SV_41	2.2709	0.0000	0.0000
R4_SV_42	1.5339	0.0000	1.4455
R4_SV_43	1.6627	0.0000	0.0000
R4_SV_45	0.0000	4.3588	0.0000
R4_SV_47	0.0000	4.3260	0.0000
R4_SV_57	0.0000	1.3184	3.9048
R4_SV_58	0.0719	2.9935	0.0000
R4_SV_59	1.0036	0.0000	0.0000
R4_SV_62	0.7310	0.0000	0.0000
R4_SV_63	1.2103	0.0000	0.0000
R4_SV_70	0.0000	1.0463	2.2184
R4_SV_73	0.0000	0.0000	4.3666
R4_SV_76	0.8029	0.0000	0.1907
R4_SV_86	0.6321	0.0000	0.6926
R4_SV_87	0.5932	0.0000	0.0000
R4_SV_93	0.5482	0.0000	0.0000
R4_SV_97	0.5512	0.0000	0.6826
R4_SV_103	0.0000	0.0000	2.4092
R4_SV_108	0.3056	0.0000	0.0000
R4_SV_111	0.0000	0.0000	2.0377
R4_SV_112	0.0000	0.9290	0.0000
R4_SV_117	0.0000	0.8868	0.0000
R4_SV_118	0.3236	0.0000	0.0000
R4_SV_121	0.0000	0.8586	0.0000
R4_SV_129	0.0000	0.0000	1.5459
R4_SV_133	0.2247	0.0000	0.0000
R4_SV_149	0.0000	0.5302	0.0000
R4_SV_150	0.3355	0.0000	0.0000
R4_SV_158	0.3026	0.0000	0.0000
R4_SV_159	0.0000	0.4598	0.0000
R4_SV_161	0.0000	0.0000	0.9335
R4_SV_168	0.0000	0.2158	0.3915
R4_SV_170	0.1348	0.0000	0.0000
R4_SV_175	0.1768	0.0000	0.0000
R4_SV_182	0.0000	0.3237	0.0000
R4_SV_184	0.0000	0.0000	0.6926
R4_SV_186	0.0000	0.0000	0.6826
R4_SV_208	0.1198	0.0000	0.0000
R4_SV_215	0.0000	0.2393	0.0000
R4_SV_219	0.0000	0.0000	0.5019
R4_SV_222	0.1438	0.0000	0.0000
R4_SV_233	0.0000	0.0000	0.4216
R4_SV_235	0.0000	0.1971	0.0000
R4_SV_237	0.0000	0.1877	0.0000
R4_SV_246	0.0000	0.1642	0.0000
R4_SV_251	0.0629	0.0000	0.0000
R4_SV_255	0.0000	0.1548	0.0000
R4_SV_273	0.0000	0.1314	0.0000
R4_SV_287	0.0000	0.1126	0.0000
R4_SV_293	0.0000	0.1079	0.0000
R4_SV_296	0.0539	0.0000	0.0502
R4_SV_326	0.0509	0.0000	0.0000
R4_SV_335	0.0000	0.0704	0.0000
R4_SV_338	0.0419	0.0000	0.0000
R4_SV_360	0.0000	0.0516	0.0000
R4_SV_372	0.0300	0.0000	0.0000

Note: The SVs indicated by yellow were assigned to nematode-derived SVs by SILVA database, but were demonstrated to be derived from a non-nematode species by BLASTN search.
