# Supplementary material for: Distinct community structures of soil nematodes from three ecologically different sites revealed by high-throughput amplicon sequencing of four 18S ribosomal RNA gene regions
Source: PLoS One. 2021 Apr 15;16(4):e0249571. doi: 10.1371/journal.pone.0249571 (PMC8049254; doi:10.1371/journal.pone.0249571)
Supplement: S3 Table — (PDF) [file pone.0249571.s003.pdf]

S3 Table. Nematode-derived SVs from region 1 and their taxa and feeding types based on a BLASTN search and SILVA database.

R1_SV[a]	BLASTN data[b]							Predicted	SILVA taxonomic data[d]			
	Order	Family	Genus	Hit species used for taxonomy	E-value	% identity	Accession no.	feeding types[c]	D7	D8	D9	D10
R1_SV_3	Mononchida	Mylonchulidae	Mylonchulus	Mylonchulus hawaiiensis	0	99.72	AB361442 etc	Predator	Enoplea	Dorylaimia	Mononchida	Mylonchulus hawaiiensis
R1_SV_4	Dorylaimida	Qudsianematidae	Ecumenicus	Ecumenicus sp.*	0	99.17	MK292127	Omnivore	Enoplea	Dorylaimia	Dorylaimida	NA
R1_SV_5	Dorylaimida	Belondiridae	Dorylaimellus	Dorylaimellus virginianus*	8e-174	97.78	AY552969	Plant feeder	Enoplea	Dorylaimia	Dorylaimida	NA
R1_SV_10	Plectida	Plectidae	Plectus	Plectus sp.	0	100	MK301120 etc	Bacteria feeder	Chromadorea	NA	Araeolaimida	NA
R1_SV_12	Enoplida	Alaimidae	Alaimus	Alaimus sp. PDL-2005*	2e-179	98.88	AJ966514	Bacteria feeder	Enoplea	Enoplia	Enoplida	Alaimus sp. PDL-2005
R1_SV_14	*Triplonchida*[e]	Diphtherophoridae	Diphtherophora	Diphterophora communis*	1e-131	91.88	KY119508	Fungus feeder	Enoplea	Enoplia	Triplonchida	Diphtherophora obesus
R1_SV_22	Rhabditida	Thelastomatidae	Severianoia	Severianoia sp. 1 SVM-2019	4e-177	98.6	MN181511	Parasite	Chromadorea	NA	Ascaridida	Ambiguous_taxa
R1_SV_23	Rhabditida	Criconematidae	Mesocriconema	Mesocriconema xenoplax	0	100	MF095022 etc	Plant feeder	Chromadorea	NA	Tylenchida	NA
R1_SV_24	Rhabditida	Ungellidae	Drasico	Drasico paludigenus	6e-180	98.89	KF573585	Parasite	Chromadorea	NA	Rhabditida	Drasico nemoralis
R1_SV_27	Enoplida	Trischistomatidae	Trischistoma	Trischistoma monohystera	0	100	AJ966509	Predator	Enoplea	Enoplia	Triplonchida	NA
R1_SV_30	Chromadorida	Cyatholaimidae	Achromadora	Achromadora cf terricola JH-2004	0	99.72	AY593940	Eucaryote feeder	Chromadorea	NA	Chromadorida	Achromadora cf terricola JH-2004
R1_SV_32	Mononchida	Mylonchulidae	Mylonchulus	Mylonchulus sp.	0	100	AB361437etc	Predator	Enoplea	Dorylaimia	Mononchida	Mylonchulus hawaiiensis
R1_SV_33	Rhabditida	Cephalobidae	Acrobeloides, Chiloplacus	Acrobeloides sp., Chiloplacus propinquus	0	100	MK636581 etc	Bacteria feeder	Chromadorea	NA	Rhabditida	NA
R1_SV_34	Chromadorida	Cyatholaimidae	Achromadora	Achromadora cf terricola JH-2004	1e-177	98.88	AY593940	Eucaryote feeder	Chromadorea	NA	Chromadorida	Achromadora cf terricola JH-2004
R1_SV_36	Dorylaimida	Aporcelaimidae	Sectonema	Sectonema barbatoides	8e-174	97.78	KY119844 etc	Omnivore	Enoplea	Dorylaimia	Dorylaimida	NA
R1_SV_37	Dorylaimida	Qudsianematidae, Actinolaimidae	Ecumenicus, Paractinolaimus	Ecumenicus monohystera, Paractinolaimus macrolaimus	2e-170	97.22	KY119890 etc	Omnivore	Enoplea	Dorylaimia	Dorylaimida	Ambiguous_taxa
R1_SV_41	Triplonchida	Prismatolaimidae	Prismatolaimus	Prismatolaimus intermedius*	0	99.72	AY284729 etc	Bacteria feeder	Enoplea	Enoplia	Triplonchida	Phaseoleae environmental sample
R1_SV_43	Rhabditida	Criconematidae	Hemicriconemoides	Hemicriconemoides sp. CB 984	0	100	JX218045	Plant feeder	Chromadorea	NA	Tylenchida	NA
R1_SV_44	Dorylaimida	Belondiridae	Axonchium, Dorylaimellus	Axonchium sp. 1 WJW-2018*, Dorylaimellus virginianus*	4e-177	98.33	MG921264 etc	Plant feeder	Enoplea	Dorylaimia	Dorylaimida	Ambiguous_taxa
R1_SV_48	Rhabditida	Cephalobidae	Acrobeloides	Acrobeloides sp.*	0	99.16	MK636581 etc	Bacteria feeder	Chromadorea	NA	Rhabditida	NA
R1_SV_54	Chromadorida	Cyatholaimidae	Achromadora	Achromadora cf terricola JH-2004	1e-172	98.03	AY593940	Eucaryote feeder	Chromadorea	NA	Chromadorida	Achromadora cf terricola JH-2004
R1_SV_57	Rhabditida	Ungellidae	Drasico	Drasico paludigenus	3e-178	98.61	KF573585	Parasite	Chromadorea	NA	Rhabditida	Drasico nemoralis
R1_SV_62	Plectida	Plectidae	Plectus	Plectus minimus*	4e-177	98.33	KC206040	Bacteria feeder	Chromadorea	NA	Araeolaimida	Ambiguous_taxa
R1_SV_67	Rhabditida	Criconematidae	Mesocriconema	Mesocriconema xenoplax	0	99.72	MF095022 etc	Plant feeder	Chromadorea	NA	Tylenchida	NA
R1_SV_69	Dorylaimida	Dorylaimidae	Opisthodorylaimus	Opisthodorylaimus sylphoides*	4e-177	98.33	AY284785	Omnivore	Enoplea	Dorylaimia	Dorylaimida	Ambiguous_taxa
R1_SV_70	Enoplida	Alaimidae	Alaimus	Alaimus sp. PDL-2005*	0	99.16	AJ966514	Bacteria feeder	Enoplea	Enoplia	Enoplida	Alaimus sp. PDL-2005
R1_SV_80	Rhabditida	Pratylenchidae	Pratylenchus	Pratylenchus loosi	0	100	KY424155	Plant feeder	Chromadorea	NA	Tylenchida	Pratylenchus japonicus
R1_SV_81	Chromadorida	Cyatholaimidae	Achromadora	Achromadora ruricola*	8e-169	97.46	AY593941	Eucaryote feeder	Chromadorea	NA	Chromadorida	NA
R1_SV_84	Chromadorida	Cyatholaimidae	Achromadora	Achromadora ruricola*	4e-172	98.03	AY593941	Eucaryote feeder	Chromadorea	NA	Chromadorida	Achromadora ruricola
R1_SV_87	Rhabditida	Pratylenchidae	Pratylenchus	Pratylenchus penetrans	0	100	MN242365 etc	Plant feeder	Chromadorea	NA	Tylenchida	Pratylenchus convallariae
R1_SV_100	Dorylaimida	Belondiridae	Dorylaimellus	Dorylaimellus virginianus	4e-172	97.5	AY552969	Plant feeder	Enoplea	Dorylaimia	Dorylaimida	Dorylaimellus virginianus
R1_SV_104	Rhabditida	Tylenchidae	Basiria	Basiria sp.	0	98.9	MK639393 etc	Plant feeder	Chromadorea	NA	Tylenchida	Aglenchus agricola
R1_SV_106	Triplonchida	Prismatolaimidae	Prismatolaimus	Prismatolaimus cf. intermedius	0	100	KJ636367 etc	Bacteria feeder	Enoplea	Enoplia	Triplonchida	Phaseoleae environmental sample
R1_SV_110	*Triplonchida*	Diphtherophoridae	Diphtherophora	Diphterophora communis*	3e-123	89.66	KY119508 etc	Fungus feeder	Enoplea	Enoplia	Triplonchida	Diphtherophora obesus
R1_SV_116	Dorylaimida	Qudsianematidae	Allodorylaimus	Allodorylaimus sp. 1 WJW-2018	0	100	KY942068 etc	Omnivore	Enoplea	Dorylaimia	Dorylaimida	Ambiguous_taxa
R1_SV_119	Rhabditida	Cephalobidae	Acrobeloides	Acrobeloides sp.*	1e-172	97.77	MK636581 etc	Bacteria feeder	Chromadorea	NA	Rhabditida	metagenome
R1_SV_121	Rhabditida	Cephalobidae	Acrobeloides	Acrobeloides varius	0	99.72	MK636581 etc	Bacteria feeder	Chromadorea	NA	Rhabditida	NA
R1_SV_123	Rhabditida	Thelastomatidae	Severianoia	Severianoia sp. 1 SVM-2019	4e-177	98.6	MN181511	Parasite	Chromadorea	NA	Ascaridida	Ambiguous_taxa
R1_SV_124	Dorylaimida	Belondiridae	Axonchium, Dorylaimellus	Axonchium sp. 1 WJW-2018*, Dorylaimellus virginianus*	2e-175	98.06	MG921264 etc	Plant feeder	Enoplea	Dorylaimia	Dorylaimida	Ambiguous_taxa
R1_SV_125	Rhabditida	Aphelenchidae	Aphelenchus	Aphelenchus sp.	0	99.44	MT396111 etc	Fungus feeder	Chromadorea	NA	Tylenchida	Aphelenchus sp. JH-2004
R1_SV_132	Dorylaimida	Aporcelaimidae, Qudsianematidae, Mydonomidae	Aporcelaimellus, Ecumenicus, Dorylaimoides	Aporcelaimellus sp.*, Ecumenicus monohystera* , Dorylaimoides sp.*	0	99.44	KY119879 etc	Omnivore /Fungus feeder	Enoplea	Dorylaimia	Dorylaimida	Ambiguous_taxa
R1_SV_134	Enoplida	Alaimidae	Alaimus	Alaimus sp. PDL-2005	0	100	AJ966514	Bacteria feeder	Enoplea	Enoplia	Enoplida	Alaimus sp. PDL-2005
R1_SV_152	Rhabditida	Meloidogynidae	Meloidogyne	Meloidogyne incognita	0	100	MF177719 etc	Plant feeder	Chromadorea	NA	Tylenchida	NA
R1_SV_169	Enoplida	Alaimidae	Alaimus	Alaimus sp. PDL-2005*	1e-177	98.6	AJ966514	Bacteria feeder	Enoplea	Enoplia	Enoplida	Alaimus sp. PDL-2005
R1_SV_170	Rhabditida	Tylenchidae	Basiria	Basiria sp.	6e-180	98.62	MK639393 etc	Plant feeder	Chromadorea	NA	Tylenchida	Aglenchus agricola
R1_SV_171	Rhabditida	Rhabditidae	Distolabrellus	Distolabrellus veechi	1e-87	87.14	AF083011	Bacteria feeder	Chromadorea	NA	Rhabditida	Distolabrellus veechi
R1_SV_178	Rhabditida	Tylenchidae	Basiria	Basiria duplexa*	1e-171	97.24	KJ869382	Plant feeder	Chromadorea	NA	Tylenchida	NA
R1_SV_180	Rhabditida	Rhabditidae	Distolabrellus	Distolabrellus veechi	3e-84	86.5	AF083011	Bacteria feeder	Chromadorea	NA	Rhabditida	Distolabrellus veechi
R1_SV_184	Rhabditida	Cephalobidae	Acrobeloides	Acrobeloides sp.*	1e-172	97.77	MK636581 etc	Bacteria feeder	Chromadorea	NA	Rhabditida	NA
R1_SV_188	Dorylaimida	Qudsianematidae	Allodorylaimus	Allodorylaimus sp.*	0	94.44	KY942068 etc	Omnivore	Enoplea	Dorylaimia	Dorylaimida	Ambiguous_taxa
R1_SV_194	Chromadorida	Cyatholaimidae	Achromadora	Achromadora cf terricola JH-2004	5e-176	98.6	AY593940	Eucaryote feeder	Chromadorea	NA	Chromadorida	Achromadora cf terricola JH-2004
R1_SV_200	Rhabditida	Thelastomatidae	Severianoia	Severianoia sp. 1 SVM-2019	2e-175	98.32	MN181511	Parasite	Chromadorea	NA	Ascaridida	Ambiguous_taxa
R1_SV_205	Chromadorida	Cyatholaimidae	Achromadora	Achromadora ruricola*	4e-167	97.18	AY593941	Eucaryote feeder	Chromadorea	NA	Chromadorida	NA
R1_SV_217	Araeolaimida	Comesomatidae	Sabatieria	Sabatieria punctata*	2e-129	90.73	AY854236	NA	Chromadorea	NA	NA	NA
R1_SV_221	Triplonchida	Prismatolaimidae	Prismatolaimus	Prismatolaimus cf. dolichurus JH-2004*	0	99.44	AY284727	Bacteria feeder	Enoplea	Enoplia	Triplonchida	Ambiguous_taxa
R1_SV_230	Rhabditida	Rhabditidae	Distolabrellus	Distolabrellus veechi	5e-116	93.2	AF083011 etc	Bacteria feeder	Chromadorea	NA	Rhabditida	Distolabrellus veechi
R1_SV_231	Enoplida	Alaimidae	Alaimus	Alaimus sp. PDL-2005*	2e-174	98.04	AJ966514	Bacteria feeder	Enoplea	Enoplia	Enoplida	Alaimus sp. PDL-2005
R1_SV_233	Rhabditida	Cephalobidae	Acrobeloides	Acrobeloides thornei	0	99.16	KY119885	Bacteria feeder	Chromadorea	NA	Rhabditida	NA
R1_SV_291	Rhabditida	Cephalobidae	Cephalobus	Cephalobus cubaensis	0	100	AF202161	Bacteria feeder	Chromadorea	NA	Rhabditida	NA
R1_SV_293	Dorylaimida	Nygolaimidae	Clavicaudoides	Clavicaudoides sp. PGM-2004	2e-175	97.81	AY552967	Predator	Enoplea	Dorylaimia	Dorylaimida	Ambiguous_taxa
R1_SV_295	Chromadorida	Cyatholaimidae	Achromadora	Achromadora ruricola*	8e-174	98.31	AY593941	Eucaryote feeder	Chromadorea	NA	Chromadorida	Achromadora ruricola
R1_SV_298	Rhabditida	Tylenchidae	Miculenchus	Miculenchus salvus*	2e-155	94.96	KY119705	Plant feeder	Chromadorea	NA	Tylenchida	NA
R1_SV_304	Triplonchida	Tripylidae	Tripylella	Tripylella sp. 1031	2e-154	95.17	FJ040488	Predator	Enoplea	Enoplia	Triplonchida	NA
R1_SV_310	Rhabditida	Thelastomatidae	Severianoia	Severianoia sp. 1 SVM-2019	2e-175	98.32	MN181511	Parasite	Chromadorea	NA	Ascaridida	Ambiguous_taxa
R1_SV_312	Rhabditida	Tylenchidae	Boleodorus	Boleodorus volutus*	6e-180	98.37	FJ969117	Plant feeder	Chromadorea	NA	Tylenchida	NA
R1_SV_316	Dorylaimida	Qudsianematidae, Nordiidae	Epidorylaimus, Enchodelus	Epidorylaimus sp. 1457, Enchodelus sp. JH-2004	2e-135	91.41	FJ040478 etc	Omnivore /Predator	Enoplea	Dorylaimia	Dorylaimida	NA
R1_SV_322	Dorylaimida	Belondiridae	Axonchium, Dorylaimellus	Axonchium sp. 1 WJW-2018*, Dorylaimellus virginianus*	1e-171	97.5	MG921264 etc	Plant feeder	Enoplea	Dorylaimia	Dorylaimida	NA
R1_SV_326	Enoplida	Alaimidae	Alaimus	Alaimus sp. PDL-2005*	2e-179	98.88	AJ966514	Bacteria feeder	Enoplea	Enoplia	Enoplida	Alaimus sp. PDL-2005
R1_SV_335	Rhabditida	Criconematidae	Hemicriconemoides	Hemicriconemoides sp. CB 984	0	99.72	JX218045	Plant feeder	Chromadorea	NA	Tylenchida	NA
R1_SV_340	Rhabditida	Meloidogynidae	Meloidogyne	Meloidogyne ethiopica	2e-180	99.72	LN626921 etc	Plant feeder	Chromadorea	NA	Tylenchida	NA
R1_SV_351	Dorylaimida	Qudsianematidae, Dorylaimidae, Nordiidae	Microdorylaimus, Mesodorylaimus, Longidorella	Microdorylaimus sp.* , Mesodorylaimus cf. nigritulus*, Longidorella sp.*	2e-149	93.48	AJ966492 etc	Omnivore/Plant feeder	Enoplea	Dorylaimia	Dorylaimida	NA
R1_SV_353	Rhabditida	Meloidogynidae	Meloidogyne	Meloidogyne luci	6e-180	99.71	LN626947 etc	Plant feeder	Chromadorea	NA	Tylenchida	NA
R1_SV_371	Plectida	Plectidae	Plectus	Plectus sp.	2e-175	98.32	MK301120 etc	Bacteria feeder	Chromadorea	NA	Araeolaimida	NA
R1_SV_386	Rhabditida	Tylenchidae	Filenchus	Filenchus misellus*	3e-178	98.35	AB473564	Fungus feeder	Chromadorea	NA	Tylenchida	Ditylenchus brevicauda
R1_SV_396	Rhabditida	Tylenchidae	Filenchus	Filenchus discrepans	3e-178	99.15	KJ869311 etc	Fungus feeder	Chromadorea	NA	Tylenchida	Filenchus discrepans
R1_SV_400	Rhabditida	Hoplolaimidae	Helicotylenchus	Helicotylenchus sp.	0	100	KJ869416 etc	Plant feeder	Chromadorea	NA	Tylenchida	Helicotylenchus multicinctus
R1_SV_413	Rhabditida	Cephalobidae	Acrobeloides	Acrobeloides sp.*	2e-179	98.88	AF430537	Bacteria feeder	Chromadorea	NA	Rhabditida	NA
R1_SV_418	Rhabditida	Pratylenchidae	Pratylenchus	Pratylenchus penetrans	0	99.44	MN242365 etc	Plant feeder	Chromadorea	NA	Tylenchida	Pratylenchus convallariae
R1_SV_424	Enoplida	Trischistomatidae	Trischistoma	Trischistoma monohystera	3e-123	90.23	AJ966509	Predator	Enoplea	Enoplia	Triplonchida	Trischistoma sp. TriSSp3
R1_SV_437	Triplonchida	Trichodoridae	Paratrichodorus	Paratrichodorus porosus	0	100	MG938558 etc	Plant feeder	Enoplea	Enoplia	Triplonchida	Paratrichodorus allius
R1_SV_439	*Triplonchida*	Diphtherophoridae	Diphtherophora	Diphterophora communis*	1e-132	91.27	KY119508 etc	Fungus feeder	Enoplea	Enoplia	Triplonchida	Diphtherophora obesus
R1_SV_455	Chromadorida	Cyatholaimidae	Achromadora	Achromadora ruricola*	8e-174	98.31	AY593941	Eucaryote feeder	Chromadorea	NA	Chromadorida	NA
R1_SV_473	Rhabditida	Aphelenchoididae	Aphelenchoides	Aphelenchoides sp. 10Asp*	6e-130	91.62	KY689014	Plant feeder	Chromadorea	NA	Tylenchida	Aphelenchoides bicaudatus
R1_SV_476	Chromadorida	Cyatholaimidae	Achromadora	Achromadora ruricola	1e-157	95.75	AY593941	Eucaryote feeder	Chromadorea	NA	Chromadorida	Achromadora ruricola
R1_SV_493	Mononchida	Mylonchulidae	Mylonchulus	Mylonchulus mulveyi	0	100	AB361449	Predator	Enoplea	Dorylaimia	Mononchida	Mylonchulus mulveyi
R1_SV_507	Rhabditida	Ecphyadophoridae	Lelenchus	Lelenchus sp.	1e-146	93.58	MN542206 etc	Plant feeder	Chromadorea	NA	Tylenchida	NA
R1_SV_520	Plectida	Plectidae	Plectus	Plectus sp.	8e-174	98.04	MK301120 etc	Bacteria feeder	Chromadorea	NA	Araeolaimida	NA
R1_SV_521	Rhabditida	Ungellidae	Drasico	Drasico paludigenus	1e-161	95.82	KF573585	Parasite	Chromadorea	NA	Rhabditida	Drasico nemoralis
R1_SV_523	Chromadorida	Cyatholaimidae	Achromadora	Achromadora cf terricola JH-2004	1e-172	98.03	AY593940	Eucaryote feeder	Chromadorea	NA	Chromadorida	Achromadora cf terricola JH-2004

R1_SV_543	Chromadorida	Cyatholaimidae	Achromadora	Achromadora cf terricola JH-2004	1e-167	97.98	AY593940	Eucaryote feeder	Chromadorea	NA	Chromadorida	Achromadora cf terricola JH-2004
R1_SV_544	Rhabditida	Rhabditidae	Distolabrellus	Distolabrellus veechi	1e-82	86.17	AF083011	Bacteria feeder	Chromadorea	NA	Rhabditida	Distolabrellus veechi
R1_SV_551	Dorylaimida	Aporcelaimidae	Aporcelaimellus	Dorylaimida sp. 1 MK-2017, Aporcelaimellus sp. WW3R09	0	100	LC275858 etc	Omnivore	Enoplea	Dorylaimia	Dorylaimida	NA
R1_SV_556	Rhabditida	Aphelenchoididae	Aphelenchoides	Aphelenchoides sp.*	1e-131	91.95	KY689014 etc	Plant feeder	Chromadorea	NA	Tylenchida	Aphelenchoides bicaudatus
R1_SV_562	Mononchida	Mylonchulidae	Mylonchulus	Mylonchulus sp.	2e-170	96.97	AB361437 etc	Predator	Enoplea	Dorylaimia	Mononchida	Mylonchulus hawaiiensis
R1_SV_578	Rhabditida	Tylenchidae	Filenchus	Ottolenchus longiurus	3e-163	96.36	KJ869337 etc	Fungus feeder	Chromadorea	NA	Tylenchida	Filenchus discrepans
R1_SV_594	Mononchida	Mylonchulidae	Mylonchulus	Mylonchulus sp.	1e-166	96.42	AB361437 etc	Predator	Enoplea	Dorylaimia	Mononchida	Mylonchulus hawaiiensis

[a]Regional nematode SVs in region 1 were identified from four data sets (amplicons from field, copse, and house garden samples and from the field sample amplified by 2-step PCR). The italic SVs were only detected in the 2-step PCR experiment.

[b]The top hit of species, genus, family, and order, e-value and sequence cover rate from BLASTN search were indicated. Top hit sequences without taxonomic data such as environmental samples were omitted and the second-closest species to the query SV sequences are shown instead with asterisks to use taxonomic assignment of the SVs.

[c]Feeding types of the SVs were predicted based on those of the closest species, based on the work performed by Yeates and the functional guildes for each feeding types at the Nemaplex home page (http://nemaplex.ucdavis.edu/Uppermnus/topmnu.htm). Parasist was predicted by previous publications.

[d]The taxonomic ranks of D7-D10 corresponding to the SVs are indicated. NA: Not assigned.

[e]*Diphterophora communis* was assigned to Triolonchida as described by Kenmotsu et al. [28].
