# Supplementary material for: Distinct community structures of soil nematodes from three ecologically different sites revealed by high-throughput amplicon sequencing of four 18S ribosomal RNA gene regions
Source: PLoS One. 2021 Apr 15;16(4):e0249571. doi: 10.1371/journal.pone.0249571 (PMC8049254; doi:10.1371/journal.pone.0249571)
Supplement: S4 Table — (PDF) [file pone.0249571.s004.pdf]

S4 Table. Nematode-derived SVs from region 2 and their taxa and feeding types based on a BLASTN search and SILVA database.

R2_SV	BLASTN data							Predicted feeding types	SILVA taxonomic data			
	Order	Family	Genus	Hit species used for taxonomy	E-value	% identity	Accession no.		D7	D8	D9	D10
R2_SV_1	Rhabditida	Aphelenchidae	Aphelenchus	Aphelenchus sp.	3e-178	100	LC275870 etc	Fungus feeder	Chromadorea	NA	Tylenchida	Aphelenchus sp. JH-2004
R2_SV_3	Mononchida	Mylonchulidae	Mylonchulus	Mylonchulus sp.	2e-180	100	MN082300 etc	Predator	Enoplea	Dorylaimia	Mononchida	NA
R2_SV_5	Dorylaimida	Dorylaimidae	Mesodorylaimus	Mesodorylaimus sp.	6e-180	99.71	MG921252 etc	Omnivore	Enoplea	Dorylaimia	Dorylaimida	Nematoda environmental sample
R2_SV_10	Dorylaimida	Belondiridae	Dorylaimellus	Dorylaimellus virginianus*	6e-175	98.86	AY552969	Plant feeder	Enoplea	Dorylaimia	Dorylaimida	Tylencholaimus mirabilis
R2_SV_12	Rhabditida	Travassosinematidae	Travassosinema	Travassosinema sp.	1e-171	98.29	LC214832	Parasite	Chromadorea	NA	NA	NA
R2_SV_15	Enoplida	Alaimidae	Alaimus	Alaimus sp. HL	0	100	MK809264	Bacteria feeder	Enoplea	Enoplia	Enoplida	Alaimus sp. PDL-2005
R2_SV_17	Plectida	Plectidae	Plectus	Plectus sp.	2e-180	100	MN082338 etc	Bacteria feeder	Chromadorea	NA	NA	NA
R2_SV_18	Rhabditida	Ungellidae	Drasico	Drasico nemoralis	1e-157	96.24	KF573586	Parasite	Chromadorea	NA	Rhabditida	Drasico nemoralis
R2_SV_19	Rhabditida	Criconematidae	Mesocriconema	Mesocriconema xenoplax	6e-180	100	MF095022 etc	Plant feeder	Chromadorea	NA	Tylenchida	Mesocriconema xenoplax
R2_SV_21	Rhabditida	Cephalobidae	Acrobeloides	Acrobeloides varius	2e-179	100	MK636581 etc	Bacteria feeder	Chromadorea	NA	Rhabditida	Acrobeles ciliatus
R2_SV_24	Rhabditida	Pratylenchidae	Pratylenchus	Pratylenchus loosi	0	99.72	LR215657	Plant feeder	Chromadorea	NA	Tylenchida	NA
R2_SV_25	Mononchida	Mylonchulidae	Mylonchulus	Mylonchulus brachyuris	2e-180	100	AB361437 etc	Predator	Enoplea	Dorylaimia	Mononchida	NA
R2_SV_26	Rhabditida	Tylenchidae	Basiria	Basiria sp.	4e-162	96.84	MK639393 etc	Plant feeder	Chromadorea	NA	Tylenchida	Basiria duplexa
R2_SV_30	Dorylaimida	Actinolaimidae	Paractinolaimus	Paractinolaimus macrolaimus*	1e-176	99.14	KY119864	Predator	Enoplea	Dorylaimia	Dorylaimida	Ambiguous_taxa
R2_SV_31	Triplonchida	Diphtherophoridae	Diphtherophora	Diphtherophora obesus*	5e-121	89.91	KY119878 etc	Fungus feeder	Enoplea	Enoplia	Triplonchida	NA
R2_SV_35	Dorylaimida	Aporcelaimidae	Sectonema, Aporcelaimellus	Sectonema sp.*, Aporcelaimellus sp. F2*	6e-180	99.71	KY119752 etc	Omnivore	Enoplea	Dorylaimia	Dorylaimida	Poduridae environmental sample
R2_SV_36	Plectida	Plectidae	Ceratoplectus	Ceratoplectus sp.*	4e-177	99.43	MN082268 etc	Bacteria feeder	Chromadorea	NA	Araeolaimida	Plectus sp.
R2_SV_38	Chromadorida	Cyatholaimidae	Achromadora	Achromadora cf terricola JH-2004	0	100	AY593940	Eucaryote feeder	Chromadorea	NA	Chromadorida	Achromadora cf terricola JH-2004
R2_SV_41	Dorylaimida	Leptonchidae	Proleptonchus	Proleptonchus weischeri	3e-173	98.57	KJ636399	Fungus feeder	Enoplea	Dorylaimia	Dorylaimida	NA
R2_SV_42	Enoplida	Trischistomatidae	Trischistoma	Trischistoma monohystera	0	100	AJ966509 etc	Predator	Enoplea	Enoplia	Triplonchida	NA
R2_SV_47	Rhabditida	Travassosinematidae	Travassosinema	Travassosinema sp. Nago	1e-171	98.29	LC214829	Parasite	Chromadorea	NA	Oxyurida	NA
R2_SV_48	Rhabditida	Cephalobidae	Heterocephalobus	Heterocephalobus elongatus	1e-177	99.71	EU040128	Bacteria feeder	Chromadorea	NA	Rhabditida	metagenome
R2_SV_52	Rhabditida	Aphelenchidae	Aphelenchus	Aphelenchus sp.	6e-175	99.42	JX421761 etc	Fungus feeder	Chromadorea	NA	Tylenchida	Aphelenchus sp. JH-2004
R2_SV_53	Triplonchida	Prismatolaimidae	Prismatolaimus	Prismatolaimus intermedius	6e-180	100	EU040133	Bacteria feeder	Enoplea	Enoplia	Triplonchida	Schizomidae environmental sample
R2_SV_54	Triplonchida	Prismatolaimidae	Prismatolaimus	Prismatolaimus cf. intermedius	3e-178	99.71	KJ636367 etc	Bacteria feeder	Enoplea	Enoplia	Triplonchida	Schizomidae environmental sample
R2_SV_56	Rhabditida	Criconematidae, Hemicycliophoridae	Criconemoides, Hemicriconemoides, Hemicycliophora	Criconemoides parainformis, Hemicriconemoides sp., Hemicycliophora conida	2e-179	100	MN738711 etc	Plant feeder	Chromadorea	NA	Tylenchida	Ogma sp. WY-2013
R2_SV_63	Dorylaimida	Qudsianematidae	Allodorylaimus	Allodorylaimus sp.	0	100	KY942068 etc	Omnivore	Enoplea	Dorylaimia	Dorylaimida	Ambiguous_taxa
R2_SV_72	Rhabditida	Rhabditidae	Diploscapter	Diploscapter sp.	4e-176	100	MN082283 etc	Bacteria feeder	Chromadorea	NA	Rhabditida	Protorhabditis sp. JB122
R2_SV_89	Desmodorida	Microlaimidae	Prodesmodora	Prodesmodora circulata	0	100	AY284721	Bacteria feeder	Chromadorea	NA	Chromadorida	Prodesmodora circulata
R2_SV_90	Rhabditida	Rhabditidae	Diploscapter	Diploscapter sp.	1e-172	99.41	MN082283 etc	Bacteria feeder	Chromadorea	NA	Rhabditida	Protorhabditis sp. JB122
R2_SV_95	Rhabditida	Tylenchidae	Basiria	Basiria duplexa*	8e-169	97.99	KJ869382 etc	Plant feeder	Chromadorea	NA	Tylenchida	NA
R2_SV_102	Dorylaimida	Tylencholaimidae	Tylencholaimus	Tylencholaimus mirabilis*	3e-168	97.72	EF207253	Fungus feeder	Enoplea	Dorylaimia	Dorylaimida	NA
R2_SV_103	Dorylaimida	Qudsianematidae	Microdorylaimus	Microdorylaimus miser*	1e-176	99.14	AY284804	Omnivore	Enoplea	Dorylaimia	Dorylaimida	Ambiguous_taxa
R2_SV_105	Rhabditida	Aphelenchidae	Aphelenchus	Aphelenchus sp.	1e-176	99.71	LC275870 etc	Fungus feeder	Chromadorea	NA	Tylenchida	Aphelenchus sp. JH-2004
R2_SV_110	Chromadorida	Cyatholaimidae	Achromadora	Achromadora cf terricola JH-2004	1e-167	97.71	AY593940	Eucaryote feeder	Chromadorea	NA	Chromadorida	Achromadora cf terricola JH-2004
R2_SV_114	Rhabditida	Homungellidae	Perodira	Perodira minuta	2e-164	97.41	EU287478	Parasite	Chromadorea	NA	Rhabditida	NA
R2_SV_116	Triplonchida	Diphtherophoridae	Diphtherophora	Diphtherophora obesus*	2e-119	89.63	AY552968	Fungus feeder	Enoplea	Enoplia	Triplonchida	NA
R2_SV_120	Rhabditida	Pratylenchidae	Pratylenchus	Pratylenchus penetrans	3e-178	99.43	MH983023	Plant feeder	Chromadorea	NA	Tylenchida	Pratylenchus penetrans
R2_SV_127	Rhabditida	Pratylenchidae	Pratylenchus	Pratylenchus penetrans	3e-178	99.43	MH983023	Plant feeder	Chromadorea	NA	Tylenchida	Pratylenchus penetrans
R2_SV_128	Rhabditida	Pratylenchidae	Pratylenchus	Pratylenchus penetrans	1e-176	99.15	MH983023	Plant feeder	Chromadorea	NA	Tylenchida	Pratylenchus penetrans
R2_SV_129	Rhabditida	Pratylenchidae	Pratylenchus	Pratylenchus penetrans	3e-178	99.43	EU669926	Plant feeder	Chromadorea	NA	Tylenchida	Pratylenchus penetrans
R2_SV_131	Dorylaimida	Belondiridae	Dorylaimellus	Dorylaimellus virginianus*	3e-173	98.57	AY552969	Plant feeder	Enoplea	Dorylaimia	Dorylaimida	Tylencholaimus mirabilis
R2_SV_132	Rhabditida	Travassosinematidae	Travassosinema	Travassosinema sp. Ishigaki	6e-170	98	LC214832	Parasite	Chromadorea	NA	NA	NA
R2_SV_134	Rhabditida	Meloidogynidae	Meloidogyne	Meloidogyne incognita	8e-179	100	MF177719	Plant feeder	Chromadorea	NA	Tylenchida	NA
R2_SV_135	Rhabditida	Homungellidae	Perodira	Perodira minuta	5e-161	96.83	EU287478	Parasite	Chromadorea	NA	Rhabditida	NA
R2_SV_139	Enoplida	Alaimidae	Alaimus	Alaimus sp. PDL-2005	6e-170	98	AJ966514	Bacteria feeder	Enoplea	Enoplia	Enoplida	Alaimus sp. PDL-2005
R2_SV_140	Rhabditida	Aphelenchidae	Aphelenchus	Aphelenchus avenae	3e-178	100	MH136627 etc	Fungus feeder	Chromadorea	NA	Tylenchida	Aphelenchus avenae
R2_SV_142	Dorylaimida	Tylencholaimidae	Tylencholaimus	Tylencholaimus mirabilis*	1e-166	97.44	EF207253	Fungus feeder	Enoplea	Dorylaimia	Dorylaimida	NA
R2_SV_145	Rhabditida	Pratylenchidae	Pratylenchus	Pratylenchus penetrans	2e-164	97.14	AB905306 etc	Plant feeder	Chromadorea	NA	Tylenchida	Pratylenchus penetrans
R2_SV_147	Chromadorida	Cyatholaimidae	Achromadora	Achromadora sp. JH-2004*	1e-167	97.43	AY284718	Eucaryote feeder	Chromadorea	NA	Chromadorida	Achromadora sp. JH-2004
R2_SV_150	Rhabditida	Tylenchidae	Boleodorus	Boleodorus thylactus*	6e-175	98.86	MK639397 etc	Plant feeder	Chromadorea	NA	Tylenchida	Boleodorus thylactus
R2_SV_164	Chromadorida	Cyatholaimidae	Achromadora	Achromadora cf terricola JH-2004	2e-169	98	AY593940	Eucaryote feeder	Chromadorea	NA	Chromadorida	Achromadora cf terricola JH-2004
R2_SV_168	Dorylaimida	Belondiridae	Dorylaimellus	Dorylaimellus virginianus*	6e-170	98	AY552969	Plant feeder	Enoplea	Dorylaimia	Dorylaimida	Tylencholaimus mirabilis
R2_SV_176	Rhabditida	Cephalobidae	Acrobeloides	Acrobeloides thornei	1e-172	98.84	KY119635	Bacteria feeder	Chromadorea	NA	Rhabditida	NA
R2_SV_179	Rhabditida	Tylenchidae	Psilenchus	Psilenchus hilarulus	8e-179	100	MK639403 etc	Plant feeder	Chromadorea	NA	Tylenchida	Psilenchus sp. CA12
R2_SV_180	Rhabditida	Travassosinematidae	Travassosinema	Travassosinema sp. Ishigaki	6e-170	98	LC214832	Parasite	Chromadorea	NA	NA	NA
R2_SV_186	Rhabditida	Tylenchidae	Filenchus	Filenchus discrepans	2e-134	92.31	KJ869311 etc	Fungus feeder	Chromadorea	NA	Tylenchida	Filenchus discrepans
R2_SV_190	Rhabditida	Cephalobidae	Acrobeloides	Acrobeloides thornei	8e-174	99.13	KY119885	Bacteria feeder	Chromadorea	NA	Rhabditida	NA
R2_SV_198	Rhabditida	Hoplolaimidae	Helicotylenchus	Helicotylenchus dihystera	0	100	MK796435 etc	Plant feeder	Chromadorea	NA	Tylenchida	Helicotylenchus digitiformis
R2_SV_199	Plectida	Plectidae	Plectus	Plectus sp. Rimu	2e-180	100	JX676807	Bacteria feeder	Chromadorea	NA	Araeolaimida	NA
R2_SV_200	Dorylaimida	Mydonomidae	Dorylaimoides	Dorylaimoides sp.	6e-175	98.86	KU662325	Fungus feeder	Enoplea	Dorylaimia	Dorylaimida	Nematoda environmental sample
R2_SV_202	Rhabditida	Pratylenchidae	Pratylenchus	Pratylenchus loosi	6e-180	99.43	LR215657	Plant feeder	Chromadorea	NA	Tylenchida	Pratylenchus japonicus
R2_SV_205	Rhabditida	Criconematidae	Mesocriconema	Mesocriconema xenoplax	3e-178	99.71	MF095022 etc	Plant feeder	Chromadorea	NA	Tylenchida	Mesocriconema xenoplax
R2_SV_208	Rhabditida	Meloidogynidae	Meloidogyne	Meloidogyne sp.	8e-179	100	AB905319 etc	Plant feeder	Chromadorea	NA	Tylenchida	NA
R2_SV_209	Rhabditida	Pratylenchidae	Pratylenchus	Pratylenchus penetrans	3e-173	98.57	KJ934156 etc	Plant feeder	Chromadorea	NA	Tylenchida	Pratylenchus penetrans
R2_SV_214	Rhabditida	Pratylenchidae	Pratylenchus	Pratylenchus penetrans	3e-178	99.43	AB905304	Plant feeder	Chromadorea	NA	Tylenchida	Pratylenchus penetrans
R2_SV_215	Rhabditida	Cephalobidae	Cephalobus	Cephalobus sp. 1 GVDU-2019	6e-180	99.71	MN082264	Bacteria feeder	Chromadorea	NA	Rhabditida	Cephalobus cubaensis
R2_SV_217	Rhabditida	Tylenchidae	Filenchus	Filenchus misellus*	8e-174	98.58	KJ869308	Fungus feeder	Chromadorea	NA	Tylenchida	Filenchus misellus
R2_SV_222	Rhabditida	Travassosinematidae	Travassosinema	Travassosinema sp. Ishigaki	1e-166	97.43	LC214832	Parasite	Chromadorea	NA	NA	NA
R2_SV_237	Rhabditida	Pratylenchidae	Pratylenchus	Pratylenchus penetrans	0	100	AB905306	Plant feeder	Chromadorea	NA	Tylenchida	Pratylenchus penetrans
R2_SV_238	Dorylaimida	Mydonomidae	Dorylaimoides	Dorylaimoides sp. WJW-2016	3e-178	99.43	KU662325	Fungus feeder	Enoplea	Dorylaimia	Dorylaimida	Nematoda environmental sample
R2_SV_243	Rhabditida	Pratylenchidae	Pratylenchus	Pratylenchus penetrans	3e-173	98.58	AB905306	Plant feeder	Chromadorea	NA	Tylenchida	Pratylenchus penetrans
R2_SV_246	Rhabditida	Travassosinematidae	Travassosinema	Travassosinema sp. Ishigaki	1e-166	97.43	LC214832	Parasite	Chromadorea	NA	NA	NA
R2_SV_247	Rhabditida	Pratylenchidae	Pratylenchus	Pratylenchus penetrans	3e-178	99.43	EU669926	Plant feeder	Chromadorea	NA	Tylenchida	Pratylenchus penetrans
R2_SV_253	Rhabditida	Pratylenchidae	Pratylenchus	Pratylenchus penetrans	1e-176	99.15	MH983023	Plant feeder	Chromadorea	NA	Tylenchida	Pratylenchus penetrans
R2_SV_259	Rhabditida	Pratylenchidae	Pratylenchus	Pratylenchus penetrans	1e-176	99.15	MH983023	Plant feeder	Chromadorea	NA	Tylenchida	Pratylenchus penetrans
R2_SV_260	Enoplida	Alaimidae	Alaimus	Alaimus sp. HL	1e-176	99.14	MK809264	Bacteria feeder	Enoplea	Enoplia	Enoplida	Alaimus sp. PDL-2005
R2_SV_262	Desmodorida	Microlaimidae	Prodesmodora	Prodesmodora circulata*	3e-158	96	AY284721 etc	Bacteria feeder	Chromadorea	NA	Chromadorida	NA
R2_SV_265	Rhabditida	Pratylenchidae	Pratylenchus	Pratylenchus penetrans strain PratPen2	6e-175	98.86	EU669926	Plant feeder	Chromadorea	NA	Tylenchida	Pratylenchus penetrans
R2_SV_270	Rhabditida	Pratylenchidae	Pratylenchus	Pratylenchus penetrans strain PratPen2	1e-176	99.14	EU669926	Plant feeder	Chromadorea	NA	Tylenchida	Pratylenchus penetrans
R2_SV_272	*Rhabditida*	Cephalobidae	Cephalobus	Cephalobus cubaensis	6e-180	99.71	AF202161 etc	Bacteria feeder	Chromadorea	NA	Rhabditida	Cephalobus cubaensis
R2_SV_273	Rhabditida	Pratylenchidae	Pratylenchus	Pratylenchus penetrans	6e-175	98.86	MH983023 etc	Plant feeder	Chromadorea	NA	Tylenchida	Pratylenchus penetrans
R2_SV_275	Triplonchida	Tripylidae	Tripylella	Tripylella sp. 1031	4e-162	96.85	FJ040488	Predator	Enoplea	Enoplia	Triplonchida	Tripylella sp. 1031
R2_SV_279	Rhabditida	Pratylenchidae	Pratylenchus	Pratylenchus penetrans isolate ILVO-Pp	6e-175	98.86	MH983023	Plant feeder	Chromadorea	NA	Tylenchida	Pratylenchus penetrans
R2_SV_281	Rhabditida	Cephalobidae	Acrobeloides	Acrobeloides thornei isolate I17_SMIRACONTROL33	2e-170	98.55	KY119885	Bacteria feeder	Chromadorea	NA	Rhabditida	NA
R2_SV_282	Rhabditida	Travassosinematidae	Travassosinema	Travassosinema sp. Ishigaki	3e-168	97.71	LC214832	Parasite	Chromadorea	NA	NA	NA
R2_SV_288	Enoplida	Alaimidae	Alaimus	Alaimus sp. HL	1e-176	99.14	MK809264	Bacteria feeder	Enoplea	Enoplia	Enoplida	Alaimus sp. PDL-2005

R2_SV_291	Rhabditida	Tylenchidae	Boleodorus	Boleodorus thylactus	1e-171	98.29	MK639397 etc	Plant feeder	Chromadorea	NA	Tylenchida	Boleodorus thylactus
R2_SV_296	Mononchida	Mylonchulidae	Mylonchulus	Mylonchulus mulveyi	8e-179	99.71	AB361449 etc	Predator	Enoplea	Dorylaimia	Mononchida	Mylonchulus mulveyi
R2_SV_299	Rhabditida	Tylenchidae	Basiria	Basiria gracilis	0	100	MK639395 etc	Plant feeder	Chromadorea	NA	Tylenchida	Basiria gracilis
R2_SV_300	Triplonchida	Prismatolaimidae	Prismatolaimus	Prismatolaimus cf. dolichurus JH-2004*	3e-177	99.71	AY284727 etc	Bacteria feeder	Enoplea	Enoplia	Triplonchida	Ambiguous_taxa
R2_SV_301	Rhabditida	Pratylenchidae	Pratylenchus	Pratylenchus penetrans strain PratPen2	6e-175	98.86	EU669926	Plant feeder	Chromadorea	NA	Tylenchida	Pratylenchus penetrans
R2_SV_303	Rhabditida	Criconematidae	Mesocriconema	Mesocriconema xenoplax	1e-176	99.42	MF095022 etc	Plant feeder	Chromadorea	NA	Tylenchida	Mesocriconema xenoplax
R2_SV_304	Rhabditida	Pratylenchidae	Pratylenchus	Pratylenchus penetrans	1e-171	98.29	MH983023 etc	Plant feeder	Chromadorea	NA	Tylenchida	Pratylenchus penetrans
R2_SV_315	Rhabditida	Pratylenchidae	Pratylenchus	Pratylenchus penetrans	6e-175	98.86	KJ934156 etc	Plant feeder	Chromadorea	NA	Tylenchida	Pratylenchus penetrans
R2_SV_317	Dorylaimida	Nygolaimidae	Clavicaudoides, Aquatides	Clavicaudoides sp. PGM-2004, Aquatides christei isolate Konza IIAC-02	1e-177	98.88	AY552967, AY552963	Predator	Enoplea	Dorylaimia	Dorylaimida	Clavicaudoides clavicaudatus
R2_SV_321	Rhabditida	Meloidogynidae	Meloidogyne	Meloidogyne ethiopica, Meloidogyne incognita	8e-179	100	LN626932 etc	Plant feeder	Chromadorea	NA	Tylenchida	NA
R2_SV_324	Desmodorida	Microlaimidae	Prodesmodora	Prodesmodora circulata isolate PrDeCir3	1e-176	99.14	AY284721	Bacteria feeder	Chromadorea	NA	Chromadorida	Achromadora sp. JH-2004
R2_SV_327	Rhabditida	Tylenchidae	Psilenchus	Psilenchus sp.	4e-177	99.71	MK639403 etc	Plant feeder	Chromadorea	NA	Tylenchida	Psilenchus sp. CA12
R2_SV_329	Rhabditida	Tylenchidae	Filenchus	Filenchus misellus*	1e-166	97.44	KJ869308	Fungus feeder	Chromadorea	NA	Tylenchida	Filenchus misellus
R2_SV_334	Rhabditida	Pratylenchidae	Pratylenchus	Pratylenchus loosi	1e-177	99.15	LR215657	Plant feeder	Chromadorea	NA	Tylenchida	NA
R2_SV_336	Rhabditida	Tylenchidae	Miculenchus	Miculenchus salmae*	2e-85	83.91	MF599079	Plant feeder	Chromadorea	NA	Tylenchida	NA
R2_SV_337	Rhabditida	Pratylenchidae	Pratylenchus	Pratylenchus penetrans	6e-175	98.86	KJ934156 etc	Plant feeder	Chromadorea	NA	Tylenchida	Pratylenchus penetrans
R2_SV_338	Dorylaimida	Dorylaimidae, Actinolaimidae, Qudsianematidae	Mesodorylaimus, Paractinolaimus, Labronema	Mesodorylaimus sp., Paractinolaimus macrolaimus, Labronema vulvapapillatum	6e-165	97.14	MG921252 etc	Omnivore	Enoplea	Dorylaimia	Dorylaimida	NA
R2_SV_343	Rhabditida	Tylenchidae	Filenchus	Ottolenchus longiurus strain FileLon2	1e-136	92.59	KJ869337	Fungus feeder	Chromadorea	NA	Tylenchida	Filenchus longiurus
R2_SV_349	Rhabditida	Pratylenchidae	Pratylenchus	Pratylenchus penetrans	3e-178	99.43	AB905304	Plant feeder	Chromadorea	NA	Tylenchida	Pratylenchus penetrans
R2_SV_351	Dorylaimida	Aporcelaimidae	Sectonema, Aporcelaimellus	Sectonema sp.*, Aporcelaimellus sp. F2*	6e-175	98.86	KY119752 etc	Omnivore	Enoplea	Dorylaimia	Dorylaimida	Poduridae environmental sample
R2_SV_355	Mononchida	Mylonchulidae	Mylonchulus	Mylonchulus brachyuris	4e-177	99.43	AB361437 etc	Predator	Enoplea	Dorylaimia	Mononchida	NA
R2_SV_362	Desmodorida, Chromadorida	Microlaimidae, Cyatholaimidae	Prodesmodora, Achromadora	Prodesmodora circulata*, Achromadora sp. JH-2004*	6e-160	96.29	AY284721 etc	Bacteria feeder/Eeukayote feeder	Chromadorea	NA	Chromadorida	Achromadora sp. JH-2004
R2_SV_363	Triplonchida	Diphtherophoridae	Diphtherophora	Diphtherophora obesus*	2e-124	90.49	AY552968	Fungus feeder	Enoplea	Enoplia	Triplonchida	NA
R2_SV_369	Rhabditida	Pratylenchidae	Pratylenchus	Pratylenchus penetrans strain PratPen2	3e-173	98.57	EU669926	Plant feeder	Chromadorea	NA	Tylenchida	Pratylenchus penetrans
R2_SV_371	Rhabditida	Tylenchidae	Basiria	Basiria gracilis	3e-178	99.15	MK639395 etc	Plant feeder	Chromadorea	NA	Tylenchida	Basiria gracilis
R2_SV_386	Desmodorida	Microlaimidae	Prodesmodora	Prodesmodora circulata isolate PrDeCir3	3e-178	99.43	AY284721	Bacteria feeder	Chromadorea	NA	Chromadorida	Prodesmodora circulata
R2_SV_390	Triplonchida	Prismatolaimidae	Prismatolaimus	Prismatolaimus cf. intermedius *	6e-165	97.41	KJ636367 etc	Bacteria feeder	Enoplea	Enoplia	Triplonchida	Phaseoleae environmental sample
R2_SV_399	Rhabditida	Aphelenchidae	Aphelenchus	Aphelenchus avenae	1e-176	99.71	MH136627 etc	Fungus feeder	Chromadorea	NA	Tylenchida	Aphelenchus avenae
R2_SV_401	Triplonchida	Diphtherophoridae	Diphtherophora	Diphtherophora obesus*	4e-127	91.04	KY119878	Fungus feeder	Enoplea	Enoplia	Triplonchida	NA
R2_SV_404	Rhabditida	Pratylenchidae	Pratylenchus	Pratylenchus penetrans	0	100	AB905305	Plant feeder	Chromadorea	NA	Tylenchida	Pratylenchus penetrans
R2_SV_410	Rhabditida	Pratylenchidae	Pratylenchus	Pratylenchus penetrans	6e-175	98.86	MH983023 etc	Plant feeder	Chromadorea	NA	Tylenchida	Pratylenchus penetrans
R2_SV_411	Rhabditida	Pratylenchidae	Pratylenchus	Pratylenchus penetrans	1e-176	99.15	MH983023 etc	Plant feeder	Chromadorea	NA	Tylenchida	Pratylenchus penetrans
R2_SV_414	Rhabditida	Pratylenchidae	Pratylenchus	Pratylenchus penetrans strain PratPen2	6e-175	98.86	EU669926	Plant feeder	Chromadorea	NA	Tylenchida	Pratylenchus penetrans
R2_SV_415	Rhabditida	Pratylenchidae	Pratylenchus	Pratylenchus penetrans	3e-173	98.58	MH983023 etc	Plant feeder	Chromadorea	NA	Tylenchida	Pratylenchus penetrans
R2_SV_418	Triplonchida	Prismatolaimidae	Prismatolaimus	Prismatolaimus cf. intermedius*	1e-166	97.69	KJ636367 etc	Bacteria feeder	Enoplea	Enoplia	Triplonchida	NA
R2_SV_426	Enoplida	Trischistomatidae	Trischistoma	Trischistoma monohystera	1e-122	90.23	AJ966509 etc	Predator	Enoplea	Enoplia	Triplonchida	NA
R2_SV_427	Dorylaimida	Aporcelaimidae	Sectonema, Aporcelaimellus	Sectonema sp.*, Aporcelaimellus sp. F2*	1e-176	99.14	KY119752 etc	Omnivore	Enoplea	Dorylaimia	Dorylaimida	Poduridae environmental sample
R2_SV_430	Chromadorida	Cyatholaimidae	Achromadora	Achromadora sp. JH-2004*	2e-164	96.87	AY284718	Eucaryote feeder	Chromadorea	NA	Chromadorida	Achromadora sp. JH-2004
R2_SV_433	Rhabditida	Pratylenchidae	Pratylenchus	Pratylenchus penetrans strain PratPen2	6e-175	98.86	EU669926	Plant feeder	Chromadorea	NA	Tylenchida	Pratylenchus penetrans
R2_SV_443	Rhabditida	Meloidogynidae	Meloidogyne	Meloidogyne incognita	2e-175	99.42	MF177719 etc	Plant feeder	Chromadorea	NA	Tylenchida	NA
R2_SV_448	Rhabditida	Tylenchidae	Basiria	Basiria gracilis isolate CA1	1e-166	96.93	EU130839	Plant feeder	Chromadorea	NA	Tylenchida	Basiria gracilis
R2_SV_450	Rhabditida	Tylenchidae	Miculenchus	Miculenchus salvus*	2e-129	91.43	KY119705	Plant feeder	Chromadorea	NA	Tylenchida	NA
R2_SV_452	Rhabditida	Tylenchidae	Filenchus	Filenchus discrepans strain FileDis2	5e-141	93.43	KJ869311	Fungus feeder	Chromadorea	NA	Tylenchida	Filenchus discrepans
R2_SV_461	Rhabditida	Aphelenchidae	Aphelenchus	Aphelenchus avenae	6e-175	99.42	MH136627 etc	Fungus feeder	Chromadorea	NA	Tylenchida	Aphelenchus avenae
R2_SV_470	Triplonchida	Prismatolaimidae	Prismatolaimus	Prismatolaimus cf. intermedius	2e-119	89.91	KJ636367 etc	Bacteria feeder	Enoplea	Enoplia	Triplonchida	Schizomidae environmental sample
R2_SV_471	Chromadorida	Cyatholaimidae	Achromadora	Achromadora sp. JH-2004 isolate AchrSp2	5e-166	97.14	AY284718	Eucaryote feeder	Chromadorea	NA	Chromadorida	Achromadora sp. JH-2004
R2_SV_476	Plectida	Plectidae	Plectus	Plectus sp.	1e-157	100	MN082338 etc	Bacteria feeder	Chromadorea	NA	NA	NA
R2_SV_482	Enoplida	Trischistomatidae	Trischistoma	Trischistoma sp.	1e-177	99.15	AJ966509 etc	Predator	Enoplea	Enoplia	Triplonchida	Trischistoma sp. TriSSp3
R2_SV_483	Rhabditida	Tylenchidae	Filenchus	Filenchus discrepans strain FileDis2	2e-144	93.97	KJ869311	Fungus feeder	Chromadorea	NA	Tylenchida	Filenchus discrepans
R2_SV_490	Dorylaimida	Qudsianematidae, Dorylaimidae	Epidorylaimus, Prodorylaimus	Epidorylaimus sp., Prodorylaimus mas	3e-148	94.3	FJ040478 etc	Omnivore	Enoplea	Dorylaimia	Dorylaimida	Epidorylaimus lugdunensis
R2_SV_499	Dorylaimida	Qudsianematidae, Dorylaimidae	Epidorylaimus, Prodorylaimus	Epidorylaimus sp., Prodorylaimus mas	3e-153	95.16	JN049661 etc	Omnivore	Enoplea	Dorylaimia	Dorylaimida	Epidorylaimus lugdunensis
R2_SV_500	Rhabditida	Pratylenchidae	Pratylenchus	Pratylenchus penetrans isolate ILVO-Pp	1e-176	99.15	MH983023	Plant feeder	Chromadorea	NA	Tylenchida	Pratylenchus penetrans
R2_SV_505	Triplonchida	Diphtherophoridae	Diphtherophora	Diphtherophora obesus*	2e-125	90.75	KY119878	Fungus feeder	Enoplea	Enoplia	Triplonchida	NA
R2_SV_507	Enoplida	Trischistomatidae	Trischistoma	Trischistoma sp.	1e-131	91.48	AJ966509 etc	Predator	Enoplea	Enoplia	Triplonchida	NA
R2_SV_511	Chromadorida	Cyatholaimidae	Achromadora	Achromadora sp. JH-2004 isolate AchrSp2	2e-169	97.71	AY284718	Eucaryote feeder	Chromadorea	NA	Chromadorida	Achromadora sp. JH-2004
R2_SV_517	Rhabditida	Aphelenchoididae	Aphelenchoides	Aphelenchoides sp. RH-2018	6e-170	98.28	MF070486	Plant feeder	Chromadorea	NA	Tylenchida	metagenome
R2_SV_519	Rhabditida	Pratylenchidae	Pratylenchus	Pratylenchus penetrans isolate ILVO-Pp	3e-173	98.58	MH983023	Plant feeder	Chromadorea	NA	Tylenchida	Pratylenchus penetrans
R2_SV_531	Desmodorida, Chromadorida	Microlaimidae, Cyatholaimidae	Prodesmodora, Achromadora	Prodesmodora circulata*, Achromadora sp. JH-2004*	6e-160	96.29	AY284721 etc	Bacteria feeder/Eeukayote feeder	Chromadorea	NA	Chromadorida	Achromadora sp. JH-2004
R2_SV_549	Enoplida	Trischistomatidae	Trischistoma	Trischistoma sp.	8e-174	98.58	KR492034 etc	Predator	Enoplea	Enoplia	Triplonchida	NA
R2_SV_551	Dorylaimida	Qudsianematidae	Microdorylaimus	Microdorylaimus miser isolate MicDMis	6e-165	97.14	AY284804	Omnivore	Enoplea	Dorylaimia	Dorylaimida	Ambiguous_taxa
R2_SV_558	Rhabditida	Tylenchidae	Filenchus	Filenchus discrepans strain FileDis2*	2e-144	94	KJ869311	Fungus feeder	Chromadorea	NA	Tylenchida	Filenchus discrepans
R2_SV_559	Desmodorida	Microlaimidae	Prodesmodora	Prodesmodora circulata*	1e-166	97.43	AY284721	Bacteria feeder	Chromadorea	NA	Chromadorida	Achromadora sp. JH-2004
R2_SV_563	Rhabditida	Pratylenchidae	Pratylenchus	Pratylenchus penetrans	3e-173	98.56	MH983023 etc	Plant feeder	Chromadorea	NA	Tylenchida	Pratylenchus penetrans
R2_SV_576	Monhysterida	Monhysteridae	Eumonhystera	Eumonhystera filiformis strain EumoFil2	0	100	AY593937	Bacteria feeder	Chromadorea	NA	Monhysterida	Eumonhystera filiformis
R2_SV_577	Monhysterida	Monhysteridae	Geomonhystera	Geomonhystera villosa*	2e-174	98.58	EF591334	Bacteria feeder	Chromadorea	NA	Monhysterida	Geomonhystera villosa
R2_SV_584	Rhabditida	Tylenchidae	Filenchus	Filenchus discrepans strain FileDis2	1e-142	93.71	KJ869311 etc	Fungus feeder	Chromadorea	NA	Tylenchida	Filenchus discrepans
R2_SV_594	Rhabditida	Ungellidae	Drasico	Drasico nemoralis	2e-79	83	KF573586	Parasite	Chromadorea	NA	Rhabditida	NA

Note: See notes for S3 Table.
