# Supplementary material for: Distinct community structures of soil nematodes from three ecologically different sites revealed by high-throughput amplicon sequencing of four 18S ribosomal RNA gene regions
Source: PLoS One. 2021 Apr 15;16(4):e0249571. doi: 10.1371/journal.pone.0249571 (PMC8049254; doi:10.1371/journal.pone.0249571)
Supplement: S5 Table — (PDF) [file pone.0249571.s005.pdf]

S5 Table. Nematode-derived SVs from region 3 and their taxa and feeding types based on a BLASTN search and SILVA database.

R3_SV	BLASTN data							Predicted feeding types	SILVA taxonomic data			
	Order	Family	Genus	Hit species used for taxonomy	E-value	% identity	Accession no.		D7	D8	D9	D10
R3_SV_1	Mononchida	Mylonchulidae, Mononchidae	Mylonchulus, Prionchulus	Mylonchulus sp., Prionchulus oleksandri	2e-149	100	AB361442 etc	Predator	Enoplea	Dorylaimia	Mononchida	Ambiguous_taxa
R3_SV_2	Dorylaimida	Longidoridae	Longidorus, Paralongidorus	Longidorus sp., Paralongidorus sp.	2e-149	100	MN129758 etc	Plant feeder	Enoplea	Dorylaimia	Dorylaimida	Ambiguous_taxa
R3_SV_4	Rhabditida	Rhabditidae	Distolabrellus	Distolabrellus veechi	6e-149	100	AF082999 etc	Bacteria feeder	Chromadorea	NA	Rhabditida	NA
R3_SV_5	Dorylaimida	Belondiridae	Dorylaimellus	Dorylaimellus parvulus isolate Barger 2	2e-149	100	AY911968	Plant feeder	Enoplea	Dorylaimia	Dorylaimida	NA
R3_SV_7	Plectida	Plectidae	Plectus	Plectus sp.	6e-149	100	LC382088 etc	Bacteria feeder	Chromadorea	NA	Araeolaimida	Ambiguous_taxa
R3_SV_10	Rhabditida	Thelastomatidae	Cephalobellus, Thelastoma, Travassosinema	Cephalobellus brevicaudatus, Thelastoma sp., Travassosinema sp. Ishigaki	6e-149	100	MF668724 etc	Parasite	Chromadorea	NA	Oxyurida	NA
R3_SV_11	Enoplida	Alaimidae	Alaimus	Alaimus sp. SSU1_19	5e-140	97.95	MG993561	Bacteria feeder	Enoplea	NA	NA	NA
R3_SV_12	Rhabditida	Cephalobidae	Acrobeloides, Cephalobus, Pseudacrobeles	Acrobeloides sp., Cephalobus sp. ILVO-C, Pseudacrobeles sp. OH-2016	6e-149	100	MK636581 etc	Bacteria feeder	Chromadorea	NA	Rhabditida	NA
R3_SV_13	Chromadorida	Cyatholaimidae	Achromadora	Achromadora cf terricola JH-2004, Achromadora sp. JH-2004	6e-149	100	AY593940 etc	Eucaryote feeder	Chromadorea	NA	Chromadorida	NA
R3_SV_14	Rhabditida	Criconematidae	Mesocriconema	Mesocriconema sp.	6e-149	100	MF095022 etc	Plant feeder	Chromadorea	NA	Tylenchida	NA
R3_SV_15	Rhabditida	Aphelenchidae	Aphelenchus	Aphelenchus sp.	6e-149	100	MT396111 etc	Fungus feeder	Chromadorea	NA	Tylenchida	Aphelenchus avenae
R3_SV_17	Rhabditida	Ungellidae	Drasico	Drasico nemoralis	6e-149	100	KF573586	Parasite?	Chromadorea	NA	Rhabditida	NA
R3_SV_19	Rhabditida	Criconematidae	Criconemoides, Discocriconemella	Criconemoides sp., Discocriconemella sinensis	6e-149	100	MN738714 etc	Plant feeder	Chromadorea	NA	Tylenchida	Hemicycliophora conida
R3_SV_22	Rhabditida	Pratylenchidae	Pratylenchus	Pratylenchus loosi	3e-147	99.66	LR215657	Plant feeder	Chromadorea	NA	Tylenchida	NA
R3_SV_25	Dorylaimida	Aporcelaimidae, Tylencholaimidae, Longidoridae, Qudsianematidae, Leptonchidae	Aporcella, Tylencholaimus, Xiphidorus, Lordellonema, Akrotonus, Funaria	Aporcella vitrinus, Tylencholaimus cf. teres, Xiphidorus sp., Lordellonema cf. parvum, Akrotonus vigor, Funaria cacti	2e-149	100	MG921235 etc	Fungus feeder/Omnivore/ Plant feeder	Enoplea	Dorylaimia	Dorylaimida	Ambiguous_taxa
R3_SV_26	Enoplida	Trischistomatidae	Trischistoma	Trischistoma sp.	5e-150	100	KR492034 etc	Predator	Enoplea	Enoplia	Triplonchida	NA
R3_SV_27	Triplonchida	Prismatolaimidae	Prismatolaimus	Prismatolaimus sp.	2e-149	100	LC186851 etc	Bacteria feeder	Enoplea	Enoplia	Triplonchida	Schizomidae environmental sample
R3_SV_28	Rhabditida	Tylenchidae, Merliniidae	Atetylenchus, Geocenamus, Psilenchus	Atetylenchus sp., Geocenamus chengi, Psilenchus sp.	7e-144	98.97	MN807627 etc	Plant feeder	Chromadorea	NA	Tylenchida	NA
R3_SV_29	Dorylaimida	Tylencholaimellidae, Actinolaimidae, Mydonomidae, Aporcelaimidae	Tylencholaimellus, Paraxonchium, Dorylaimoides, Doryllium	Tylencholaimellus sp., Paraxonchium magnidens, Dorylaimoides cf. elegans, Doryllium sp.	2e-149	100	KP835681 etc	Fungus feeder/Omnivore	Enoplea	Dorylaimia	Dorylaimida	Ambiguous_taxa
R3_SV_30	Rhabditida	Pratylenchidae	Pratylenchus	Pratylenchus sp.	1e-151	100	MH983023 etc	Plant feeder	Chromadorea	NA	Tylenchida	Pratylenchus penetrans
R3_SV_32	Dorylaimida	Aporcelaimidae	Aporcella, Aporcelaimus, Sectonema	Metaporcelaimus simplex, Sectonema sp., Aporcelaimus sp.,	2e-149	100	EF024622 etc	Omnivore	Enoplea	Dorylaimia	Dorylaimida	Ambiguous_taxa
R3_SV_34	Rhabditida	Rhabditidae	Distolabrellus	Distolabrellus veechi	3e-147	99.66	AF082999 etc	Bacteria feeder	Chromadorea	NA	Rhabditida	NA
R3_SV_36	Dorylaimida	Qudsianematidae, Dorylaimidae, Aporcelaimidae, Mydonomidae, Pararhyssocolpidae, Actinolaimidae, Belondiridae	Ecumenicus, Mesodorylaimus, Aporcelaimellus, Dorylaimoides, Pararhyssocolpus, Amblydorylaimus, Paractinolaimus, Axonchoides	Ecumenicus sp., Mesodorylaimus sp., Aporcelaimellus sp., Dorylaimoides sp., Rhyssocolpus paradoxus, Amblydorylaimus isokaryon, Paractinolaimus macrolaimus, Axonchoides sp.	2e-149	100	MK292127 etc	Omnivore?	Enoplea	Dorylaimia	Dorylaimida	Ambiguous_taxa
R3_SV_39	Triplonchida	Trichodoridae	Paratrichodorus	Paratrichodorus sp.*	2e-119	95.02	MG938581 etc	Plant feeder	Enoplea	Enoplia	Triplonchida	NA
R3_SV_40	Rhabditida	Rhabditidae	Diploscapter	Diploscapter sp.	6e-149	100	JQ838254 etc	Bacteria feeder	Chromadorea	NA	Rhabditida	Protorhabditis sp. JB122
R3_SV_42	Rhabditida	Cephalobidae	Acrobeloides, Cephalobus, Pseudacrobeles	Acrobeloides sp., Cephalobus sp., Pseudacrobeles sp.	1e-145	99.31	MK636581 etc	Bacteria feeder	Chromadorea	NA	Rhabditida	NA
R3_SV_43	Mononchida	Mylonchulidae	Mylonchulus, Prionchulus	Mylonchulus sp., Prionchulus oleksandri	8e-148	99.66	MG969498 etc	Predator	Enoplea	Dorylaimia	Mononchida	NA
R3_SV_44	Rhabditida	Aphelenchoididae	Aphelenchoides	Aphelenchoides sp.	1e-145	99.65	KY689014	Plant feeder	Chromadorea	NA	Tylenchida	NA
R3_SV_45	Rhabditida	Pratylenchidae	Pratylenchus	Pratylenchus sp.	3e-152	100	EU669926 etc	Plant feeder	Chromadorea	NA	Tylenchida	Pratylenchus penetrans
R3_SV_47	Chromadorida	Cyatholaimidae	Achromadora	Achromadora ruricola strain AchrRur	6e-149	100	AY593941	Eucaryote feeder	Chromadorea	NA	Chromadorida	NA
R3_SV_48	Rhabditida	Aphelenchoididae	Aphelenchoides	Aphelenchoides sp.	7e-144	98.47	KY769062 etc	Plant feeder	Chromadorea	NA	Tylenchida	Aphelenchoides bicaudatus
R3_SV_51	Dorylaimida	Belondiridae	Dorylaimellus	Dorylaimellus parvulus isolate Barger 2	9e-148	99.66	AY911968	Plant feeder	Enoplea	Dorylaimia	Dorylaimida	NA
R3_SV_53	Dorylaimida	Qudsianematidae, Nordiidae, Dorylaimidae	Allodorylaimus, Heterodorus, Rhyssocolpus, Enchodelus, Microdorylaimus, Prodorylaimus, Longidorella	Allodorylaimus sp., Heterodorus brevidentatus, Rhyssocolpus iuventutis, Enchodelus sp., Microdorylaimus sp., Prodorylaimus mas, Longidorella sp.	2e-149	100	KY942068 etc	Omnivore?	Enoplea	Dorylaimia	Dorylaimida	Ambiguous_taxa
R3_SV_54	Rhabditida	Aphelenchidae	Aphelenchus	Aphelenchus sp.	3e-147	99.66	MT396111 etc	Fungus feeder	Chromadorea	NA	Tylenchida	NA
R3_SV_58	Rhabditida	Tylenchidae	Discopersicus	Discopersicus iranicus	6e-149	100	KM502981	Plant feeder?	Chromadorea	NA	Tylenchida	NA
R3_SV_65	Dorylaimida	Qudsianematidae	Microdorylaimus, Thonus	Microdorylaimus angleus, Takamangai circulifera	2e-149	100	AY146526 etc	Omnivore	Enoplea	Dorylaimia	Dorylaimida	Ambiguous_taxa
R3_SV_68	Rhabditida	Criconematidae	Mesocriconema	Mesocriconema sp.	3e-147	99.66	MH983017 etc	Plant feeder	Chromadorea	NA	Tylenchida	NA
R3_SV_69	Rhabditida	Tylenchidae	Boleodorus, Basiria	Boleodorus sp., Basiria similis	2e-149	100	KT709462 etc	Plant feeder	Chromadorea	NA	Tylenchida	Boleodorus thylactus
R3_SV_71	Rhabditida	Aphelenchoididae	Aphelenchoides	Aphelenchoides sp.	1e-140	98.28	MN931593 etc	Plant feeder	Chromadorea	NA	Tylenchida	NA
R3_SV_72	Enoplida	Alaimidae	Alaimus	Alaimus sp. PDL-2005	1e-141	98.29	AJ966514	Bacteria feeder	Enoplea	Enoplia	Enoplida	Alaimus sp. PDL-2005
R3_SV_80	Rhabditida	Aphelenchoididae	Aphelenchoides	Aphelenchoides sp.	1e-140	98.26	MN931593 etc	Plant feeder	Chromadorea	NA	Tylenchida	NA
R3_SV_83	Dorylaimida	Qudsianematidae	Discolaimus	Discolaimus texanus isolate Konza IIICD-147	5e-150	100	AY146485	Predator	Enoplea	Dorylaimia	Dorylaimida	NA
R3_SV_84	Rhabditida	Tylenchidae	Filenchus	Filenchus cf. helenae TSH-2005 isolate Konza VIIEE-92	6e-149	100	AY912032	Fungus feeder	Chromadorea	NA	Tylenchida	Filenchus discrepans
R3_SV_86	Rhabditida	Cephalobidae	Acrobeloides, Cephalobus, Pseudacrobeles	Acrobeloides sp., Cephalobus sp., Pseudacrobeles sp.	3e-147	99.66	MK636581 etc	Bacteria feeder	Chromadorea	NA	Rhabditida	NA
R3_SV_87	Rhabditida	Rhabditidae	Distolabrellus	Distolabrellus veechi	3e-147	99.66	AF082999 etc	Bacteria feeder	Chromadorea	NA	Rhabditida	NA
R3_SV_88	Enoplida	Alaimidae	Alaimus	Alaimus sp. SSU1_19	5e-135	96.93	MG993561	Bacteria feeder	Enoplea	NA	NA	NA
R3_SV_89	Rhabditida	Cephalobidae	Cephalobus	Cephalobus cubaensis	6e-149	100	AF202161	Bacteria feeder	Chromadorea	NA	Rhabditida	Cephalobus cubaensis
R3_SV_90	Rhabditida	Hoplolaimidae, Heteroderidae	Helicotylenchus, Rotylenchus, Punctodera, Cactodera, Globodera	Helicotylenchus sp., Rotylenchus sp., Punctodera sp., Cactodera chenopodiae, Globodera sp.	2e-149	100	MT175368 etc	Plant feeder	Chromadorea	NA	Tylenchida	Rotylenchulus reniformis
R3_SV_92	Rhabditida	Rhabditidae	Distolabrellus	Distolabrellus veechi	3e-147	99.66	AF082999 etc	Bacteria feeder	Chromadorea	NA	Rhabditida	NA
R3_SV_95	Triplonchida	Tripylidae	Tripylella	Tripylella sp. ZQZ-2010a isolate R10	2e-148	99.66	GQ503074	Predator	Enoplea	Enoplia	Triplonchida	NA
R3_SV_98	Rhabditida	Tylenchidae, Anguinidae	Discotylenchus, Filenchus, Ditylenchus	Discotylenchus sp., Filenchus misellus, Ditylenchus brevicauda	5e-150	100	MK301105 etc	Fungus feeder/Plant feeder	Chromadorea	NA	Tylenchida	NA
R3_SV_100	Dorylaimida	Belondiridae, Aporcelaimidae	Axonchium, Aporcelaimus, Dactyluraxonchium	Axonchium sp., Aporcelaimus sp., Dactyluraxonchium sp.	2e-149	100	MG921264 etc	Plant feeder/Omnivore	Enoplea	Dorylaimia	Dorylaimida	Ambiguous_taxa
R3_SV_103	Enoplida	Trischistomatidae	Trischistoma	Trischistoma sp.	1e-136	97.21	KR492034 etc	Predator	Enoplea	Enoplia	Triplonchida	NA
R3_SV_104	Rhabditida	Pratylenchidae	Pratylenchus	Pratylenchus sp.	1e-150	99.66	EU669926 etc	Plant feeder	Chromadorea	NA	Tylenchida	Pratylenchus penetrans
R3_SV_107	Rhabditida	Tylenchidae	Basiria	Basiria gracilis isolate fafu01	5e-150	100	MK639394	Plant feeder	Chromadorea	NA	Tylenchida	Basiria gracilis
R3_SV_113	Rhabditida	Pratylenchidae	Pratylenchus	Pratylenchus penetrans	1e-150	99.66	EU669926 etc	Plant feeder	Chromadorea	NA	Tylenchida	Pratylenchus penetrans

R3_SV_114	Dorylaimida	Nygolaimidae	Clavicaudoides, Aquatides	Clavicaudoides sp., Aquatides christei	8e-148	99.66	AY552967 etc	Predator	Enoplea	Dorylaimia	Dorylaimida	NA
R3_SV_122	Mononchida	Mylonchulidae	Mylonchulus	Mylonchulus mulveyi	2e-149	100	AB361449 etc	Predator	Enoplea	Dorylaimia	Mononchida	NA
R3_SV_123	Rhabditida	Aphelenchoididae	Aphelenchoides	Aphelenchoides sp.	3e-142	98.63	MN931593 etc	Plant feeder	Chromadorea	NA	Tylenchida	NA
R3_SV_126	Rhabditida	Pratylenchidae	Pratylenchus	Pratylenchus sp.	2e-148	99.33	MH983023 etc	Plant feeder	Chromadorea	NA	Tylenchida	Pratylenchus penetrans
R3_SV_128	Rhabditida	Meloidogynidae	Meloidogyne	Meloidogyne sp.	1e-150	100	MF177719 etc	Plant feeder	Chromadorea	NA	Tylenchida	NA
R3_SV_129	Triplonchida	Odontolaimidae	Odontolaimus	Odontolaimus sp. OdLaSp1*	1e-125	95.49	FJ969131	Bacteria feeder	Enoplea	Enoplia	Triplonchida	NA
R3_SV_132	Rhabditida	Ecphyadophoridae	Lelenchus	Lelenchus sp.	5e-135	97.24	MN542205 etc	Plant feeder	Chromadorea	NA	Tylenchida	NA
R3_SV_139	Dorylaimida	Nygolaimidae	Clavicaudoides, Aquatides	Clavicaudoides sp., Aquatides christei	2e-144	98.97	AY552967 etc	Predator	Enoplea	Dorylaimia	Dorylaimida	Nygolaimus cf. brachyuris JH-2004
R3_SV_140	Rhabditida	Rhabditidae	Pellioditis	Pellioditis marina strain SB178	2e-143	98.97	AF083021	Bacteria feeder	Chromadorea	NA	Rhabditida	Ambiguous_taxa
R3_SV_141	Dorylaimida	Belondiridae, Carcharolaimidae	Dorylaimellus, Carcharolaimus,	Dorylaimellus virginianus, Carcharolaimus teres	2e-149	100	AY552969 etc	Plant feeder/Predator	Enoplea	Dorylaimia	Dorylaimida	NA
R3_SV_144	Rhabditida	Meloidogynidae	Meloidogyne	Meloidogyne sp.	7e-149	99.66	MF177719 etc	Plant feeder	Chromadorea	NA	Tylenchida	NA
R3_SV_148	Enoplida	Trischistomatidae	Trischistoma	Trischistoma sp.	5e-140	97.95	KR492034 etc	Predator	Enoplea	Enoplia	Triplonchida	NA
R3_SV_149	Dorylaimida	Nygolaimidae	Clavicaudoides, Aquatides, Nygolaimus	Clavicaudoides sp., Aquatides christei, Nygolaimus cf. brachyuris JH-2004	4e-146	99.32	AY552967 etc	Predator	Enoplea	Dorylaimia	Dorylaimida	NA
R3_SV_151	Rhabditida	Rhabditidae	Distolabrellus	Distolabrellus veechi	1e-145	99.31	AF082999 etc	Bacteria feeder	Chromadorea	NA	Rhabditida	NA
R3_SV_153	Enoplida	Trischistomatidae	Trischistoma	Trischistoma sp.	7e-119	93.81	KR492034 etc	Predator	Enoplea	Enoplia	Triplonchida	NA
R3_SV_158	Enoplida	Trischistomatidae	Trischistoma	Trischistoma sp.	2e-143	98.63	KR492034 etc	Predator	Enoplea	Enoplia	Triplonchida	NA
R3_SV_167	Rhabditida	Aphelenchidae	Aphelenchus	Aphelenchus sp.	1e-145	99.31	MT396111 etc	Fungus feeder	Chromadorea	NA	Tylenchida	NA
R3_SV_192	Monhysterida	Monhysteridae	Geomonhystera	Geomonhystera sp.	7e-144	98.97	EF591334 etc	Bacteria feeder	Chromadorea	NA	Monhysterida	Geomonhystera villosa

Note: See notes on S3 Table.
