# Supplementary material for: Distinct community structures of soil nematodes from three ecologically different sites revealed by high-throughput amplicon sequencing of four 18S ribosomal RNA gene regions
Source: PLoS One. 2021 Apr 15;16(4):e0249571. doi: 10.1371/journal.pone.0249571 (PMC8049254; doi:10.1371/journal.pone.0249571)
Supplement: S6 Table — (PDF) [file pone.0249571.s006.pdf]

S6 Table. Nematode-derived SVs from region 4 and their taxa and feeding types based on a BLASTN search and SILVA database.

R4_SV	BLASTN data							Predicted feeding types	SILVA taxonomic data			
	Order	Family	Genus	Hit species used for taxonomy	E-value	% identity	Accession no.		D7	D8	D9	D10
R4_SV_1	Dorylaimida	Dorylaimidae	Laimydorus	Laimydorus sp. TX1-07*	7e-159	98.75	AY146502	Omnivore	Enoplea	Dorylaimia	Dorylaimida	NA
R4_SV_2	Mononchida	Mononchidae, Mylonchulidae	Prionchulus, Mylonchulus	Prionchulus oleksandri, Mylonchulus cf. incurvus	4e-166	100	MG969498 etc	Predator	Enoplea	Dorylaimia	Mononchida	Mylonchulus hawaiiensis
R4_SV_7	Dorylaimida	Belondiridae	Dorylaimellus	Dorylaimellus parvulus	2e-165	100	AY911968	Plant feeder	Enoplea	Dorylaimia	Dorylaimida	Proleptonchus weischeri
R4_SV_12	Rhabditida	Rhabditidae	Distolabrellus	Distolabrellus veechi	3e-163	100	AF082999	Bacteria feeder	Chromadorea	NA	Rhabditida	NA
R4_SV_14	Rhabditida	Thelastomatidae, Travassosinematidae, Cosmocercidae	Aorurus, Thelastoma, Travassosinema, Raillietnema	Aorurus agile*, Thelastoma sp.*, Travassosinema sp.*, Raillietnema sp. *	6e-165	99.06	MN190720 etc	Parasite	Chromadorea	NA	Rhabditida	Ambiguous_taxa
R4_SV_15	Plectida	Plectidae	Plectus	Plectus sp.	2e-165	100	LC382088 etc	Bacteria feeder	Chromadorea	NA	Araeolaimida	NA
R4_SV_17	Enoplida	Alaimidae	Alaimus	Alaimus sp.*	3e-157	98.44	LC186877 etc	Bacteria feeder	Enoplea	Enoplia	Enoplida	NA
R4_SV_18	Dorylaimida	Dorylaimidae	Laimydorus	Laimydorus sp. TX1-07*	2e-160	99.07	AY146502	Omnivore	Enoplea	Dorylaimia	Dorylaimida	NA
R4_SV_21	Rhabditida	Rhabditidae	Distolabrellus	Distolabrellus veechi	9e-163	100	AF082999	Bacteria feeder	Chromadorea	NA	Rhabditida	NA
R4_SV_24	Rhabditida	Cephalobidae	Acrobeloides	Acrobeloides varius	2e-165	100	MK636581 etc	Bacteria feeder	Chromadorea	NA	Rhabditida	NA
R4_SV_25	Rhabditida	Criconematidae	Mesocriconema	Mesocriconema sp.	6e-165	100	MH983017 etc	Plant feeder	Chromadorea	NA	Rhabditida	Mesocriconema xenoplax
R4_SV_26	Rhabditida	Aphelenchidae	Aphelenchus	Aphelenchus sp.	9e-163	99.69	KX356730 etc	Fungus feeder	Chromadorea	NA	Rhabditida	Aphelenchus avenae
R4_SV_27	Chromadorida	Cyatholaimidae	Achromadora	Achromadora cf terricola JH-2004	2e-164	99.69	AY593940	Eucaryote feeder	Chromadorea	NA	Chromadorida	Achromadora cf terricola JH-2004
R4_SV_30	Rhabditida	Ungellidae	Drasico	Drasico nemoralis	1e-151	97.52	KF573586	Parasite?	Chromadorea	NA	Rhabditida	Drasico nemoralis
R4_SV_31	Plectida, Rhabditida, Dorylaimida	Plectidae, Cephalobidae, Dorylaimidae	Plectus, Acrobeloides, Ceratoplectus, Wilsonema	Plectus sp., Acrobeloides buetschlii, Ceratoplectus cf. armatus ITDL-2009, Wilsonema sp.	2e-165	100	LC186814 etc	Bacteria feeder	Chromadorea	NA	Araeolaimida	Acrobeloides buetschlii
R4_SV_33	Rhabditida	Criconematidae	Criconemoides	Criconemoides parainformis	2e-165	100	MN738711	Plant feeder	Chromadorea	NA	Rhabditida	Hemicycliophora conida
R4_SV_34	Mononchida	Mylonchulidae	Mylonchulus	Mylonchulus sp.	4e-166	100	LC186773 etc	Predator	Enoplea	Dorylaimia	Mononchida	Ambiguous_taxa
R4_SV_36	Rhabditida	Cephalobidae	Eucephalobus, Cephalobus	Eucephalobus sp., Cephalobus sp. Konza	2e-165	100	AY912001 etc	Bacteria feeder	Chromadorea	NA	Rhabditida	NA
R4_SV_37	Triplonchida	Diphtherophoridae	Diphtherophora	Diphtherophora sp. Shahrood*	5e-121	92.38	KY115102	Fungus feeder	NA	NA	NA	NA
R4_SV_38	Rhabditida	Pratylenchidae	Pratylenchus	Pratylenchus sp.	6e-165	100	MN756509 etc	Plant feeder	Chromadorea	NA	Rhabditida	Pratylenchus penetrans
R4_SV_39	Rhabditida	Pratylenchidae	Pratylenchus	Pratylenchus loosi	2e-165	100	LR215657	Plant feeder	Chromadorea	NA	Rhabditida	unidentified
R4_SV_41	Dorylaimida	Tylencholaimidae	Tylencholaimus, Capilonchus	Tylencholaimus sp. PDL-2005*, Cf. Capilonchus sp.*	4e-151	97.2	AJ966510 etc	Fungus feeder	Enoplea	Dorylaimia	Dorylaimida	NA
R4_SV_42	Triplonchida	Prismatolaimidae	Prismatolaimus	Prismatolaimus sp.	2e-165	100	LC186851 etc	Bacteria feeder	Enoplea	Enoplia	Triplonchida	Ambiguous_taxa
R4_SV_43	Enoplida	Trischistomatidae	Trischistoma	Trischistoma sp.	6e-165	100	JN673802 etc	Predator	Enoplea	Enoplia	Triplonchida	Trischistoma triregium
R4_SV_45	Rhabditida	Tylenchidae	Discopersicus, Basiria	Discopersicus iranicus, Basiria sp.	2e-154	98.12	KM502981 etc	Plant feeder	Chromadorea	NA	Rhabditida	Basiria duplexa
R4_SV_47	Dorylaimida	Tylencholaimellidae	Tylencholaimellus	Tylencholaimellus striatus isolate Konza IAD-128*	7e-164	99.69	AY146530	Fungus feeder	Enoplea	Dorylaimia	Dorylaimida	NA
R4_SV_57	Rhabditida	Ungellidae	Drasico	Drasico nemoralis	6e-150	97.2	KF573586	Parasite	Chromadorea	NA	Rhabditida	Drasico nemoralis
R4_SV_58	Dorylaimida	Aporcelaimidae	Sectonema, Aporcelaimus, Aporcelaimellus	Sectonema sp.*, Aporcelaimus sp.*, Aporcelaimellus sp.*	2e-165	99.69	AY284815 etc	Omnivore	Enoplea	Dorylaimia	Dorylaimida	Poduridae environmental sample
R4_SV_59	Rhabditida	Aphelenchoidinae	Aphelenchoides	Aphelenchoides sp. Asp25*	7e-139	95.28	KY769062	Plant feeder	Chromadorea	NA	Rhabditida	metagenome
R4_SV_62	Rhabditida	Aphelenchoidinae	Aphelenchoides	Aphelenchoidinae sp.	9e-163	100	AB631006	Plant feeder	Chromadorea	NA	Rhabditida	metagenome
R4_SV_63	Dorylaimida	Dorylaimidae	Mesodorylaimus	Mesodorylaimus cf. recurvus Konza IAD-37	4e-166	100	AY146489	Omnivore	Enoplea	Dorylaimia	Dorylaimida	NA
R4_SV_70	Rhabditida	Cephalobidae	Eucephalobus	Eucephalobus striatus isolate EuceStr2*	2e-155	98.13	AY284667	Bacteria feeder	Chromadorea	NA	Rhabditida	NA
R4_SV_73	Rhabditida	Rhabditidae	Diploscapter	Diploscapter coronatus strain DiScCor2	3e-168	100	KJ636377	Bacteria feeder	Chromadorea	NA	Rhabditida	Protorhabditis sp. JB122
R4_SV_76	Chromadorida	Cyatholaimidae	Achromadora	Achromadora cf terricola JH-2004*	3e-148	96.6	AY593940	Eucaryote feeder	Chromadorea	NA	Chromadorida	Achromadora cf terricola JH-2004
R4_SV_86	Chromadorida	Cyatholaimidae	Achromadora	Achromadora sp.	4e-166	100	LC186707 etc	Eucaryote feeder	Chromadorea	NA	Chromadorida	NA
R4_SV_87	Rhabditida	Meloidogynidae	Meloidogyne	Meloidogyne sp.	6e-165	100	MF177719 etc	Plant feeder	Chromadorea	NA	Rhabditida	NA
R4_SV_93	Chromadorida	Cyatholaimidae	Achromadora	Achromadora sp. JH-2004	4e-166	100	AY284717	Eucaryote feeder	Chromadorea	NA	Chromadorida	Achromadora sp. JH-2004
R4_SV_97	Rhabditida	Aphelenchidae	Aphelenchus	Aphelenchus avenae	2e-164	100	EU306347	Fungus feeder	Chromadorea	NA	Rhabditida	Aphelenchus avenae
R4_SV_103	Triplonchida	Prismatolaimidae	Prismatolaimus	Prismatolaimus sp.	2e-165	100	LC186858 etc	Bacteria feeder	Enoplea	Enoplia	Triplonchida	Ambiguous_taxa
R4_SV_108	Rhabditida	Aphelenchoididae	Aphelenchoides	Aphelenchoides sp.	7e-134	94.39	MK301114	Plant feeder	Chromadorea	NA	Rhabditida	NA
R4_SV_111	Dorylaimida	Qudsianematidae	Eudorylaimus	cf. Eudorylaimus carteri MtR GWN-02	4e-166	100	AY146523	Omnivore	Enoplea	Dorylaimia	Dorylaimida	NA
R4_SV_112	Rhabditida	Tylenchidae	Discopersicus, Basiria	Discopersicus iranicus, Basiria duplexa	6e-165	100	KM502981 etc	Plant feeder	Chromadorea	NA	Rhabditida	Basiria duplexa
R4_SV_117	Plectida	Plectidae	Plectus	Plectus sp.	2e-165	100	LC186667 etc	Bacteria feeder	Chromadorea	NA	Araeolaimida	NA
R4_SV_118	Chromadorida	Cyatholaimidae	Achromadora	Achromadora cf terricola JH-2004*	1e-151	97.22	AY593940	Eucaryote feeder	Chromadorea	NA	Chromadorida	Achromadora cf terricola JH-2004
R4_SV_121	Triplonchida	Diphtherophoridae	Diphtherophora	Diphtherophora sp. Shahrood*	2e-129	93.21	KY115102	Fungus feeder	Enoplea	Enoplia	Triplonchida	NA
R4_SV_129	Dorylaimida	Qudsianematidae	Microdorylaimus	Microdorylaimus sp.	2e-164	99.69	AY284804 etc	Omnivore	Enoplea	Dorylaimia	Dorylaimida	Enchodelus veletensis
R4_SV_133	Rhabditida	Aphelenchoididae	Aphelenchoides	Aphelenchoides sp.	2e-135	94.67	KY964615 etc	Plant feeder	Chromadorea	NA	Rhabditida	NA
R4_SV_149	Rhabditida	Tylenchidae	Boleodorus	Boleodorus volutus strain BoleVol1*	6e-155	97.56	FJ969117	Plant feeder	Chromadorea	NA	Rhabditida	Boleodorus thylactus
R4_SV_150	Rhabditida	Aphelenchidae	Aphelenchus	Aphelenchus sp.	4e-161	99.37	KX356730 etc	Fungus feeder	Chromadorea	NA	Rhabditida	Aphelenchus avenae
R4_SV_158	Rhabditida	Aphelenchidae	Aphelenchus	Aphelenchus sp.	2e-159	99.06	KX356730 etc	Fungus feeder	Chromadorea	NA	Rhabditida	Aphelenchus avenae
R4_SV_159	Enoplida	Alaimidae	Alaimus	Alaimus sp.	7e-164	99.69	LC186877 etc	Bacteria feeder	Enoplea	Enoplia	Enoplida	Alaimus sp. PDL-2005
R4_SV_161	Dorylaimida	Qudsianematidae	Allodorylaimus, Eudorylaimus	Allodorylaimus sp., Eudorylaimus cf. subdigitalis	4e-166	100	KY942068 etc	Omnivore	Enoplea	Dorylaimia	Dorylaimida	NA
R4_SV_168	Rhabditida	Ungellidae	Drasico	Drasico nemoralis	1e-116	91.19	KF573586	Parasite	Chromadorea	NA	Rhabditida	Drasico nemoralis
R4_SV_170	Rhabditida	Meloidogynidae	Meloidogyne	Meloidogyne sp.	6e-165	100	LN713297 etc	Plant feeder	Chromadorea	NA	Rhabditida	Meloidogyne incognita (southern root-knot nematode)
R4_SV_175	Rhabditida	Meloidogynidae	Meloidogyne	Meloidogyne sp.	3e-163	99.69	KU666392 etc	Plant feeder	Chromadorea	NA	Rhabditida	Meloidogyne incognita (southern root-knot nematode)
R4_SV_182	Dorylaimida	Mydonomidae, Dorylaimidae	Dorylaimoides, Thornenema	Dorylaimoides sp.*, Thornenema cf. laevicapitatum*	7e-164	99.69	AY593950 etc	Fungus feeder/Omnivore	Enoplea	Dorylaimia	Dorylaimida	NA
R4_SV_184	Rhabditida	Cephalobidae	Heterocephalobus	Heterocephalobus elongatus*	2e-160	99.07	AY284670 etc	Bacteria feeder	Chromadorea	NA	Rhabditida	Heterocephalobus elongatus
R4_SV_186	Rhabditida	Hoplolaimidae	Helicotylenchus	Helicotylenchus sp.	9e-163	99.38	KJ869416 etc	Plant feeder	Chromadorea	NA	Rhabditida	NA
R4_SV_201	Rhabditida	Aphelenchidae	Aphelenchus	Aphelenchus sp.	4e-161	99.37	KX356730 etc	Fungus feeder	Chromadorea	NA	Rhabditida	Aphelenchus avenae
R4_SV_208	Rhabditida	Meloidogynidae	Meloidogyne	Meloidogyne sp.	3e-163	99.69	MT102326 etc	Plant feeder	Chromadorea	NA	Rhabditida	NA
R4_SV_215	Rhabditida	Tylenchidae	Filenchus	Filenchus discrepans	6e-165	99.69	KJ869311 etc	Fungus feeder	Chromadorea	NA	Rhabditida	Filenchus discrepans
R4_SV_219	Rhabditida	Cephalobidae	Cervidellus, Pseudacrobeles	Cervidellus sp. JH-2004, Pseudacrobeles variabilis	2e-160	99.07	AY284674 etc	Bacteria feeder	Chromadorea	NA	Rhabditida	NA
R4_SV_222	Rhabditida	Panagrolaimidae	Propanagrolaimus	Propanagrolaimus sp.	3e-73	83.18	KJ434176 etc	Bacteria feeder	Chromadorea	NA	Rhabditida	Propanagrolaimus filiformis
R4_SV_233	Rhabditida	Cephalobidae	Cephalobus	Cephalobus cubaensis	2e-165	99.38	AF202161	Bacteria feeder	Chromadorea	NA	Rhabditida	Cephalobus cubaensis
R4_SV_235	Rhabditida	Cosmocercidae, Thelastomatidae	Cosmocerca, Cephalobellus	Cosmocerca simile*, Cephalobellus brevicaudatus*	3e-158	98.75	MN839758 etc	Parasite	Chromadorea	NA	Rhabditida	Ambiguous_taxa
R4_SV_237	Triplonchida	Prismatolaimidae	Prismatolaimus	Prismatolaimus sp.	2e-165	100	LC186686 etc	Bacteria feeder	Enoplea	Enoplia	Triplonchida	Ambiguous_taxa
R4_SV_246	Rhabditida	Meloidogynidae	Meloidogyne	Meloidogyne ichinohei isolate MeloIch3*	5e-111	90.06	KC875385	Plant feeder	Chromadorea	NA	Rhabditida	NA
R4_SV_251	Enoplida	Trischistomatidae	Trischistoma	Trischistoma sp.	6e-155	98.12	JN673802 etc	Predator	Enoplea	Enoplia	Triplonchida	NA
R4_SV_255	Mononchida	Mylonchulidae	Mylonchulus	Mylonchulus mulveyi	6e-165	100	AB361449 etc	Predator	Enoplea	Dorylaimia	Mononchida	Mylonchulus mulveyi
R4_SV_273	Triplonchida	Tripylidae	Tripylella	Tripylella sp. 1031	7e-149	96.88	FJ040488	Predator	Enoplea	Enoplia	Triplonchida	NA
R4_SV_287	Rhabditida	Ecphyadophoridae	Lelenchus	Lelenchus sp. MB-2019	9e-148	97.15	MN542205	Plant feeder	Chromadorea	NA	Rhabditida	NA
R4_SV_293	Rhabditida	Tylenchidae	Filenchus	Filenchus sp.	9e-168	100	LC186881 etc	Fungus feeder	Chromadorea	NA	Rhabditida	Filenchus misellus
R4_SV_296	Monhysterida	Monhysteridae	Eumonhystera	Eumonhystera filiformis*	2e-155	98.13	AY593937 etc	Bacteria feeder	Chromadorea	NA	Monhysterida	NA
R4_SV_326	Dorylaimida	Nygolaimidae	Clavicaudoides, Aquatides	Clavicaudoides sp. , Aquatides christei	3e-167	100	AY552967 etc	Predator	Enoplea	Dorylaimia	Dorylaimida	Aquatides aquaticus
R4_SV_335	Triplonchida	Diphtherophoridae	Diphtherophora	Diphtherophora sp. Shahrood*	5e-116	91.43	KY115102	Fungus feeder	Enoplea	Enoplia	Triplonchida	NA
R4_SV_338	Dorylaimida	Tylencholaimidae	Tylencholaimus	Tylencholaimus sp.*	3e-158	98.45	LC186596 etc	Fungus feeder	Enoplea	Dorylaimia	Dorylaimida	NA

R4_SV_350	Plectida	Plectidae	Plectus	Plectus sp.	2e-145	96.26	LC186667 etc	Bacteria feeder	Chromadorea	NA	Araeolaimida	NA
R4_SV_360	Monhysterida	Monhysteridae	Eumonhystera	Eumonhystera filiformis*	3e-162	99.38	AY593937 etc	Bacteria feeder	Chromadorea	NA	Monhysterida	Paralamyctes environmental sample
R4_SV_372	Mononchida	Mononchidae	Prionchulus	Prionchulus oleksandri*	4e-161	99.07	MG969498	Predator	Enoplea	Dorylaimia	Mononchida	NA
R4_SV_385	Rhabditida	Aphelenchidae	Aphelenchus	Aphelenchus sp.	2e-129	92.71	KX356730 etc	Fungus feeder	Chromadorea	NA	Rhabditida	Aphelenchus avenae
R4_SV_387	Enoplida	Trischistomatidae	Trischistoma	Trischistoma sp.	7e-134	94.62	JN673802 etc	Predator	Enoplea	Enoplia	Triplonchida	Trischistoma triregium
R4_SV_433	Dorylaimida	Nordiidae, Dorylaimidae	Pungentus, Mesodorylaimus, Calcaridorylaimus	Pungentus cf. crassus*, Mesodorylaimus sp.*, Calcaridorylaimus sp.*	2e-90	97.06	AY146529 etc	Omnivore	Enoplea	Dorylaimia	Dorylaimida	NA
R4_SV_435	Dorylaimida	Nordiidae	Pungentus	Pungentus cf. crassus*	2e-90	97.07	AY146529	Omnivore	Enoplea	Dorylaimia	Dorylaimida	NA

Note: See notes on S3 Table.
