# Supplementary material for: Distinct community structures of soil nematodes from three ecologically different sites revealed by high-throughput amplicon sequencing of four 18S ribosomal RNA gene regions
Source: PLoS One. 2021 Apr 15;16(4):e0249571. doi: 10.1371/journal.pone.0249571 (PMC8049254; doi:10.1371/journal.pone.0249571)
Supplement: S7 Table — (PDF) [file pone.0249571.s007.pdf]

S7 Table. Novel nematode-derived SVs from three nematode communities in four SSU regions.

SV name[a]	BLASTN data[b]							Predicted feeding types[c]
	Order	Family	Genus	Hit species used for taxonomy	E-value	%id (hit)	%id (top)	
R1_SV_34	Chromadorida	Cyatholaimidae	Achromadora	Achromadora cf terricola JH-2004	1e-177	98.88		Eucaryote feeder
R1_SV_54	Chromadorida	Cyatholaimidae	Achromadora	Achromadora cf terricola JH-2004	1e-172	98.03		Eucaryote feeder
R1_SV_194	Chromadorida	Cyatholaimidae	Achromadora	Achromadora cf terricola JH-2004	5e-176	98.6		Eucaryote feeder
R1_SV_295	Chromadorida	Cyatholaimidae	Achromadora	Achromadora ruricola*	8e-174	98.31	98.87	Eucaryote feeder
R1_SV_476	Chromadorida	Cyatholaimidae	Achromadora	Achromadora ruricola	1e-157	95.75		Eucaryote feeder
R1_SV_543	Chromadorida	Cyatholaimidae	Achromadora	Achromadora cf terricola JH-2004	1e-167	97.98		Eucaryote feeder
R1_SV_36	Dorylaimida	Aporcelaimidae	Sectonema	Sectonema barbatoides	8e-174	97.78		Omnivore
R1_SV_100	Dorylaimida	Belondiridae	Dorylaimellus	Dorylaimellus virginianus	4e-172	97.5		Plant feeder
R1_SV_293	Dorylaimida	Nygolaimidae	Clavicaudoides	Clavicaudoides sp. PGM-2004	2e-175	97.81		Predator
R1_SV_37	Dorylaimida	Qudsianematidae, Actinolaimidae	Ecumenicus, Paractinolaimus	Ecumenicus monohystera, Paractinolaimus macrolaimus	2e-170	97.22		Omnivore
R1_SV_316	Dorylaimida	Qudsianematidae, Nordiidae	Epidorylaimus, Enchodelus	Epidorylaimus sp. 1457, Enchodelus sp. JH-2004	2e-135	91.41		Omnivore /Predator
R1_SV_424	Enoplida	Trischistomatidae	Trischistoma	Trischistoma monohystera	3e-123	90.23		Predator
R1_SV_169	Enoplida	Alaimidae	Alaimus	Alaimus sp. PDL-2005*	1e-177	98.6	98.88	Bacteria feeder
R1_SV_231	Enoplida	Alaimidae	Alaimus	Alaimus sp. PDL-2005*	2e-174	98.04	98.32	Bacteria feeder
R1_SV_562	Mononchida	Mylonchulidae	Mylonchulus	Mylonchulus sp.	2e-170	96.97		Predator
R1_SV_594	Mononchida	Mylonchulidae	Mylonchulus	Mylonchulus sp.	1e-166	96.42		Predator
R1_SV_371	Plectida	Plectidae	Plectus	Plectus sp.	2e-175	98.32		Bacteria feeder
R1_SV_556	Rhabditida	Aphelenchoididae	Aphelenchoides	Aphelenchoides sp.*	1e-131	91.95	98.66	Plant feeder
R1_SV_119	Rhabditida	Cephalobidae	Acrobeloides	Acrobeloides sp.*	1e-172	97.77	98.32	Bacteria feeder
R1_SV_184	Rhabditida	Cephalobidae	Acrobeloides	Acrobeloides sp.*	1e-172	97.77	98.32	Bacteria feeder
R1_SV_507	Rhabditida	Ecphyadophoridae	Lelenchus	Lelenchus sp.	1e-146	93.58		Plant feeder
R1_SV_171	Rhabditida	Rhabditidae	Distolabrellus	Distolabrellus veechi	1e-87	87.14		Bacteria feeder
R1_SV_180	Rhabditida	Rhabditidae	Distolabrellus	Distolabrellus veechi	3e-84	86.5		Bacteria feeder
R1_SV_22	Rhabditida	Thelastomatidae	Severianoia	Severianoia sp. 1 SVM-2019	4e-177	98.6		Parasite
R1_SV_123	Rhabditida	Thelastomatidae	Severianoia	Severianoia sp. 1 SVM-2019	4e-177	98.6		Parasite
R1_SV_200	Rhabditida	Thelastomatidae	Severianoia	Severianoia sp. 1 SVM-2019	2e-175	98.32		Parasite
R1_SV_310	Rhabditida	Thelastomatidae	Severianoia	Severianoia sp. 1 SVM-2019	2e-175	98.32		Parasite
R1_SV_104	Rhabditida	Tylenchidae	Basiria	Basiria sp.	0	98.9		Plant feeder
R1_SV_170	Rhabditida	Tylenchidae	Basiria	Basiria sp.	6e-180	98.62		Plant feeder
R1_SV_298	Rhabditida	Tylenchidae	Miculenchus	Miculenchus salvus*	2e-155	94.96	98.60	Plant feeder
R1_SV_578	Rhabditida	Tylenchidae	Filenchus	Ottolenchus longiurus	3e-163	96.36		Fungus feeder
R1_SV_24	Rhabditida	Ungellidae	Drasico	Drasico paludigenus	6e-180	98.89		Parasite
R1_SV_57	Rhabditida	Ungellidae	Drasico	Drasico paludigenus	3e-178	98.61		Parasite
R1_SV_521	Rhabditida	Ungellidae	Drasico	Drasico paludigenus	1e-161	95.82		Parasite
R1_SV_110	Triplonchida	Diphtherophoridae	Diphtherophora	Diphterophora communis*	3e-123	89.66	96.65	Fungus feeder
R1_SV_304	Triplonchida	Tripylidae	Tripylella	Tripylella sp. 1031	2e-154	95.17		Predator
R2_SV_110	Chromadorida	Cyatholaimidae	Achromadora	Achromadora cf terricola JH-2004	1e-167	97.71		Eucaryote feeder
R2_SV_147	Chromadorida	Cyatholaimidae	Achromadora	Achromadora sp. JH-2004*	1e-167	97.43	97.71	Eucaryote feeder
R2_SV_164	Chromadorida	Cyatholaimidae	Achromadora	Achromadora cf terricola JH-2004	2e-169	98		Eucaryote feeder
R2_SV_430	Chromadorida	Cyatholaimidae	Achromadora	Achromadora sp. JH-2004*	2e-164	96.87	97.15	Eucaryote feeder
R2_SV_471	Chromadorida	Cyatholaimidae	Achromadora	Achromadora sp. JH-2004 isolate AchrSp2	5e-166	97.14		Eucaryote feeder
R2_SV_262	Desmodorida	Microlaimidae	Prodesmodora	Prodesmodora circulata*	3e-158	96	96.86	Bacteria feeder
R2_SV_559	Desmodorida	Microlaimidae	Prodesmodora	Prodesmodora circulata*	1e-166	97.43	97.71	Bacteria feeder
R2_SV_362	Desmodorida, Chromadorida	Microlaimidae, Cyatholaimidae	Prodesmodora, Achromadora	Prodesmodora circulata*, Achromadora sp. JH-2004*	6e-160	96.29	97.14	Bacteria feeder/Eeukayote feeder
R2_SV_531	Desmodorida, Chromadorida	Microlaimidae, Cyatholaimidae	Prodesmodora, Achromadora	Prodesmodora circulata*, Achromadora sp. JH-2004*	6e-160	96.29	97.14	Bacteria feeder/Eeukayote feeder
R2_SV_41	Dorylaimida	Leptonchidae	Proleptonchus	Proleptonchus weischeri	3e-173	98.57		Fungus feeder
R2_SV_200	Dorylaimida	Mydonomidae	Dorylaimoides	Dorylaimoides sp.	6e-175	98.86		Fungus feeder
R2_SV_317	Dorylaimida	Nygolaimidae	Clavicaudoides, Aquatides	Clavicaudoides sp. PGM-2004, Aquatides christei isolate Konza IIAC-02	1e-177	98.88		Predator
R2_SV_338	Dorylaimida	Dorylaimidae, Actinolaimidae, Qudsianematidae	Mesodorylaimus, Paractinolaimus, Labronema	Mesodorylaimus sp., Paractinolaimus macrolaimus, Labronema vulvapapillatum	6e-165	97.14		Omnivore
R2_SV_490	Dorylaimida	Qudsianematidae, Dorylaimidae	Epidorylaimus, Prodorylaimus	Epidorylaimus sp., Prodorylaimus mas	3e-148	94.3		Omnivore
R2_SV_499	Dorylaimida	Qudsianematidae, Dorylaimidae	Epidorylaimus, Prodorylaimus	Epidorylaimus sp., Prodorylaimus mas	3e-153	95.16		Omnivore
R2_SV_139	Enoplida	Alaimidae	Alaimus	Alaimus sp. PDL-2005	6e-170	98		Bacteria feeder
R2_SV_549	Enoplida	Trischistomatidae	Trischistoma	Trischistoma sp.	8e-174	98.58		Predator
R2_SV_517	Rhabditida	Aphelenchoididae	Aphelenchoides	Aphelenchoides sp. RH-2018	6e-170	98.28		Plant feeder
R2_SV_176	Rhabditida	Cephalobidae	Acrobeloides	Acrobeloides thornei	1e-172	98.84		Bacteria feeder
R2_SV_281	Rhabditida	Cephalobidae	Acrobeloides	Acrobeloides thornei isolate I17_SMIRACONTROL33	2e-170	98.55		Bacteria feeder
R2_SV_114	Rhabditida	Homungellidae	Perodira	Perodira minuta	2e-164	97.41		Parasite
R2_SV_135	Rhabditida	Homungellidae	Perodira	Perodira minuta	5e-161	96.83		Parasite
R2_SV_145	Rhabditida	Pratylenchidae	Pratylenchus	Pratylenchus penetrans	2e-164	97.14		Plant feeder
R2_SV_209	Rhabditida	Pratylenchidae	Pratylenchus	Pratylenchus penetrans	3e-173	98.57		Plant feeder
R2_SV_243	Rhabditida	Pratylenchidae	Pratylenchus	Pratylenchus penetrans	3e-173	98.58		Plant feeder

R2_SV_265	Rhabditida	Pratylenchidae	Pratylenchus	Pratylenchus penetrans strain PratPen2	6e-175	98.86		Plant feeder
R2_SV_273	Rhabditida	Pratylenchidae	Pratylenchus	Pratylenchus penetrans	6e-175	98.86		Plant feeder
R2_SV_279	Rhabditida	Pratylenchidae	Pratylenchus	Pratylenchus penetrans isolate ILVO-Pp	6e-175	98.86		Plant feeder
R2_SV_301	Rhabditida	Pratylenchidae	Pratylenchus	Pratylenchus penetrans strain PratPen2	6e-175	98.86		Plant feeder
R2_SV_304	Rhabditida	Pratylenchidae	Pratylenchus	Pratylenchus penetrans	1e-171	98.29		Plant feeder
R2_SV_315	Rhabditida	Pratylenchidae	Pratylenchus	Pratylenchus penetrans	6e-175	98.86		Plant feeder
R2_SV_337	Rhabditida	Pratylenchidae	Pratylenchus	Pratylenchus penetrans	6e-175	98.86		Plant feeder
R2_SV_369	Rhabditida	Pratylenchidae	Pratylenchus	Pratylenchus penetrans strain PratPen2	3e-173	98.57		Plant feeder
R2_SV_410	Rhabditida	Pratylenchidae	Pratylenchus	Pratylenchus penetrans	6e-175	98.86		Plant feeder
R2_SV_414	Rhabditida	Pratylenchidae	Pratylenchus	Pratylenchus penetrans strain PratPen2	6e-175	98.86		Plant feeder
R2_SV_415	Rhabditida	Pratylenchidae	Pratylenchus	Pratylenchus penetrans	3e-173	98.58		Plant feeder
R2_SV_433	Rhabditida	Pratylenchidae	Pratylenchus	Pratylenchus penetrans strain PratPen2	6e-175	98.86		Plant feeder
R2_SV_519	Rhabditida	Pratylenchidae	Pratylenchus	Pratylenchus penetrans isolate ILVO-Pp	3e-173	98.58		Plant feeder
R2_SV_563	Rhabditida	Pratylenchidae	Pratylenchus	Pratylenchus penetrans	3e-173	98.56		Plant feeder
R2_SV_12	Rhabditida	Travassosinematidae	Travassosinema	Travassosinema sp.	1e-171	98.29		Parasite
R2_SV_47	Rhabditida	Travassosinematidae	Travassosinema	Travassosinema sp. Nago	1e-171	98.29		Parasite
R2_SV_132	Rhabditida	Travassosinematidae	Travassosinema	Travassosinema sp. Ishigaki	6e-170	98		Parasite
R2_SV_180	Rhabditida	Travassosinematidae	Travassosinema	Travassosinema sp. Ishigaki	6e-170	98		Parasite
R2_SV_222	Rhabditida	Travassosinematidae	Travassosinema	Travassosinema sp. Ishigaki	1e-166	97.43		Parasite
R2_SV_246	Rhabditida	Travassosinematidae	Travassosinema	Travassosinema sp. Ishigaki	1e-166	97.43		Parasite
R2_SV_282	Rhabditida	Travassosinematidae	Travassosinema	Travassosinema sp. Ishigaki	3e-168	97.71		Parasite
R2_SV_448	Rhabditida	Tylenchidae	Basiria	Basiria gracilis isolate CA1	1e-166	96.93		Plant feeder
R2_SV_26	Rhabditida	Tylenchidae	Basiria	Basiria sp.	4e-162	96.84		Plant feeder
R2_SV_291	Rhabditida	Tylenchidae	Boleodorus	Boleodorus thylactus	1e-171	98.29		Plant feeder
R2_SV_186	Rhabditida	Tylenchidae	Filenchus	Filenchus discrepans	2e-134	92.31		Fungus feeder
R2_SV_217	Rhabditida	Tylenchidae	Filenchus	Filenchus misellus*	8e-174	98.58	98.86	Fungus feeder
R2_SV_329	Rhabditida	Tylenchidae	Filenchus	Filenchus misellus*	1e-166	97.44	97.72	Fungus feeder
R2_SV_343	Rhabditida	Tylenchidae	Filenchus	Ottolenchus longiurus strain FileLon2	1e-136	92.59		Fungus feeder
R2_SV_452	Rhabditida	Tylenchidae	Filenchus	Filenchus discrepans strain FileDis2	5e-141	93.43		Fungus feeder
R2_SV_483	Rhabditida	Tylenchidae	Filenchus	Filenchus discrepans strain FileDis2	2e-144	93.97		Fungus feeder
R2_SV_558	Rhabditida	Tylenchidae	Filenchus	Filenchus discrepans strain FileDis2*	2e-144	94	94.29	Fungus feeder
R2_SV_584	Rhabditida	Tylenchidae	Filenchus	Filenchus discrepans strain FileDis2	1e-142	93.71		Fungus feeder
R2_SV_336	Rhabditida	Tylenchidae	Miculenchus	Miculenchus salmae*	2e-85	83.91	95.68	Plant feeder
R2_SV_450	Rhabditida	Tylenchidae	Miculenchus	Miculenchus salvus*	2e-129	91.43	91.76	Plant feeder
R2_SV_18	Rhabditida	Ungellidae	Drasico	Drasico nemoralis	1e-157	96.24		Parasite
R2_SV_594	Rhabditida	Ungellidae	Drasico	Drasico nemoralis	2e-79	83		Parasite
R2_SV_116	Triplonchida	Diphtherophoridae	Diphtherophora	Diphtherophora obesus*	2e-119	89.63	98.56	Fungus feeder
R2_SV_275	Triplonchida	Tripylidae	Tripylella	Tripylella sp. 1031	4e-162	96.85		Predator
R2_SV_390	Triplonchida	Prismatolaimidae	Prismatolaimus	Prismatolaimus cf. intermedius *	6e-165	97.41	98.85	Bacteria feeder
R2_SV_470	Triplonchida	Prismatolaimidae	Prismatolaimus	Prismatolaimus cf. intermedius	2e-119	89.91		Bacteria feeder
R3_SV_11	Enoplida	Alaimidae	Alaimus	Alaimus sp. SSU1_19	5e-140	97.95		Bacteria feeder
R3_SV_72	Enoplida	Alaimidae	Alaimus	Alaimus sp. PDL-2005	1e-141	98.29		Bacteria feeder
R3_SV_88	Enoplida	Alaimidae	Alaimus	Alaimus sp. SSU1_19	5e-135	96.93		Bacteria feeder
R3_SV_103	Enoplida	Trischistomatidae	Trischistoma	Trischistoma sp.	1e-136	97.21		Predator
R3_SV_148	Enoplida	Trischistomatidae	Trischistoma	Trischistoma sp.	5e-140	97.95		Predator
R3_SV_153	Enoplida	Trischistomatidae	Trischistoma	Trischistoma sp.	7e-119	93.81		Predator
R3_SV_158	Enoplida	Trischistomatidae	Trischistoma	Trischistoma sp.	2e-143	98.63		Predator
R3_SV_192	Monhysterida	Monhysteridae	Geomonhystera	Geomonhystera sp.	7e-144	98.97		Bacteria feeder
R3_SV_48	Rhabditida	Aphelenchoididae	Aphelenchoides	Aphelenchoides sp.	7e-144	98.47		Plant feeder
R3_SV_123	Rhabditida	Aphelenchoididae	Aphelenchoides	Aphelenchoides sp.	3e-142	98.63		Plant feeder
R3_SV_132	Rhabditida	Ecphyadophoridae	Lelenchus	Lelenchus sp.	5e-135	97.24		Plant feeder
R3_SV_28	Rhabditida	Tylenchidae, Merliniidae	Atetylenchus, Geocenamus, Psilenchus	Atetylenchus sp., Geocenamus chengi, Psilenchus sp.	7e-144	98.97		Plant feeder
R4_SV_76	Chromadorida	Cyatholaimidae	Achromadora	Achromadora cf terricola JH-2004*	3e-148	96.6	98.46	Eucaryote feeder
R4_SV_251	Enoplida	Trischistomatidae	Trischistoma	Trischistoma sp.	6e-155	98.12		Predator
R4_SV_296	Monhysterida	Monhysteridae	Eumonhystera	Eumonhystera filiformis*	2e-155	98.13	98.75	Bacteria feeder
R4_SV_108	Rhabditida	Aphelenchoididae	Aphelenchoides	Aphelenchoides sp.	7e-134	94.39		Plant feeder
R4_SV_133	Rhabditida	Aphelenchoididae	Aphelenchoides	Aphelenchoides sp.	2e-135	94.67		Plant feeder
R4_SV_70	Rhabditida	Cephalobidae	Eucephalobus	Eucephalobus striatus isolate EuceStr2*	2e-155	98.13	98.44	Bacteria feeder
R4_SV_287	Rhabditida	Ecphyadophoridae	Lelenchus	Lelenchus sp. MB-2019	9e-148	97.15		Plant feeder
R4_SV_246	Rhabditida	Meloidogynidae	Meloidogyne	Meloidogyne ichinohei isolate MeloIch3*	5e-111	90.06	96.54	Plant feeder
R4_SV_222	Rhabditida	Panagrolaimidae	Propanagrolaimus	Propanagrolaimus sp.	3e-73	83.18		Bacteria feeder
R4_SV_45	Rhabditida	Tylenchidae	Discopersicus, Basiria	Discopersicus iranicus, Basiria sp.	2e-154	98.12		Plant feeder
R4_SV_30	Rhabditida	Ungellidae	Drasico	Drasico nemoralis	1e-151	97.52		Parasite
R4_SV_57	Rhabditida	Ungellidae	Drasico	Drasico nemoralis	6e-150	97.2		Parasite
R4_SV_168	Rhabditida	Ungellidae	Drasico	Drasico nemoralis	1e-116	91.19		Parasite
R4_SV_121	Triplonchida	Diphtherophoridae	Diphtherophora	Diphtherophora sp. Shahrood*	2e-129	93.21	95.21	Fungus feeder
R4_SV_273	Triplonchida	Tripylidae	Tripylella	Tripylella sp. 1031	7e-149	96.88		Predator

[a]Regional nematode sequence variants (SVs) from four regions that were identified in amplicons from field, copse, and house garden samples.

[b]The top hit of species from BLASTN searches and their genus, family, order and e-value are indicated. The top hit sequences that did not have any taxonomic data such as environmental samples were omitted and the second-closest species for the respective SVs are shown instead with an asterisk. %id (hit) and %id (top) indicate the percentage of nucleotide sequence identity for the SV to the hit species and top hit sequences (including environmental samples), respectively.

[c]Feeding types were predicted as described in the legens for S3 Table.
