# Supplementary material for: Distinct community structures of soil nematodes from three ecologically different sites revealed by high-throughput amplicon sequencing of four 18S ribosomal RNA gene regions
Source: PLoS One. 2021 Apr 15;16(4):e0249571. doi: 10.1371/journal.pone.0249571 (PMC8049254; doi:10.1371/journal.pone.0249571)
Supplement: S8 Table — (PDF) [file pone.0249571.s008.pdf]

S8 Table. Regional nematode SVs shared with high sequence similarities.

R1_SV		R2_SV		R2_SV (continued)		R3_SV		R4_SV	
R1_SV_3	R1_SV_32 (A/G, T/G)[a]	R2_SV_1	R2_SV_105 (A/G); R2_SV_52 (C/T, A/T)	R2_SV_296		R3_SV_1	R3_SV_43 (A/G)	R4_SV_1	R4_SV_18 (T/A)
R1_SV_4		R2_SV_3		R2_SV_299		R3_SV_2	R3_SV_25 (T/C)	R4_SV_2	
R1_SV_5	R1_SV_100 (A/G)	R2_SV_5		R2_SV_300		R3_SV_4	R3_SV_34 (G/A); R3_SV_87 (A/G); R3_SV_92 (A/G)	R4_SV_7	
R1_SV_10		R2_SV_10	R2_SV_131 (G/A)	R2_SV_301	R2_SV_247 (G/A, T/C); R2_SV_433 (G/A, G/A)	R3_SV_5	R3_SV_51 (T/G); R3_SV_141 (G/A)	R4_SV_12	
R1_SV_12	R1_SV_169 (A/T); R1_SV_70 (T/C); R1_SV_326 (C/G, T/C)	R2_SV_12	R2_SV_132 (G/A); R2_SV_180 (G/A); R2_SV_282 (T/G, T/G); R2_SV_47 (C/T)	R2_SV_303	R2_SV_19 (G/T, G/T)	R3_SV_7		R4_SV_14	
R1_SV_14		R2_SV_15		R2_SV_304		R3_SV_10		R4_SV_15	R4_SV_117 (C/T)
R1_SV_22	R1_SV_123 (T/C, C/T); R1_SV_200 (T/G); R1_SV_310 (C/T)	R2_SV_17		R2_SV_315	R2_SV_209 (T/G)	R3_SV_11		R4_SV_17	
R1_SV_23	R1_SV_67 (G/A)	R2_SV_18		R2_SV_317		R3_SV_12	R3_SV_86 (T/G)	R4_SV_18	R4_SV_1 (A/T)
R1_SV_24	R1_SV_57 (C/G)	R2_SV_19	R2_SV_205 (G/A); R2_SV_303 (T/G, T/G)	R2_SV_324		R3_SV_13		R4_SV_24	
R1_SV_27		R2_SV_21		R2_SV_329		R3_SV_14	R3_SV_68 (T/G)	R4_SV_25	
R1_SV_30		R2_SV_24	R2_SV_334 (T/G, T/G)	R2_SV_334	R2_SV_24 (G/T, G/T)	R3_SV_15	R3_SV_54 (A/G)	R4_SV_26	R4_SV_150 (G/A); R4_SV_158 (C/G, G/A)
R1_SV_32[b]	R1_SV_32 (G/A, G/T)	R2_SV_25	R2_SV_355 (T/G, T/G)	R2_SV_336		R3_SV_17		R4_SV_27	
R1_SV_33	R1_SV_121 (C/T)	R2_SV_26		R2_SV_337		R3_SV_19		R4_SV_30	R4_SV_57 (C/T)
R1_SV_34	R1_SV_194 (C/A)	R2_SV_30		R2_SV_338		R3_SV_22		R4_SV_31	
R1_SV_36		R2_SV_31	R2_SV_363 (A/G, C/T)	R2_SV_343		R3_SV_25	R3_SV_2 (C/T)	R4_SV_33	
R1_SV_37		R2_SV_35	R2_SV_427 (A/G, A/G)	R2_SV_349	R2_SV_214 (C/A, T/G); R2_SV_253 (C/T, T/G)	R3_SV_26		R4_SV_34	
R1_SV_41	R1_SV_106 (C/T, G/A)	R2_SV_36		R2_SV_351		R3_SV_27		R4_SV_36	
R1_SV_43		R2_SV_38		R2_SV_355	R2_SV_25 (G/T, G/T)	R3_SV_28		R4_SV_37	
R1_SV_44	R1_SV_124 (A/G)	R2_SV_41		R2_SV_362	R2_SV_531 (C/T, C/T)	R3_SV_29	R3_SV_36 (T/A); R3_SV_100 (T/A, T/C)	R4_SV_38	
R1_SV_48	R1_SV_233 (T/G, A/G); R1_SV_413 (T/G, C/A)	R2_SV_42		R2_SV_363	R2_SV_31 (G/A, T/C)	R3_SV_30		R4_SV_39	
R1_SV_54		R2_SV_47	R2_SV_12 (T/C); R2_SV_132 (G/A, T/C); R2_SV_180 (T/C, G/A)	R2_SV_369		R3_SV_32	R3_SV_36 (C/T, T/G); R3_SV_100 (T/G)	R4_SV_41	
R1_SV_57	R1_SV_24 (G/C)	R2_SV_48		R2_SV_371		R3_SV_34	R3_SV_4 (A/G); R3_SV_87 (A/G, A/G); R3_SV_92 (A/G, A/G)	R4_SV_42	R4_SV_103 (C/T, G/A)
R1_SV_62		R2_SV_52	R2_SV_1 (T/C, T/A)	R2_SV_386	R2_SV_89 (G/A, T/C)	R3_SV_36	R3_SV_29 (A/T); R3_SV_32 (T/C, G/T); R3_SV_100 (T/C)	R4_SV_43	
R1_SV_67	R1_SV_23 (A/G)	R2_SV_53		R2_SV_390	R2_SV_418 (T/C)	R3_SV_39		R4_SV_45	
R1_SV_69		R2_SV_54		R2_SV_404		R3_SV_40		R4_SV_47	
R1_SV_70	R1_SV_12 (C/T); R1_SV_169 (C/T, A/T); R1_SV_326 (C/G)	R2_SV_56		R2_SV_410		R3_SV_42		R4_SV_57	R4_SV_30 (T/C)
R1_SV_80		R2_SV_63		R2_SV_411	R2_SV_214 (T/A, G/T); R2_SV_253 (G/T, G/A); R2_SV_127 (T/G, G/T)	R3_SV_43	R3_SV_1 (G/A)	R4_SV_58	
R1_SV_81		R2_SV_72		R2_SV_414		R3_SV_44	R3_SV_48 (A/G)	R4_SV_59	R4_SV_62 (G/A)
R1_SV_84	R1_SV_295 (C/T); R1_SV_455 (T/C)	R2_SV_89	R2_SV_386 (A/G, C/T)	R2_SV_415		R3_SV_45	R3_SV_104 (C/T); R3_SV_113 (G/A)	R4_SV_62	R4_SV_59 (A/G)
R1_SV_87	R1-SV_418 (A/T, C/T)	R2_SV_90		R2_SV_418	R2_SV_390 (C/T)	R3_SV_47		R4_SV_63	
R1_SV_100	R1_SV_5 (A/G	R2_SV_95		R2_SV_427	R2_SV_35 (G/A, G/A)	R3_SV_48	R3_SV_44 (G/A)	R4_SV_70	
R1_SV_104	R1_SV_170 (C/T)	R2_SV_102	R2_SV_142 (C/T)	R2_SV_430	R2_SV_147 (T/C, insC)[c]	R3_SV_51	R3_SV_5 (G/T); R3_SV_141 (G/A, G/T)	R4_SV_73	
R1_SV_106	R1_SV_41 (T/C, G/A)	R2_SV_103		R2_SV_433	R2_SV_301 (A/G, A/G); R2_SV_247 (T/C, A/G)	R3_SV_53	R3_SV_65 (A/C)	R4_SV_76	R4_SV_118 (A/T, A/C)
R1_SV_110		R2_SV_105	R2_SV_1 (G/A)	R2_SV_443		R3_SV_54	R3_SV_15 (G/A)	R4_SV_86	R4_SV_93 (G/A)
R1_SV_116	R1_SV_188 (AG/GT)	R2_SV_110	R2_SV_164 (C/T)	R2_SV_448		R3_SV_58		R4_SV_87	R4_SV_175 (T/C, C/T)
R1_SV_119	R1_SV_184 (C/T, T/C)	R2_SV_114	R2_SV_135 (TG/CA)	R2_SV_450		R3_SV_65	R3_SV_53 (C/A)	R4_SV_93	R4_SV_86 (A/G)
R1_SV_121	R1_SV_33 (T/C)	R2_SV_116		R2_SV_452		R3_SV_68	R3_SV_14 (G/T)	R4_SV_97	
R1_SV_123	R1_SV_22 (C/T, T/C); R1_SV_310 (C/T)	R2_SV_120	R2_SV_128 (T/C); R2_SV_279 (A/G, G/A); R2_SV_259 (G/A, T/C); R2_SV_127 (C/G); R2_SV_411 (C/T, T/G); R2_SV_253 (C/T, G/A)	R2_SV_461		R3_SV_69		R4_SV_103	R4_SV_42 (T/C, A/G)
R1_SV_124	R1_SV_44 (G/A)	R2_SV_127	R2_SV_120 (G/C, C/T); R2_SV_214 (G/T, T/A); R2_SV_411 (G/T, T/G); R2_SV_253 (G/T, G/A)	R2_SV_470		R3_SV_72		R4_SV_108	R4_SV_133 (G/T, C/T)
R1_SV_132		R2_SV_128	R2_SV_120 (C/T); R2_SV_279 (A/G, C/T); R2_SV_259 (G/A, T/C); R2_SV_253 (C/T, T/C)	R2_SV_471		R3_SV_83		R4_SV_111	R4_SV_161 (T/C)
R1_SV_134		R2_SV_129		R2_SV_476		R3_SV_84		R4_SV_112	
R1_SV_152		R2_SV_131	R2_SV_10 (A/G)	R2_SV_483		R3_SV_86	R3_SV_12 (G/T)	R4_SV_117	R4_SV_15 (T/C)
R1_SV_169	R1_SV_12 (T/A); R1_SV_70 (T/C, T/A)	R2_SV_132	R2_SV_12 (A/G); R2_SV_222 (G/A, C/T); R2_SV_180 (A/G, G/A); R2_SV_47 (A/G, C/T)	R2_SV_490		R3_SV_87	R3_SV_4 (G/A); R3_SV_34 (G/A, G/A); R3_SV_92 (A/G, A/G)	R4_SV_118	R4_SV_76 (T/A, C/A)
R1_SV_170	R1_SV_104 (T/C)	R2_SV_134		R2_SV_499		R3_SV_88		R4_SV_121	
R1_SV_171		R2_SV_135	R2_SV_114 (CA/TG)	R2_SV_500		R3_SV_89		R4_SV_129	
R1_SV_178		R2_SV_139		R2_SV_505		R3_SV_90		R4_SV_133	R4_SV_108 (T/G, T/C)
R1_SV_180		R2_SV_140		R2_SV_517		R3_SV_92	R3_SV_4 (G/A); R3_SV_34 (G/A, G/A); R3_SV_87 (G/A, A/G)	R4_SV_149	
R1_SV_184	R1_SV_119 (T/C, C/T)	R2_SV_142	R2_SV_102 (T/C)	R2_SV_519		R3_SV_95		R4_SV_150	R4_SV_26 (A/G); R4_SV_158 (C/G)
R1_SV_188	R1_SV_116 (GT/AG)	R2_SV_145		R2_SV_531	R2_SV_362 (T/C, T/C)	R3_SV_98		R4_SV_158	R4_SV_26 (G/C, A/G); R4_150 (G/C)
R1_SV_194	R1_SV_34 (A/C)	R2_SV_147	R2_SV_430 (C/T, delC)[c]	R2_SV_549		R3_SV_100	R3_SV_29 (A/C, C/T); R3_SV_32 (G/T); R3_SV_36 (C/T)	R4_SV_159	
R1_SV_200	R1_SV_22 (G/T); R1_SV_310 (C/T, G/T)	R2_SV_150		R2_SV_558		R3_SV_103		R4_SV_161	R4_SV_111 (C/T)
R1_SV_205		R2_SV_164	R2_SV_110 (T/C)	R2_SV_559		R3_SV_104	R3_SV_45 (T/C); R3_SV_113 (G/A)	R4_SV_168	
R1_SV_217		R2_SV_168		R2_SV_563		R3_SV_107		R4_SV_170	
R1_SV_221		R2_SV_176		R2_SV_584		R3_SV_113	R3_SV_45 (A/G); R3_SV_104 (C/T)	R4_SV_175	R4_SV_87 (C/T, T/C)
R1_SV_231		R2_SV_179		R2_SV_594		R3_SV_122		R4_SV_182	
R1_SV_233	R1_SV_48 (G/T, G/A); R1_SV_413 (C/A, G/A)	R2_SV_180	R2_SV_12 (A/G); R2_SV_132 (G/A, A/G);R2_SV_47 (C/T, A/G)			R3_SV_123		R4_SV_184	

R1_SV_291		R2_SV_186				R3_SV_126		R4_SV_186	
R1_SV_293		R2_SV_190	R2_SV_281 (T/C, A/G)			R3_SV_129		R4_SV_208	
R1_SV_295	R1_SV_84 (T/C); R1_SV_455 (T/C)	R2_SV_198				R3_SV_132		R4_SV_215	
R1_SV_298		R2_SV_199				R3_SV_141	R3_SV_5 (A/G); R3_SV_51 (A/G, T/G)	R4_SV_219	
R1_SV_304		R2_SV_200				R3_SV_144		R4_SV_222	
R1_SV_310	R1_SV_22 (T/C); R1_SV_123 (T/C, T/G); R1_SV_200 (T/C)	R2_SV_202				R3_SV_148		R4_SV_233	
R1_SV_312		R2_SV_205	R2_SV_19 (A/G)			R3_SV_149		R4_SV_235	
R1_SV_316		R2_SV_208				R3_SV_151		R4_SV_237	
R1_SV_322		R2_SV_209	R2_SV_315 (G/T)			R3_SV_153		R4_SV_246	
R1_SV_326	R1_SV_12 (G/C, C/T); R1_SV_70 (G/C)	R2_SV_214	R2_SV_253 (A/T, G/A); R2_SV_127 (T/G, A/T); R2_SV_411 (T/G); R2_SV_349 (A/C, G/T)			R3_SV_158		R4_SV_251	
R1_SV_351		R2_SV_215	R2_SV_272 (C/T, C/T)			R3_SV_192		R4_SV_255	
R1_SV_353		R2_SV_217						R4_SV_273	
R1_SV_371		R2_SV_222	R2_SV_132 (A/G, T/C)					R4_SV_287	
R1_SV_386		R2_SV_237						R4_SV_293	
R1_SV_396		R2_SV_238						R4_SV_296	
R1_SV_400		R2_SV_243						R4_SV_326	
R1_SV_413	R1_SV_48 (G/T, A/C); R1_SV_233 (A/C, A/G)	R2_SV_246						R4_SV_335	
R1_SV_418	R1_SV_87 (T/A, T/C)	R2_SV_247	R2_SV_301 (A/G, C/T); R2_SV_433 (C/T, G/A)					R4_SV_338	
R1_SV_424		R2_SV_253	R2_SV_127 (T/G, A/G); R2_SV_214 (T/A, A/G); R2_SV_411 (T/G, A/G)					R4_SV_360	
R1_SV_437		R2_SV_259	R2_SV_128 (A/G, C/T)					R4_SV_372	
R1_SV_439		R2_SV_260							
R1_SV_455	R1_SV_84 (C/T); R1_SV_295 (C/T, C/T)	R2_SV_262							
R1_SV_476		R2_SV_265							
R1_SV_493		R2_SV_270							
R1_SV_507		R2_SV_272	R2_SV_215 (T/C, T/C)						
R1_SV_521		R2_SV_273							
R1_SV_543		R2_SV_275							
R1_SV_551		R2_SV_279	R2_SV_120 (G/A, A/G)						
R1_SV_556		R2_SV_281	R2_SV_190 (C/T, G/A)						
R1_SV_562		R2_SV_282	R2_SV_12 (G/T, G/T)						
R1_SV_578		R2_SV_288							
R1_SV_594		R2_SV_291							

Regional nematode SVs that were highly similar to other SVs were screened using ATGC software as described in the Materials and methods section. The nematode SVs containing less than 2 diffrent sites are indicated.

[a]Differing nucleotide sequences are indicated in parentheses. In this case, R1_SV_3 and R1_SV_32 share two different sites in their nucleotide sequences: SV_3 contains A and T, whereas the corresponding sites of the SV_32 sequence contain G and G, respectively.

[b]The higher number of nematode SVs of a pair or group of highly similar SVs are indicated in yellow except for the SVs with the lowest number.

[c]del: deletion; ins: insertion
