# Supplementary material for: Distinct community structures of soil nematodes from three ecologically different sites revealed by high-throughput amplicon sequencing of four 18S ribosomal RNA gene regions
Source: PLoS One. 2021 Apr 15;16(4):e0249571. doi: 10.1371/journal.pone.0249571 (PMC8049254; doi:10.1371/journal.pone.0249571)
Supplement: S9 Table — (PDF) [file pone.0249571.s009.pdf]

S9 Table. Regional nematode SVs identical to the previously determined [28] gene-derived operational taxonomic units (rOTUs) found using the copse-derived nematodes (Z01).

rOTU name	Abundance %	Order	Region 1	Region 2	Region 3	Region 4
Z01rOTU01	19.1	Rhabditida	R1_SV_23; R1_SV_67 (R1a)	R2_SV_19	R3_SV_14	R4_SV_25
Z01rOTU02	16.2	Dorylaimida	R1_SV_44; R1_SV_124 (R1a)	R2_SV_30	R3_SV_29	R4_SV_47
Z01rOTU03	16.2	Dorylaimida	R1_SV_351		R3_SV_2	R4_SV_338
Z01rOTU04	10.3	Dorylaimida	R1_SV_132	R2_SV_200; R2_SV_238 (R2a)	R3_SV_36[a]	R4_SV_182 (R4a)
Z01rOTU05	5.9	Triplonchida	R1_SV_437			
Z01rOTU06	4.4	Rhabditida	R1_SV_312	R2_SV_150; R2_SV_291 (R2a)	R3_SV_69	R4_SV_149
Z01rOTU07	4.4	Dorylaimida	R1_SV_5	R2_SV_10	R3_SV_5	R4_SV_7
Z01rOTU08	2.9	Dorylaimida	R1_SV_69	R2_SV_102	R3_SV_36[a]	R4_SV_63
Z01rOTU09	2.9	Triplonchida		R2_SV_363		
Z01rOTU10	2.9	Rhabditida	R1_SV_33	R2_SV_21	R3_SV_12	R4_SV_24
Z01rOTU11	2.9	Rhabditida	R1_SV_178	R2_SV_95	R3_SV_58	R4_SV_112
Z01rOTU12	2.9	Triplonchida		R2_SV_53	R3_SV_27	R4_SV_42
Z01rOTU13[a]	1.5	Dorylaimida			R3_SV_36[a]	
Z01rOTU14	1.5	Triplonchida				
Z01rOTU15	1.5	Plectida		R2_SV_36	R3_SV_7	R4_SV_31
Z01rOTU16	1.5	Triplonchida		R2_SV_31	R3_SV_39	R4_SV_37
Z01rOTU17	1.5	Triplonchida	R1_SV_221	R2_SV_300		R4_SV_237
Z01rOTU18	1.5	Triplonchida			R3_SV_129	

Regional nematode SVs identical to SSU-derived operational taxonomic units (rOTUs) were screened using ATGC software as described in the Materials and methods section. The name of the rOTUs, their relative abundance in the total nematodes, and their orders are also shown. The SVs identical to polymorphic rOTUs are indicated with parenthesis describing the polymorphic sequence. Z01 represents the soil sample code the experimental ID. [a]Since Z01rOTU04, 08 and 13 share the same sequence in region 3 and any SVs in other regions were not detected, Z01rOTU13 is likely undetected and omitted from the detected Z01rOTUs in this study.
