# Supplementary material for: Distinct community structures of soil nematodes from three ecologically different sites revealed by high-throughput amplicon sequencing of four 18S ribosomal RNA gene regions
Source: PLoS One. 2021 Apr 15;16(4):e0249571. doi: 10.1371/journal.pone.0249571 (PMC8049254; doi:10.1371/journal.pone.0249571)
Supplement: S10 Table — (PDF) [file pone.0249571.s010.pdf]

S10 Table. Nematode SVs in regions 1 and 2 identical to the previously determined [26] rOTUs using the flowerbed (K01)- and cultivated field (H01)-derived nematodes.

rOTU name	Abundance %	Order	Region 1	Region 2
K01rOTU01	26.5	Dorylaimida		
K01rOTU02a	26.5	Mononchida	R1_SV_32 (288bp)	R2_SV_25 (299bp)
K01rOTU02b	8.8	Mononchida		
K01rOTU03	8.8	Triplonchida	R1_SV_304 (282bp)	R2_SV_275 (299bp)
K01rOTU04	5.9	Rhabditida		
K01rOTU05	4.4	Rhabditida	R1_SV_33 (280bp)	R2_SV_48 (297bp)
K01rOTU06	4.4	Enoplida		
K01rOTU07	2.9	Rhabditida	R1_SV_43 (279bp)	R2_SV_56 (297bp)
K01rOTU08	1.5	Dorylaimida		
K01rOTU09	1.5	Plectida		
K01rOTU10	1.5	Dorylaimida	R1_SV_5 (284bp)	R2_SV_10 (301bp)
K01rOTU11	1.5	Dorylaimida	R1_SV_36 (286bp)	R2_SV_35 (301bp)
K01rOTU12	1.5	Dorylaimida	R1_SV_351 (291bp)	
K01rOTU13	1.5	Araeolaimida	R1_SV_217 (283bp)	
K01rOTU14	1.5	Dorylaimida		
K01rOTU15	1.5	Triplonchida	R1_SV_110 (283bp)	R2_SV_116 (298bp)
H01rOTU01	16.7	Rhabditida	R1_SV_291 (283bp)	R2_SV_215 (300bp); R2_SV_272 (300bp)
H01rOTU02	12.5	Rhabditida		
H01rOTU03	12.5	Rhabditida	R1_SV_33 (280bp)	R2_SV_21 (297bp)
H01rOTU04	10.4	Rhabditida	R1_SV_33 (280bp)	R2_SV_48 (297bp)
H01rOTU05	10.4	Rhabditida		
H01rOTU06	10.4	Plectida		R2_SV_36 (299bp)
H01rOTU07	6.3	Rhabditida		
H01rOTU08	4.2	Plectida	R1_SV_10 (283bp)	R2_SV_17 (299bp); R2_SV_476 (299bp)
H01rOTU09	4.2	Mononchida		
H01rOTU10	4.2	Triplonchida	R1_SV_14 (282bp)	R2_SV_31 (298bp)
H01rOTU11	4.2	Rhabditida		R2_SV_1 (295bp)
H01rOTU12	2.1	Dorylaimida		
H01rOTU13	2.1	Dorylaimida	R1_SV_37 (284bp)	R2_SV_41 (301bp)

Nematode SVs in regions 1 and 2 which are identical to SSU-derived operational taxonomic units (rOTUs) were screened using ATGC software as described in the Materials and methods section. The name of the rOTUs, their relative abundance in the total nematodes, and their orders are also shown. The length of the identical nucleotide sequence overlapping the corresponding rOTU is indicated in parenthesis. K01 and H01 represent the soil sample codes and experimental IDs.
