# Supplementary material for: Distinct community structures of soil nematodes from three ecologically different sites revealed by high-throughput amplicon sequencing of four 18S ribosomal RNA gene regions
Source: PLoS One. 2021 Apr 15;16(4):e0249571. doi: 10.1371/journal.pone.0249571 (PMC8049254; doi:10.1371/journal.pone.0249571)
Supplement: S11 Table — (PDF) [file pone.0249571.s011.pdf]

S11 Table. Regional nematode SVs, feeding types, and colonizer–persister (cp)-values in families in the four 18S small subunit ribosomal RNA (SSU) regions.

Family	Region 1	Region 2	Region 3	Region 4	Order	Feeding types	cp-value
Actinolaimidae	0	1	0	0	Dorylaimida	Omnivore	5
Alaimidae	6	4	3	2	Enoplida	Bacteria feeder	4
Aphelenchidae	0	5	2	4	Rhabditida	Fungus feeder	2
Aphelenchoididae	1	1	3	4	Rhabditida	Fungus feeder	2
Aporcelaimidae	2	3	1	1	Dorylaimida	Omnivore	5
Belondiridae	5	3	2	1	Dorylaimida	Plant feeder	5
Cephalobidae	8	7	4	6	Rhabditida	Bacteria feeder	2
Comesomatidae	1	0	0	0	Araeolaimida	NA	-
Criconematidae	3	3	3	2	Rhabditida	Plant feeder	3
Cyatholaimidae	11	6	2	5	Chromadorida	Omnivore	3
Diphtherophoridae	3	4	0	3	Triplonchida	Fungus feeder	3
Dorylaimidae	1	1	0	3	Dorylaimida	Omnivore	4
Ecphyadophoridae	1	0	1	1	Rhabditida	Plant feeder	2
Homungellidae	0	2	0	0	Rhabditida	Parasite	-
Hoplolaimidae	1	1	0	1	Rhabditida	Plant feeder	3
Leptonchidae	0	1	0	0	Dorylaimida	Fungus feeder	4
Longidoridae	0	0	1	0	Dorylaimida	Plant feeder	5
Meloidogynidae	2	3	1	5	Rhabditida	Plant feeder	3
Microlaimidae	0	5	0	0	Desmodorida	Bacteria feeder	2
Monhysteridae	0	1	1	2	Monhysterida	Bacteria feeder	2
Mononchidae	0	0	0	1	Mononchida	Predator	4
Mydonomidae	0	2	0	0	Dorylaimida	Omnivore	4
Mylonchulidae	5	4	2	2	Mononchida	Predator	4
Nygolaimidae	1	1	1	1	Dorylaimida	Predator	5
Odontolaimidae	0	0	1	0	Triplonchida	Bacteria feeder	3
Panagrolaimidae	0	0	0	1	Rhabditida	Bacteria feeder	1
Plectidae	3	4	1	2	Plectida	Bacteria feeder	2
Pratylenchidae	3	34	6	2	Rhabditida	Plant feeder	3
Prismatolaimidae	3	6	1	3	Triplonchida	Bacteria feeder	3
Qudsianematidae	3	2	2	3	Dorylaimida	Omnivore	4
Rhabditidae	2	2	6	2	Rhabditida	Bacteria feeder	1
Thelastomatidae	4	0	1	0	Rhabditida	Parasite	-
Travassosinematidae	0	7	0	0	Rhabditida	Parasite	-
Trichodoridae	1	0	1	0	Triplonchida	Plant feeder	4
Tripylidae	1	1	1	1	Triplonchida	Predator	3
Trischistomatidae	2	2	5	2	Enoplida	Predator	3
Tylenchidae	8	18	4	5	Rhabditida	Plant feeder	2
Tylencholaimellidae	0	0	0	1	Dorylaimida	Omnivore	4
Tylencholaimidae	0	2	0	2	Dorylaimida	Fungus feeder	4
Ungellidae	3	2	1	3	Rhabditida	Parasite	-
NA	4	6	10	5			
Total nematode SVs	88	144	67	76			

Numbers of regional nematode SVs in families found in the four SSU regions studied are shown with their corresponding orders, feeding types, and cp-values. The SVs with more than two families were classfied as NA (not assigned). The feeding types and cp-values of families were obtained as described in the Materials and methods section. A hyphen (-) indicates no data.
