# Supplementary figures and images for: Distinct community structures of soil nematodes from three ecologically different sites revealed by high-throughput amplicon sequencing of four 18S ribosomal RNA gene regions
Source: PLoS One. 2021 Apr 15;16(4):e0249571. doi: 10.1371/journal.pone.0249571 (PMC8049254; doi:10.1371/journal.pone.0249571)

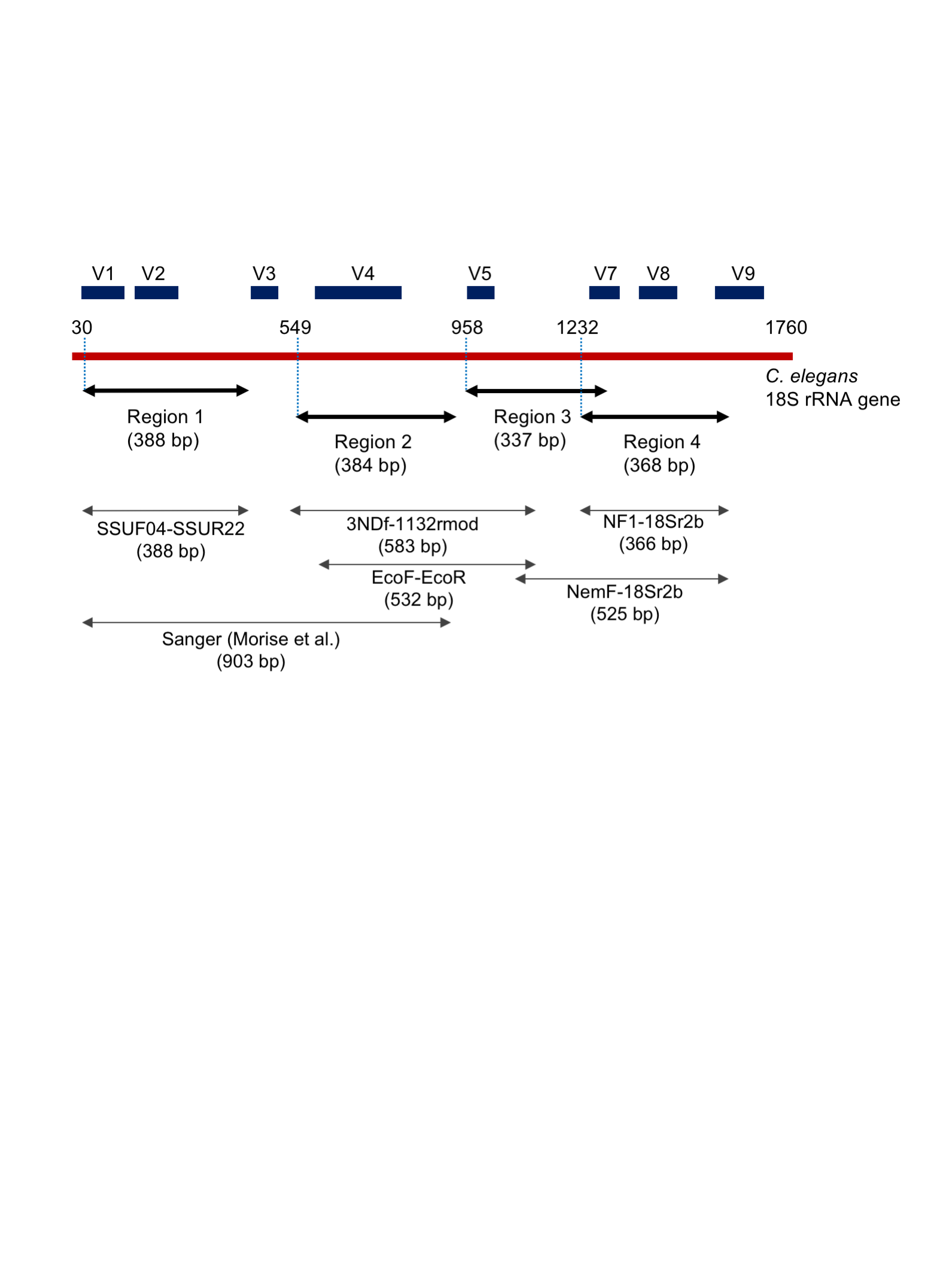

Supplement: S1 Fig — The four barcode regions for PCR amplifications (regions 1–4, with amplicon sizes shown) used in this and in a prior study [28] are indicated by black double-headed arrows. The numbers indicating the nucleotide positions of the 5’-end of forward primers are shown on the entire SSU gene prepared from the nucleotide sequence of the C. elegans ribosomal RNA gene cluster (X03680). The dark blue boxes correspond to the hypervariable regions of the eukaryotic SSU genes reported by Hugerth et al. [38] and Hadziavdic et al. [37]. The regions that were amplified by the four indicated published primer sets (SSUF04-SSUR22 [39], EcoF-EcoR [32], NF1-18Sr2b [29], and NemF-18Sr2b [30]), as well as the amplified region from prior Sanger-based DNA barcoding [26], are indicated using gray double-headed arrows with amplicon sizes in parenthesis. (TIFF) [file pone.0249571.s012.tiff]

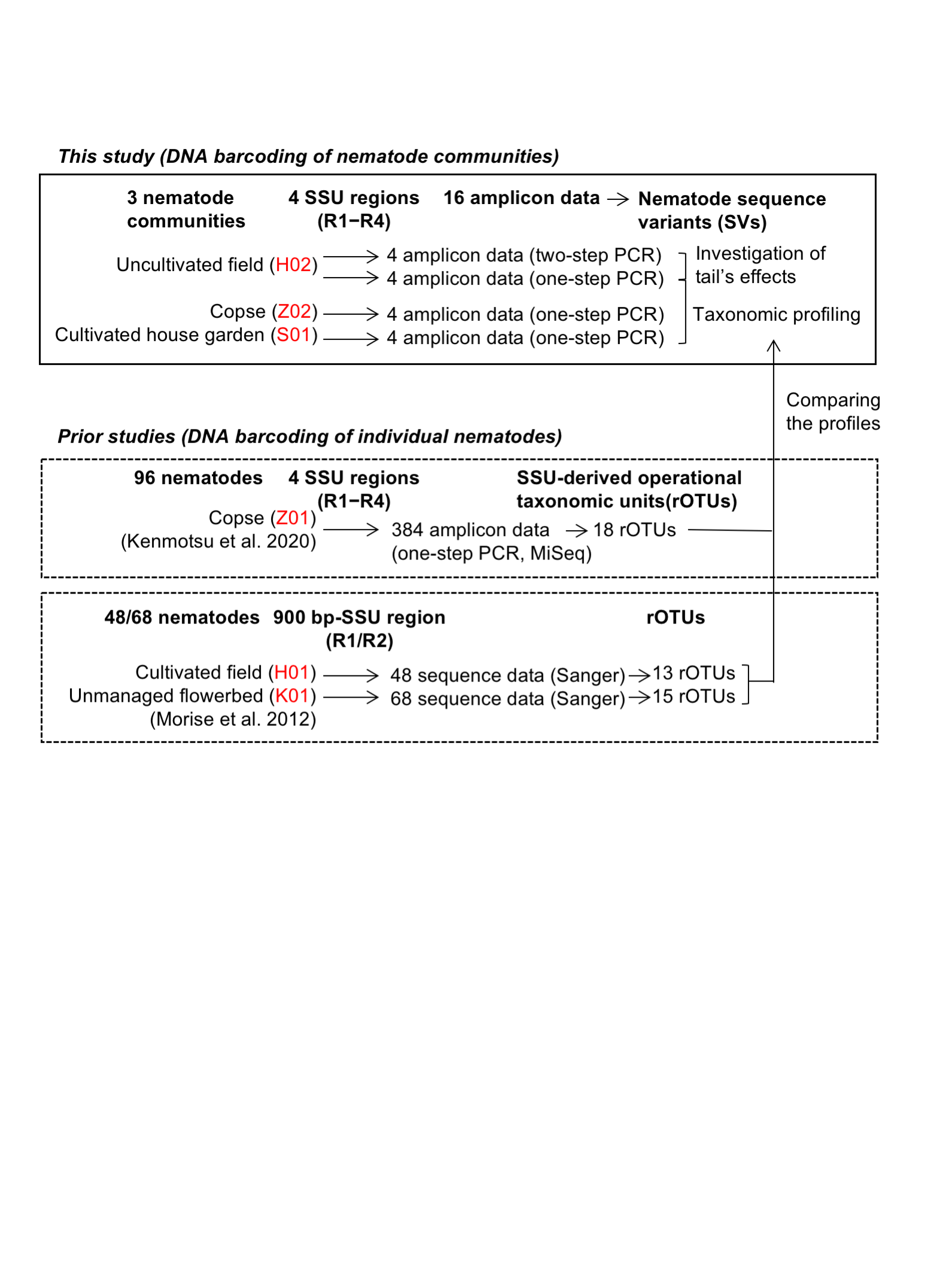

Supplement: S2 Fig — The experimental scheme is shown in a box by solid line. The resultant nematode-derived SVs identified in this study were used for taxonomic profiling and investigation of primer tails’s effects on amplifications. The SSU-derived operational taxonomic units (rOTUs) identified in prior MiSeq [28]- and Sanger [26]-based DNA barcoding of individual nematodes are shown in boxes (broken lines) and were integrated into the cladograms of regional nematode SVs to compare the taxomic profiles (see Figs 3–6). Red colored codes in parenthesis (e.g., H02) represent the soil sample code (H: field soil) and the experimental ID (ID: 02). (TIFF) [file pone.0249571.s013.tiff]

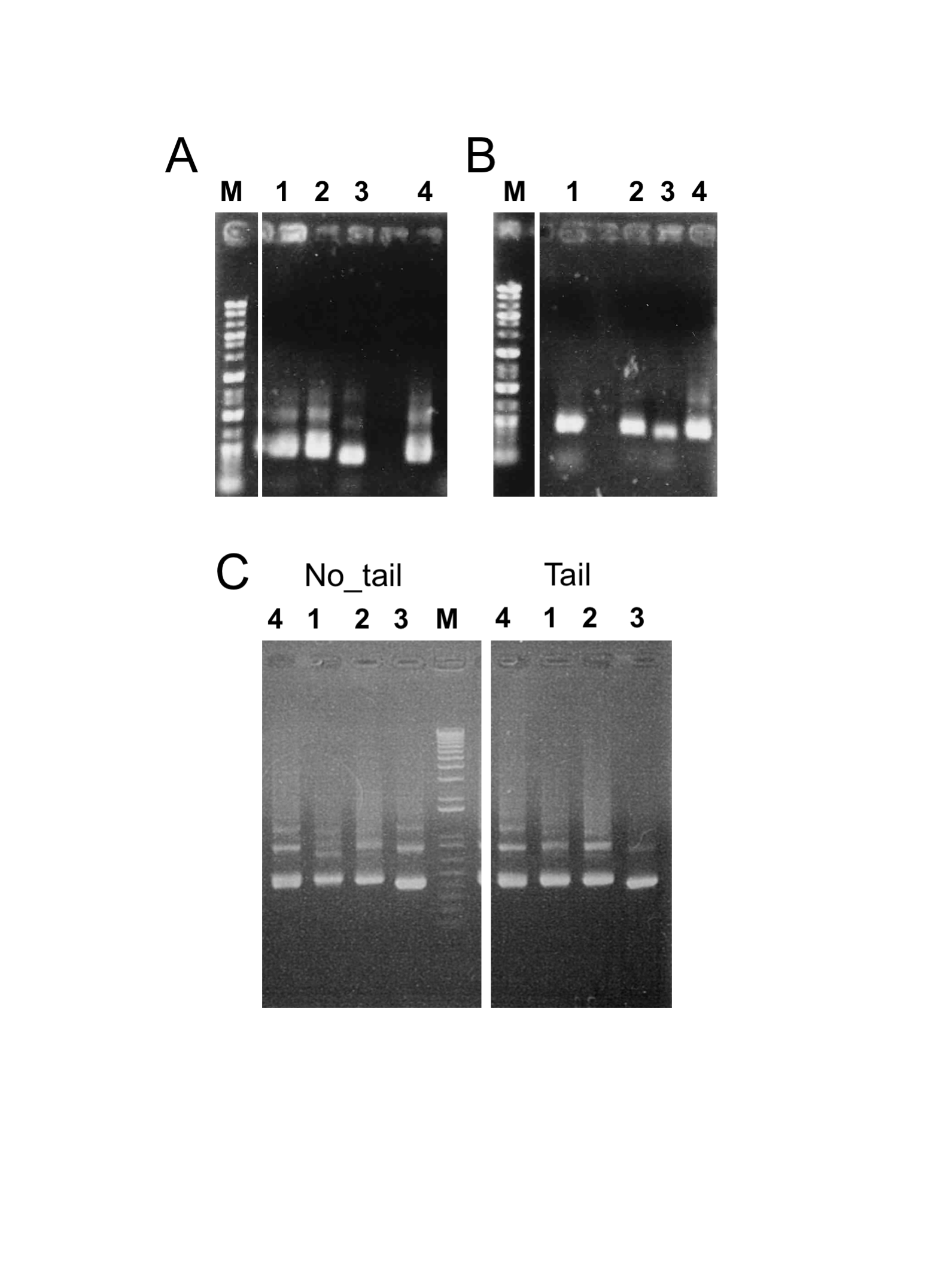

Supplement: S3 Fig — Five microliter aliquots of each reaction mixture containing nematode DNA from the (A) copse, (B) house garden and (C) field soils were subjected to 1% (A and B) or 2% (C) agarose gel electrophoresis. The PCR products from region 1–4 in the gels are visualized using successive ethidium bromide staining and are shown in lanes 1–4, respectively. In the field sample, the products prepared from one-step PCR with tailed (Tail) and two-step PCR with tailless and tailed primers (No_tail) were analyzed. M: Gene Ladder Wide 1 (Nippon Gene, Toyama, Japan) as a size marker. (TIFF) [file pone.0249571.s014.tiff]

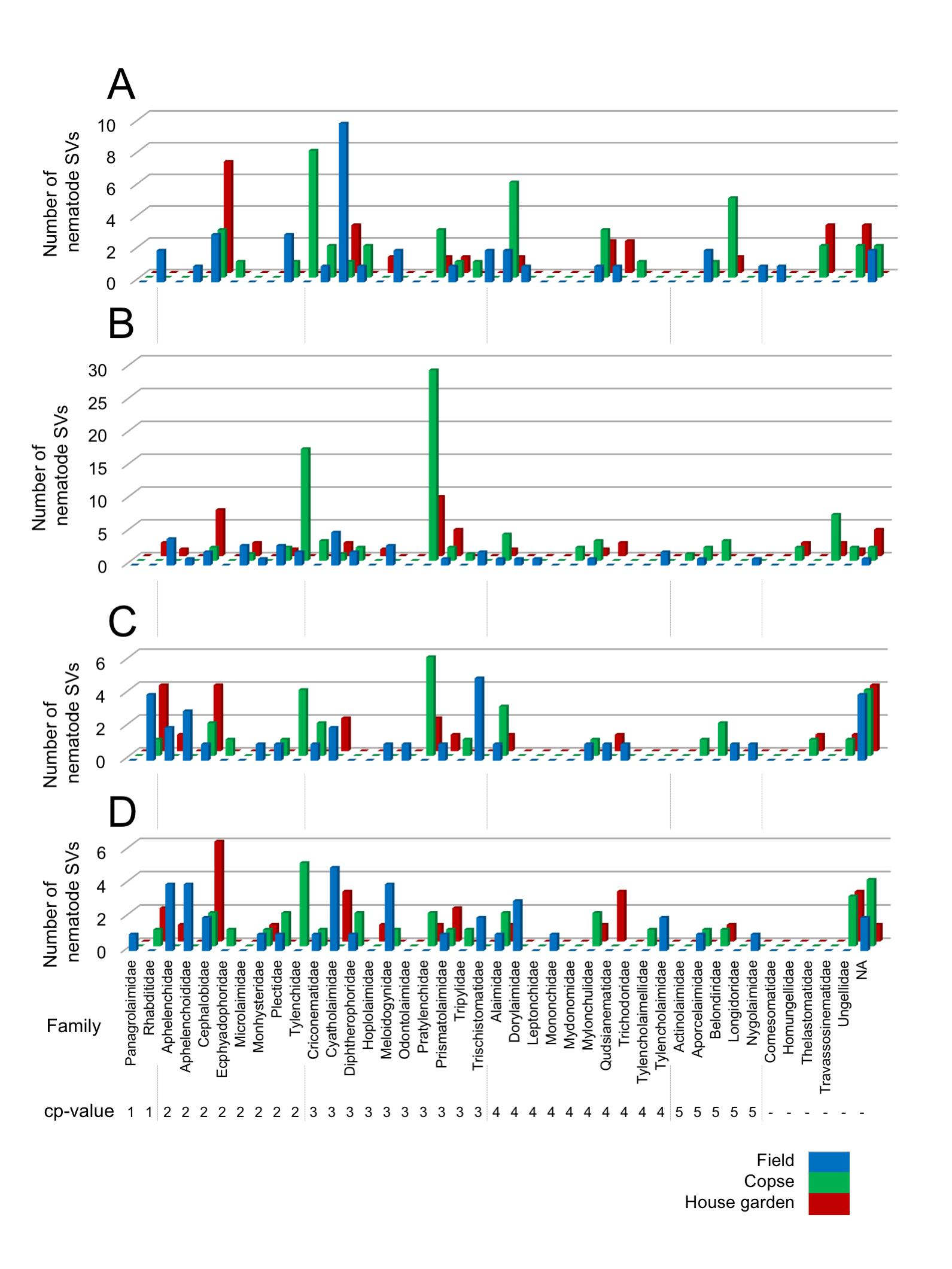

Supplement: S4 Fig — The number of nematode SVs identified from the field (blue), copse (green), and house garden (dark red) soil samples is indicated by 3D-histogram for regions (A–D) 1–4, respectively, in each family. The cp-values, shown at the bottom of (D), indicate the nematode’s life strategy characteristics as described in the Materials and methods section. NA: not assigned to a single family (i.e., assigned to multiple families). (TIFF) [file pone.0249571.s015.tiff]

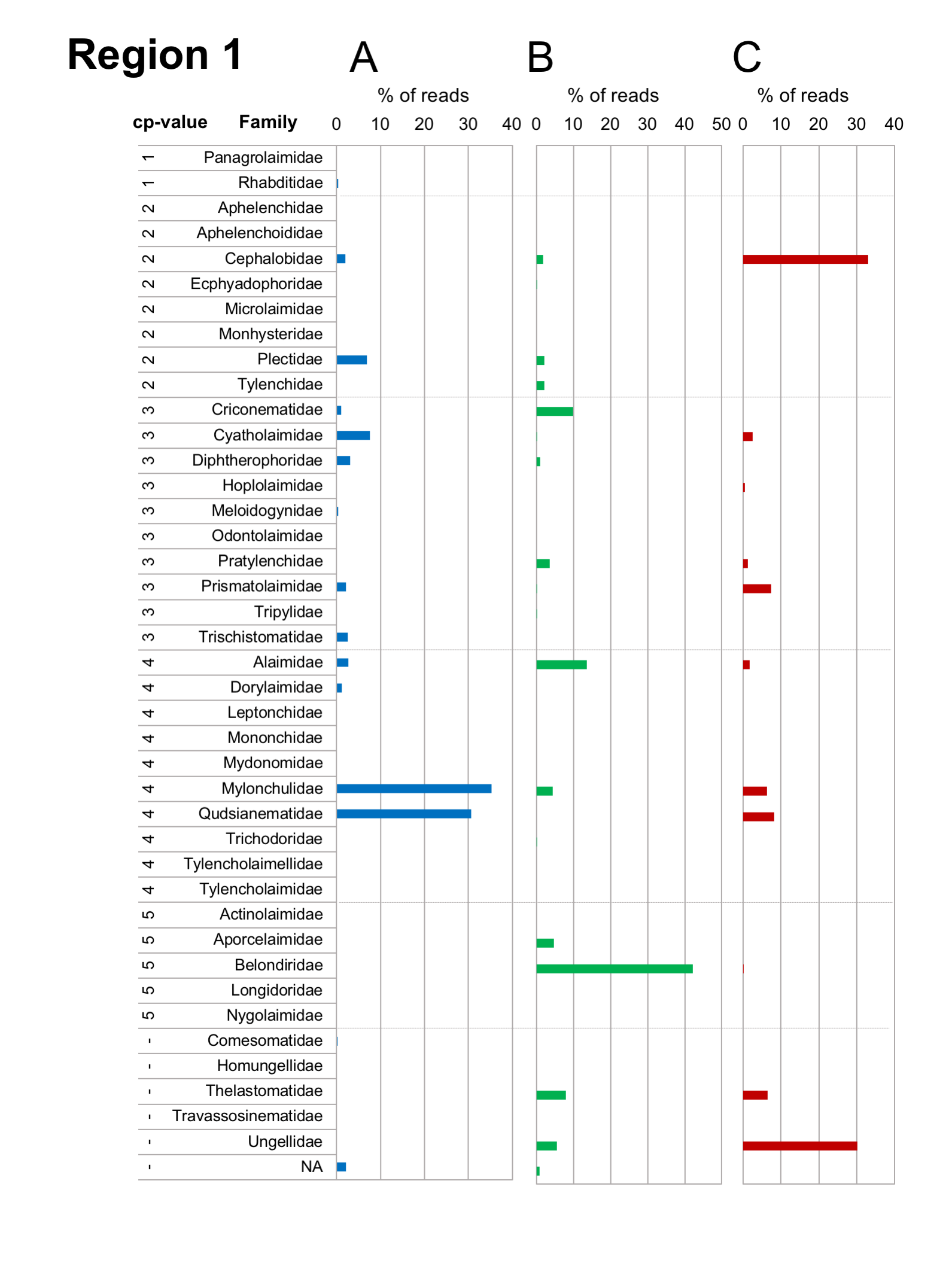

Supplement: S5 Fig — The relative abundance (%) of sequence reads of SVs in region 1 from the (A) field, (B) copse, and (C) house garden samples for each family. Families are aligned by their cp-values (1–5); undefined cp-values are indicated by a hyphen (-). NA: not assigned to a single family. (TIFF) [file pone.0249571.s016.tiff]

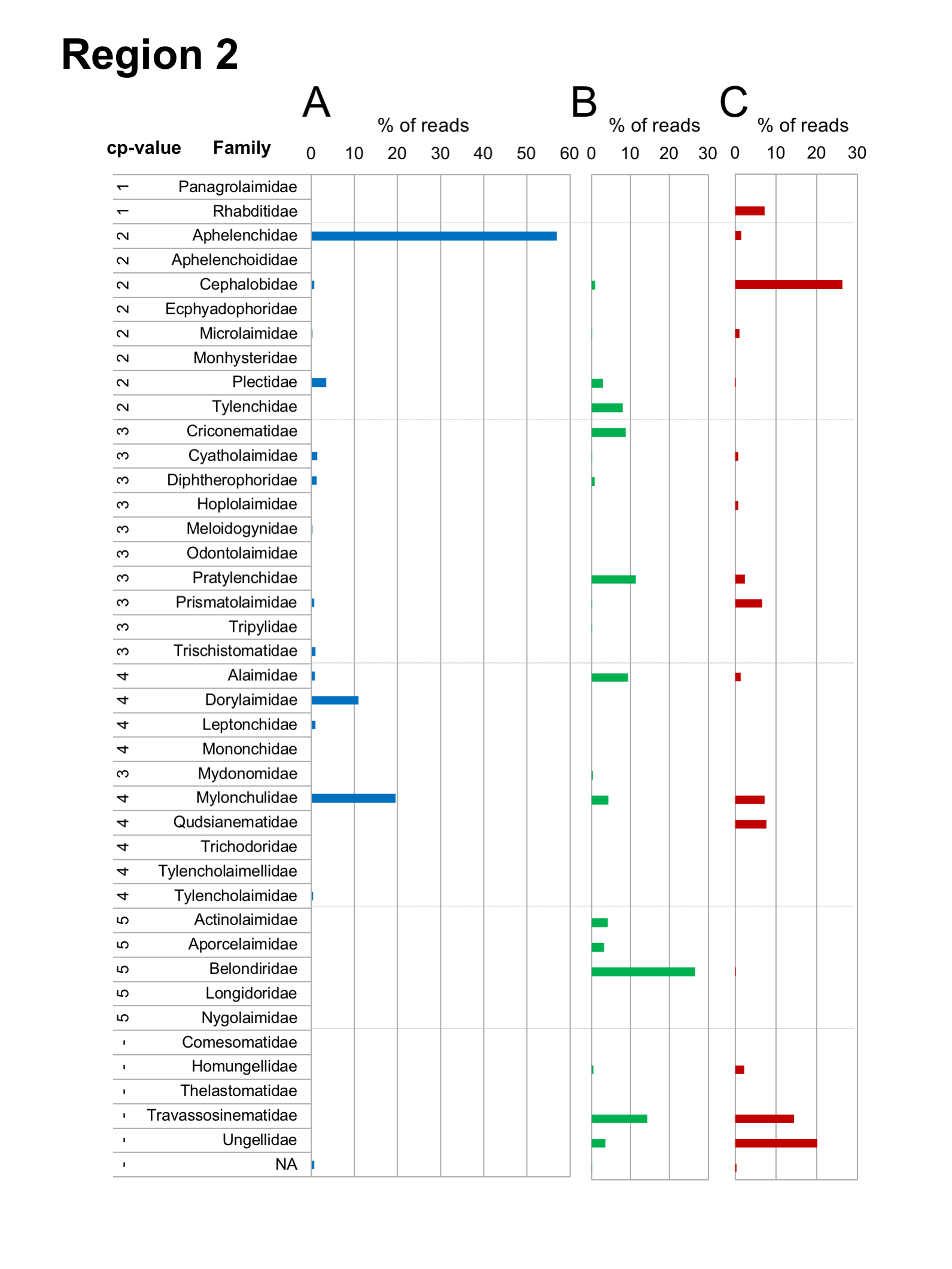

Supplement: S6 Fig — The relative abundance (%) of sequence reads of SVs in region 2 from the (A) field, (B) copse, and (C) house garden samples for each family. Families are aligned by their cp-values (1–5); undefined cp-values are indicated by a hyphen (-). NA: not assigned to a single family. (TIFF) [file pone.0249571.s017.tiff]

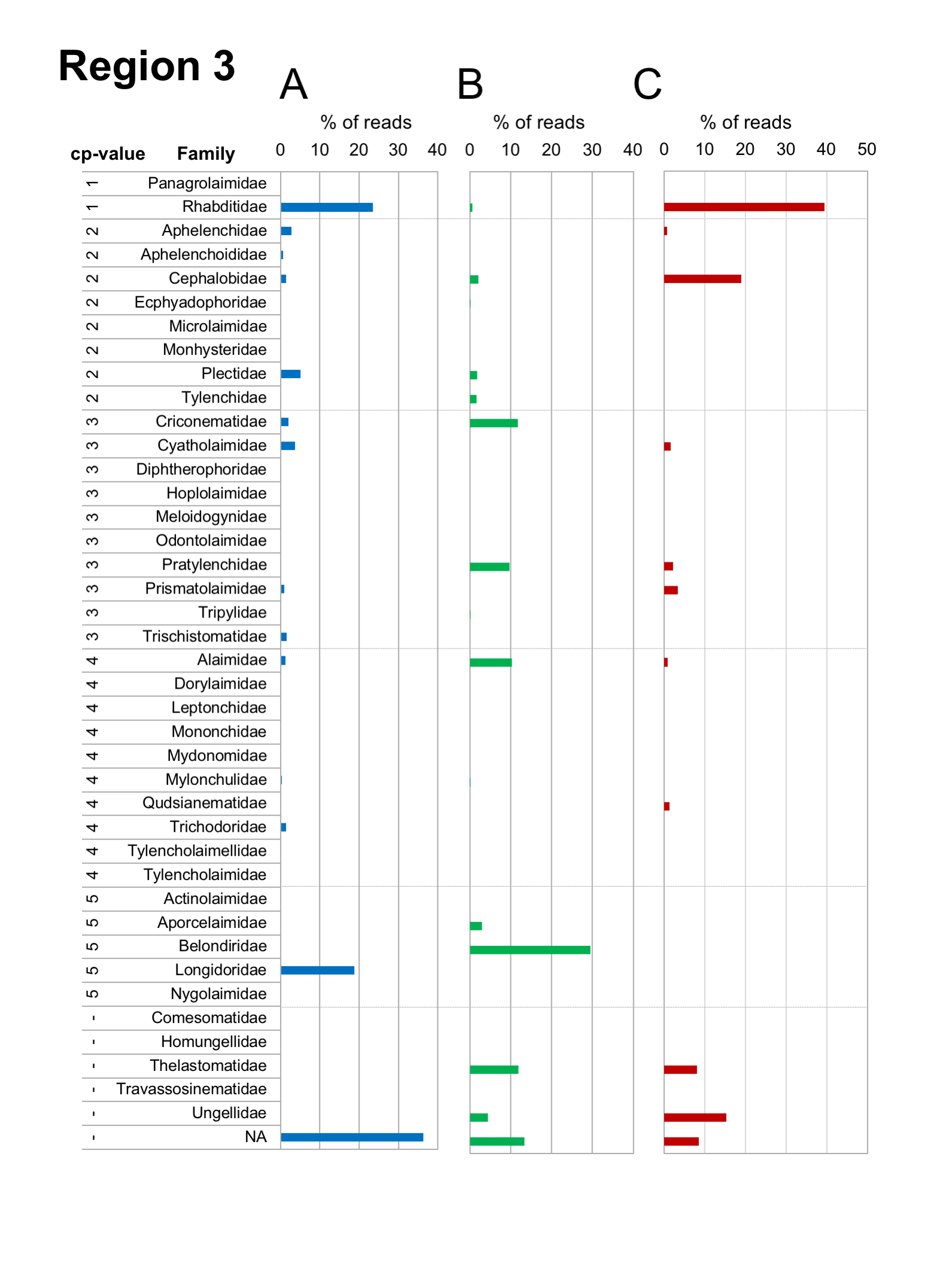

Supplement: S7 Fig — The relative abundance (%) of sequence reads of SVs in region 3 from the (A) field, (B) copse, and (C) house garden samples for each family. Families are aligned by their cp-values (1–5); undefined cp-values are indicated by a hyphen (-). NA: not assigned to a single family. (TIFF) [file pone.0249571.s018.tiff]

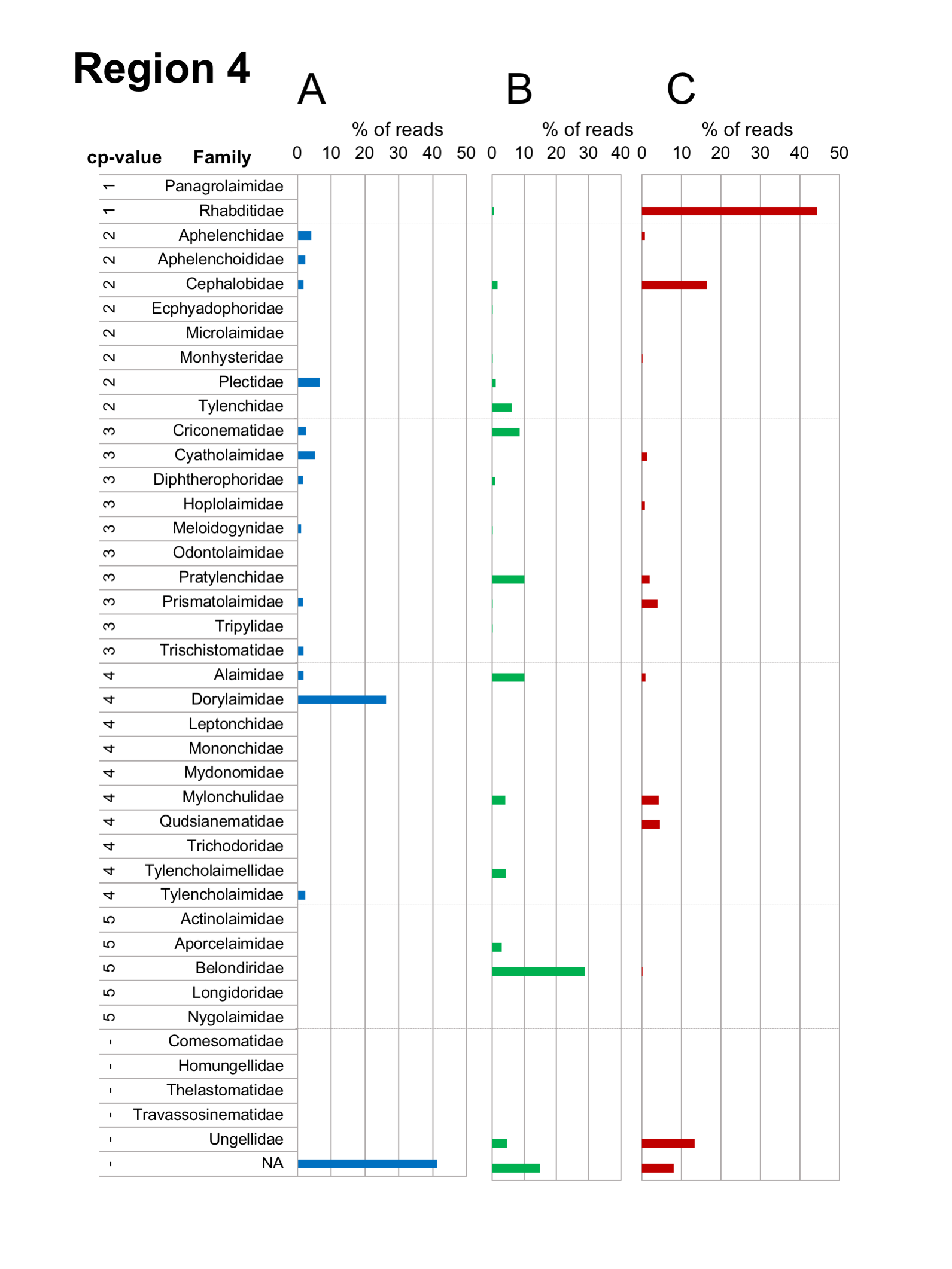

Supplement: S8 Fig — The relative abundance (%) of sequence reads of SVs in region 4 from the (A) field, (B) copse, and (C) house garden samples for each family. Families are aligned by their cp-values (1–5); undefined cp-values are indicated by a hyphen (-). NA: not assigned to a single family. (TIFF) [file pone.0249571.s019.tiff]

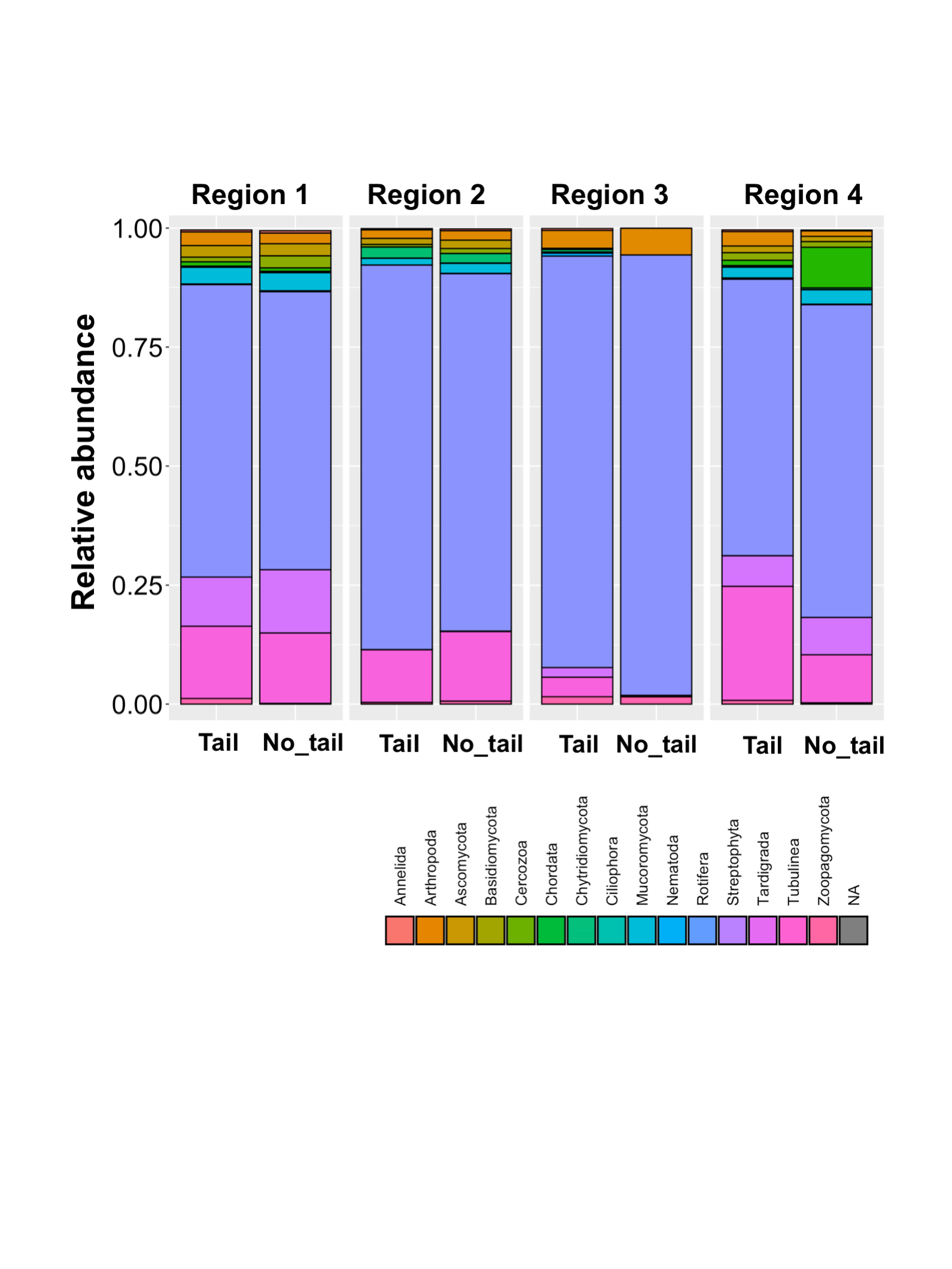

Supplement: S9 Fig — The SVs from each SSU gene region shown on top of the histograms were identified from the high-throughput sequencing of amplicons prepared from one-step PCR with tailed primers (Tail) and two-step PCR with tailless and tailed primers (No_tail). The relative abundance of SVs and their phylum are shown, where the phylum is indicated by the colors in the legend. NA: Not assigned. (TIFF) [file pone.0249571.s020.tiff]

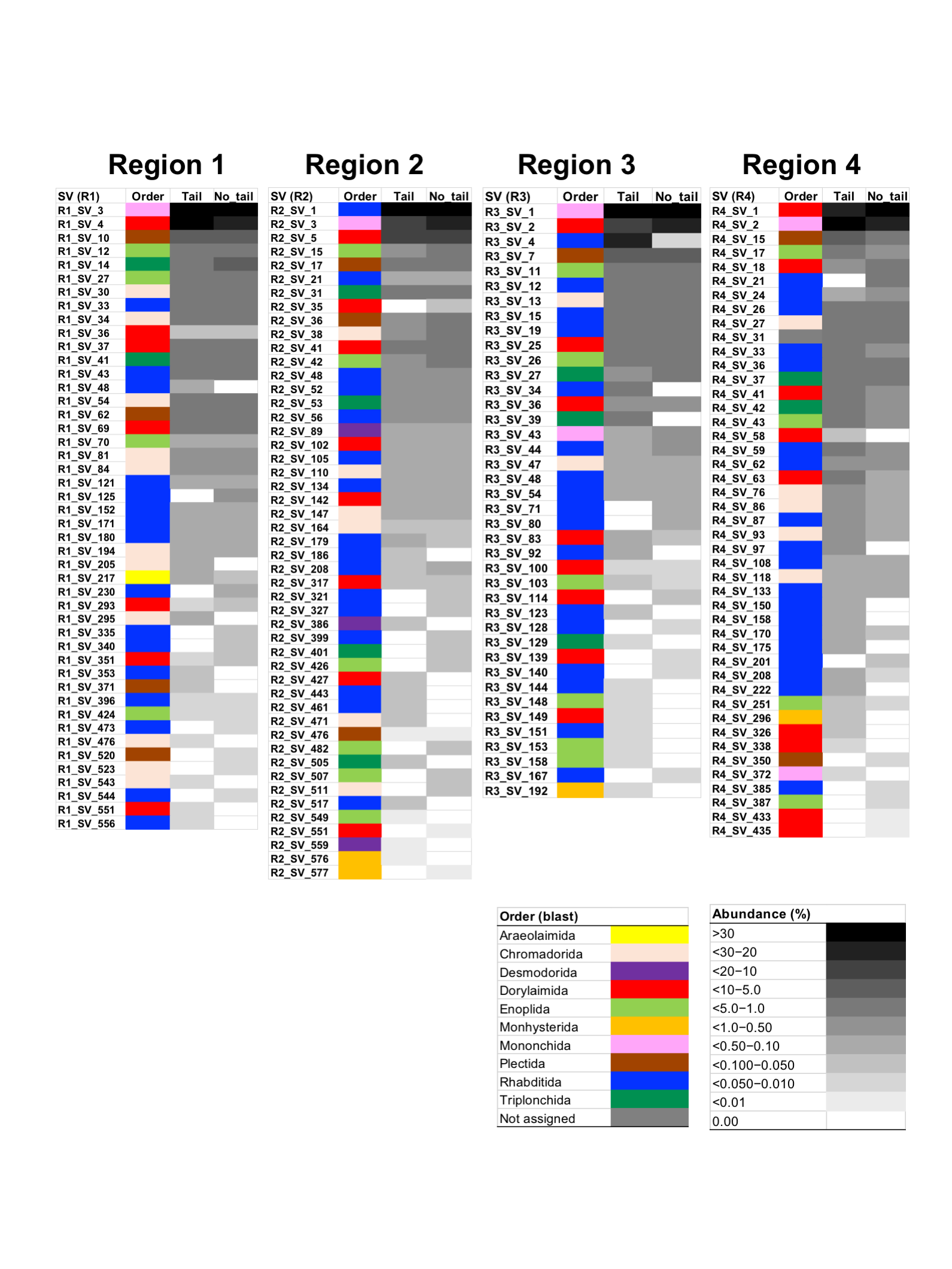

Supplement: S10 Fig — The regional nematode SVs are indicated to the left of the column with each SSU gene region on top and their relative abundance of sequence reads in the amplicons from one-step PCR (Tail) and two-step PCR (No_tail) are indicated in each SSU gene region by density bars as shown in the legend. The colors represent the orders of the SVs determined by BLASTN search as indicated in the legend. (TIFF) [file pone.0249571.s021.tiff]

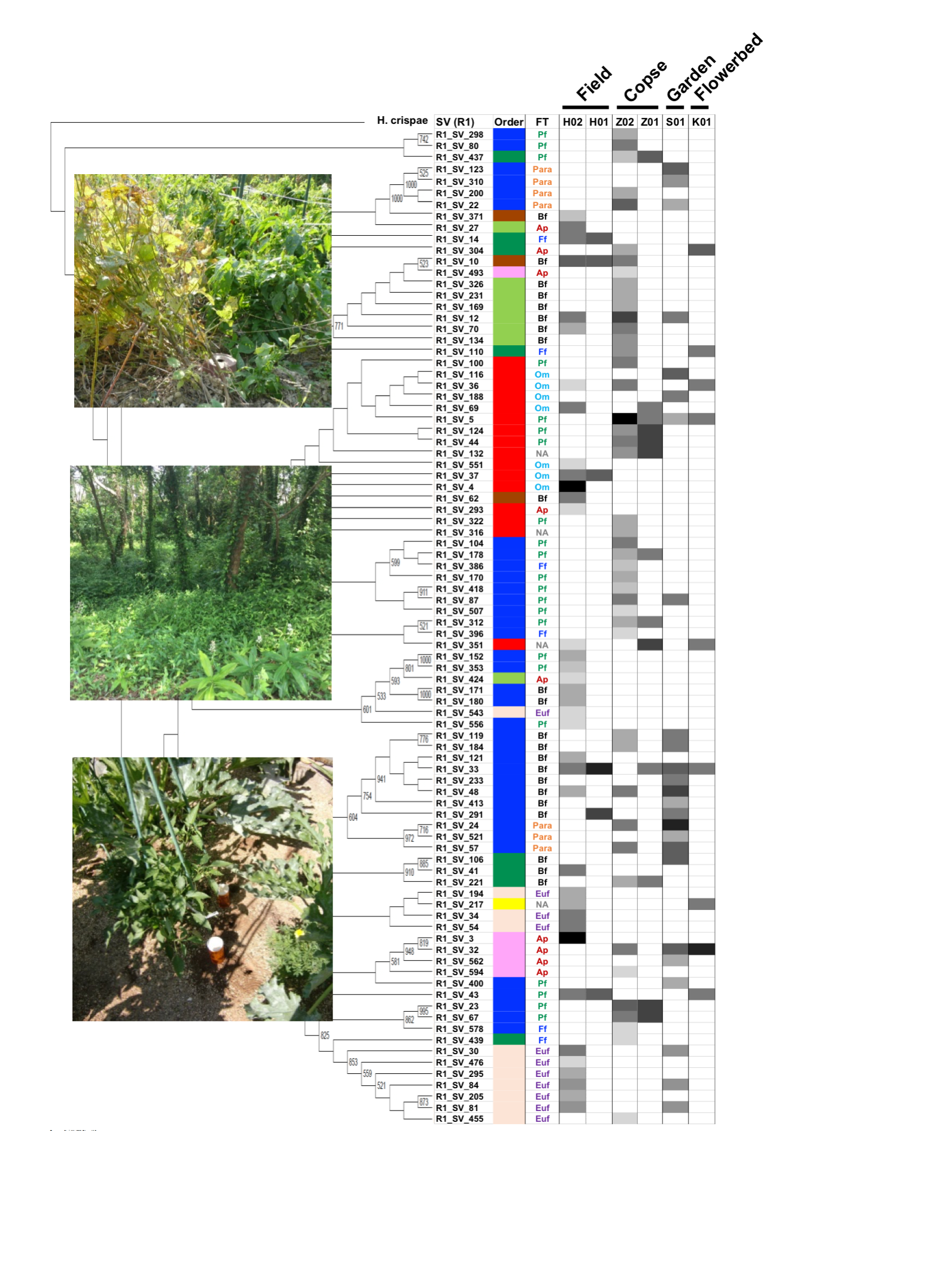

Supplement: S11 Fig — (TIFF) [file pone.0249571.s022.tiff]
