# Supplementary material for: Distinct community structures of soil nematodes from three ecologically different sites revealed by high-throughput amplicon sequencing of four 18S ribosomal RNA gene regions
Source: PLoS One. 2021 Apr 15;16(4):e0249571. doi: 10.1371/journal.pone.0249571 (PMC8049254; doi:10.1371/journal.pone.0249571)
Supplement: S1 Raw images — The amplification of each SSU gene region of nematode DNA from the copse (left image of the first figure) and house garden (right image) soils was performed by PCR with tailed PCR primers for each SSU gene region. Aliquots of the resultant PCR products from each sample were independently subjected to 1% agarose gel electrophoresis as shown in the figure, where a red “X” designates an empty lane. The PCR products from one-step PCR (right side of the second figure) and two-step PCR (left side of the second figure, including a marker lane) were amplified using the field sample and were subjected to 2% agarose gel electrophoresis. The PCR products in the three gels were visualized using successive ethidium bromide staining. Fluorescent images of agarose gels were acquired using the FAS-III gel imaging system (Nippon Genetics Co., Tokyo, Japan), and the original TIFF images shown in each file were used to prepare S3A–S3C Fig by cropping out the unrelated area. M: lane with a size marker (Gene Ladder Wide 1, Nippon Gene, Toyama, Japan). (PDF) [file pone.0249571.s023.pdf]

S1 Raw images.

Below are the raw gel images used to create S3A and S3B Figs (left and right, respectively). These images were only modified by cropping to remove the empty or unrelated (separate PCR run on the same gel) lanes, as marked with a red "X".

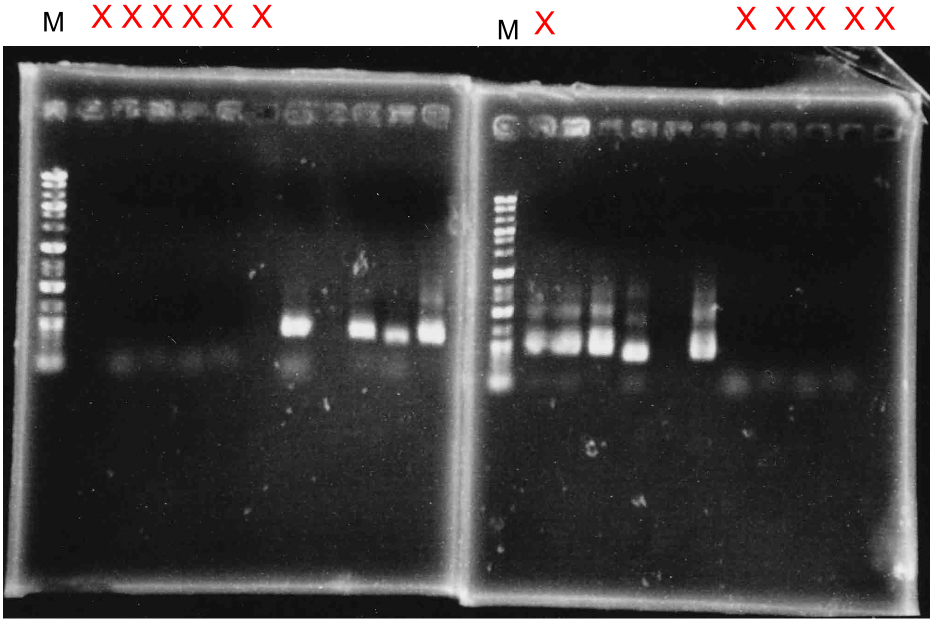

The house garden soil samples are shown in the left image. Here, lane 1 contained the size marker (M; Gene Ladder Wide 1, Nippon Gene, Toyama, Japan). Lanes 2, 7, and 9 were empty. Lanes 3–6 contained the negative control for PCR without a template DNA. Lanes 8, 10, 11, and 12 contained PCR products indicated in the legend of S3 Fig. The copse samples are shown in the right image. Here, lane 1 contained the size marker (M). Lane 2 contained the same PCR product as in lane 3, owing to leakage from lane 3. Lanes 6 and 12 were empty. Lanes 8–11 contained the negative control products Lanes 3, 4, 5, and 7 contained the PCR products indicated in the legend of S3 Fig. The PCR products were subjected to 1% agarose gel electrophoresis and visualized using successive ethidium bromide staining. The fluorescent images of agarose gels were taken using the FAS-III gel imaging system (Nippon Genetics Co., Tokyo, Japan). The photograph was scanned by the image scanner (TS8230, Canon) to generate a PDF file.

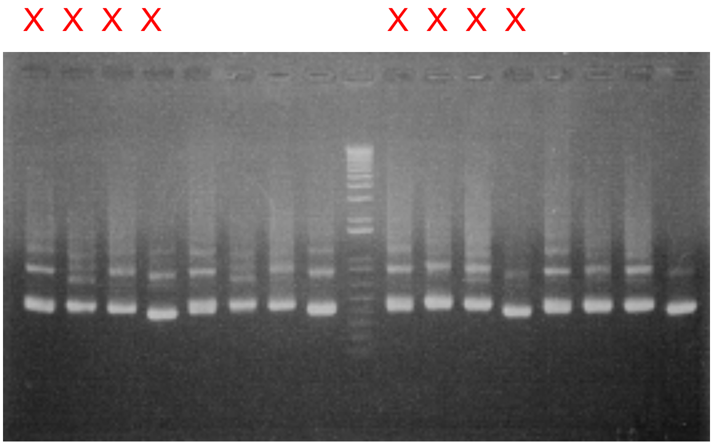

Shown above is the uncropped image for S3C Fig. Lanes 1–4 and 10–13 contained PCR products from unrelated reactions containing different template DNAs. Lane 9 contained the size marker (M). Lanes 5–8 and 14–17 contained PCR products indicated in the legend of S3 Fig. The PCR products were separated in 2% agarose gel electrophoresis. The ethidium bromide-stained gel image was acquired using the FAS-III imaging system to generate a TIFF image file.
